# Electric‐Fish‐Inspired Thin Hydrogel Electrocytes Achieve High Power Density and Environmental Robustness

**DOI:** 10.1002/advs.202519348

**Published:** 2025-12-07

**Authors:** Dor Tillinger, Wonbae Lee, Haley M. Tholen, Derek M. Hall, Joseph S. Najem

**Affiliations:** ^1^ Department of Mechanical Engineering The Pennsylvania State University 336 Reber Building University Park PA 16802 USA; ^2^ Department of Materials Science and Engineering The Pennsylvania State University Steidle Building University Park PA 16802 USA; ^3^ Department of Mechanical Engineering The Pennsylvania State University 166 Energy and Environment Laboratory University Park PA 16802 USA

**Keywords:** anti‐freezing, bioinspired power sources, compact power sources, electric‐fish‐inspired power sources, environmental stability, hydrogels, layer‐by‐layer spin coating, scalable fabrication, sustained hydration

## Abstract

Electric‐fish‐inspired hydrogel‐based power sources offer a promising platform for powering soft, wearable, and implantable electronics due to their compliance, biocompatibility, and biodegradability. They typically consist of high‐ and low‐salinity gel layers separated by anion‐ and cation‐selective gel compartments, generating an electric potential that emulates the diffusion‐based energy mechanisms of electrocytes in electric fish. However, their development has been hindered by high internal resistance, limited power density, and poor environmental stability. Here, a scalable layer‐by‐layer spin‐coating strategy is introduced to fabricate hydrogel electrocytes with precise thickness control, yielding 106.1 µm‐thick units comparable to biological electrocytes. This thin architecture significantly reduces resistance and enables high instantaneous power density (44.0 kW m^−3^) with low area‐normalized resistance (2.0 × 10^−3^ Ω m^2^.). By tailoring the hydrogel composition with a glycerol–carboxylated chitosan mixture, long‐term hydration (>98.7% after 120 h at 60% RH) and antifreezing performance down to −80 °C are achieved without encapsulation. Furthermore, varying layer thickness provides tunable energy density, while integration of PEDOT:PSS hydrogel electrodes preserves material compliance and yields robust, ready‐to‐use power systems. These advances overcome critical barriers in hydrogel‐based energy storage, establishing a versatile, scalable pathway toward stable, bioinspired power sources for next‐generation wearable, implantable, and autonomous devices.

## Graphical abstract

This study presents thin, environmentally stable hydrogel power sources inspired by electric fish. Made using layer‐by‐layer spin‐coating with glycerol‐enhanced solutions, they offer precise layer control, long‐term hydration, and anti‐freezing stability. The devices achieve a high‐power density of 44 kW m^‒3^, low resistance, and are biocompatible, making scalable, wearable, and implantable hydrogel‐based energy systems are possible.

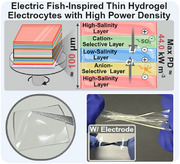

## 1 Introduction

The ongoing advancement and miniaturization of modern electronics require power sources that combine compactness, scalability, and flexibility with reliable performance under both harsh and sensitive conditions.^[^ ^1^, ^2^ ^]^ Emerging applications such as soft robotics, implantable biomedical devices, and wearable electronics further demand attributes beyond electrical output, including biocompatibility, safety, mechanical compliance, and lightweight construction.^[^ ^1^, ^3^, ^4^, ^5^ ^]^ Current commercial power systems, such as lithium‐ion (Li‐ion) batteries, were not designed with these capabilities in mind. Although Li‐ion batteries remain the most widely used technology due to their high power and energy densities and long cycle life,^[^ ^6^ ^]^ their toxic,^[^ ^3^, ^7^ ^]^ flammable,^[^ ^4^, ^5^ ^]^ rigid,^[^ ^1^, ^7^, ^8^ ^]^ and heavy components^[^ ^5^ ^]^, limit compatibility with deformable, lightweight, or miniaturized platforms.^[^ ^1^, ^8^ ^]^

In response, researchers have explored various modifications to Li‐ion batteries, such as polymer substitution, structural redesigns, and alternative fabrication methods, improving performance, safety, and stability.^[^ ^8^, ^9^, ^10^, ^11^ ^]^ Yet, many of these systems retain the fundamental drawbacks of conventional Li‐ion batteries.^[^ ^8^, ^12^ ^]^ Alternative chemistries beyond lithium‐ion also face challenges related to mechanical rigidity, safety, and material availability.^[^ ^9^, ^12^ ^]^ These limitations underscore the need for entirely new classes of power systems that are compliant, soft, safe, and structurally compatible with next‐generation electronics.

**Figure 1:**
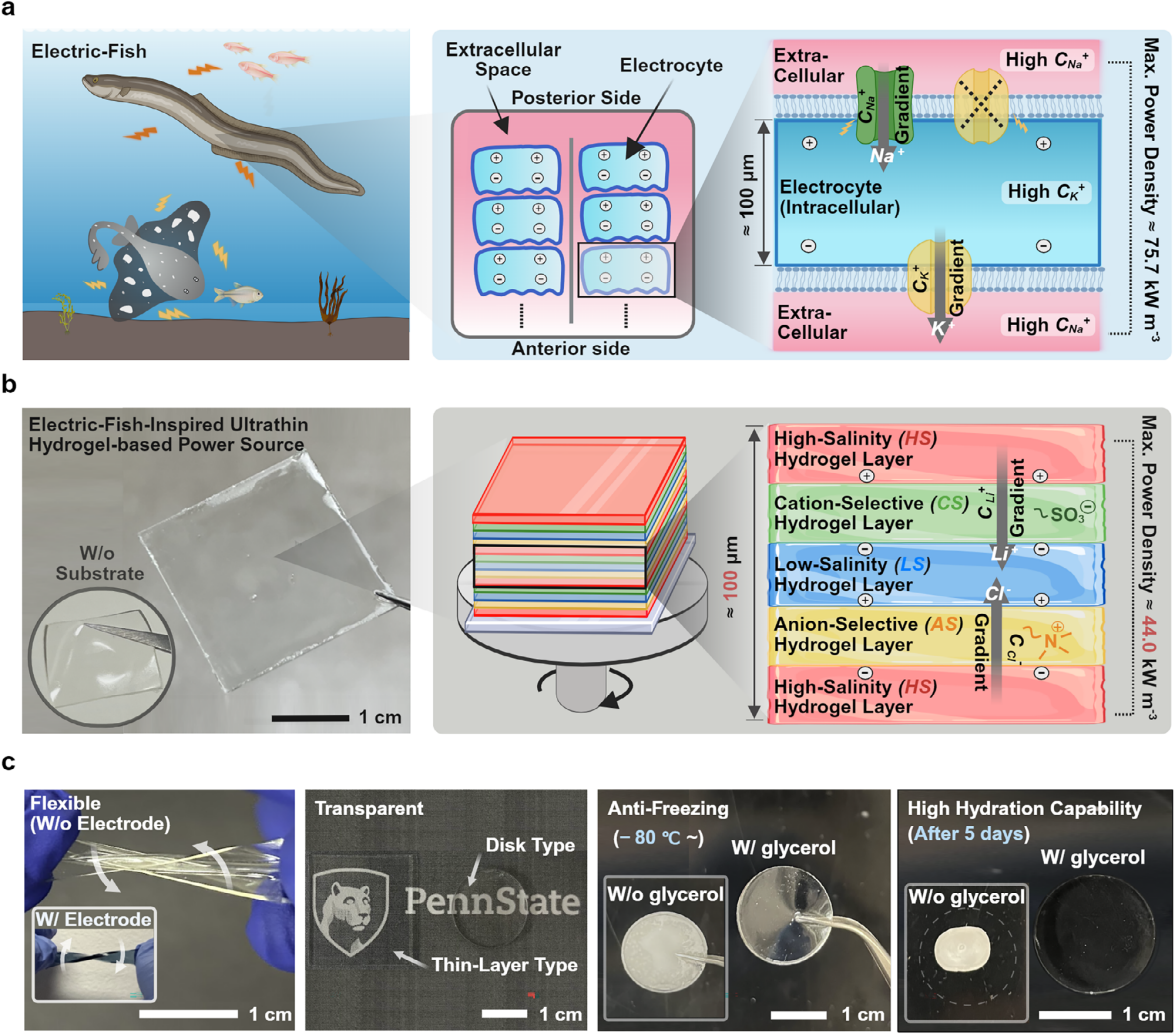
a) Schematic of voltage generation in electrocytes of electric‐fish, where asymmetric ion gradients and selective ion channels across anterior and posterior sides enable transcellular potential generation. b) Photograph and schematic of the electric‐fish‐inspired thin hydrogel‐based power source. The design mimics the electrocyte potential generation mechanism by layering high‐salinity (HS), anion‐selective (AS), low‐salinity (LS), and cation‐selective (CS) hydrogels to establish a concentration gradient and charge separation. Our thin 100‐µm‐thick electrocyte allows for maximum power density of 44.0 kW m^−3^ c) Key material advantages of our hydrogel compositions, including mechanical flexibility, optical transparency, anti‐freezing capability down to –80°C, and long‐term hydration.

One promising alternative draws inspiration from the electrical generation mechanisms of electric fish, leading to the development of hydrogel‐based power sources.^[^ ^13^, ^14^, ^15^, ^16^ ^]^ Species such as *Electrophorus electricus* (electric eel) and *Tetronarce nobiliana* (torpedo ray) possess electric organs comprising specialized cells called electrocytes, which generate electric potentials by maintaining asymmetric ion concentrations between their intracellular and extracellular environments.^[^ ^17^, ^18^ ^]^ At rest, the anterior and posterior membrane potentials cancel, yielding no net voltage. Upon stimulation, sodium channels in the posterior membrane open, allowing Na^+^ influx, while potassium channels close, maintaining ionic separation and producing a transcellular voltage (**Figure** **1**a).^[^ ^17^, ^18^ ^]^ A single electrocyte in an electric eel generates ≈130 mV, with an average thickness of ≈100 µm and cross‐sectional area of 40 mm × 1.5 mm.^[^ ^13^, ^19^ ^]^ By stacking thousands of electrocytes in series, the eel can produce more than 600 V and deliver peak power up to 100 W, all driven by ionic concentration gradients.^[^ ^13^, ^18^, ^20^, ^21^, ^22^ ^]^

Mimicking this biological architecture, hydrogel‐based power sources replicate the function of the electrocyte using four distinct hydrogel types.^[^ ^13^ ^]^ Two salinity hydrogels, one high‐salinity (HS) and one low‐salinity (LS), represent the extracellular and intracellular environments, respectively (Figure 1b). The other two are selective layers: a cation‐selective (CS) hydrogel that permits cation transport and an anion‐selective (AS) hydrogel that permits anion transport. Each selective layer contains a fixed‐charge polymer backbone that repels co‐ion flux and facilitates counter‐ion flux, mimicking selective ion channels embedded in the electrocyte membranes that regulate ion‐specific transport across the cell. Stacking these four hydrogel types in the HS–AS–LS–CS–HS sequence forms a unit assembly that produces an ionic concentration gradient and voltage output analogous to that of a biological electrocyte.^[^ ^13^ ^]^ Hydrogel‐based power sources also offer intrinsic material advantages, including biocompatibility, flexibility, light weight, compliance, shape adaptability, transparency, and cost‐effectiveness.^[^ ^12^, ^13^, ^23^, ^24^ ^]^ These attributes make them compelling candidates for next‐generation power systems, particularly in miniaturized biomedical and robotic electronic applications.^[^ ^12^, ^24^ ^]^

The proof‐of‐concept system presented by Schroeder et al.^[^ ^13^ ^]^ employed printed hydrogel units in an origami‐folded configuration. Each unit generated an instantaneous maximum open‐circuit potential (OCP, V_oc_) of ≈170 mV, while serial stacking of 614 units produced 110 V. However, the device exhibited a high area‐normalized resistance and a low volumetric power density of 0.0096 kW m^−3^ (**Table** **1**), limiting its practical utility. Subsequent studies pursued material and structural optimization via alternative fabrication approaches. For instance, Guha et al.^[^ ^14^ ^]^ investigated various salt types and concentrations to increase OCP. Additionally, the researchers redesigned the geometry using thin sheets of lens‐cleaning paper as a porous scaffold for each hydrogel layer, improving mechanical integrity by forming a paper‐gel assembly. Geometrically, instantaneous maximum power output ($P_{max}=\frac{V_{OC}^{2}}{4R}$) is achieved by minimizing internal resistance ($R=\frac{\rho T}{A}$) through reducing the thickness‐to‐area ($\frac{T}{A}$) aspect ratio (Section S9.1, Supporting Information).^[^ ^25^ ^]^ Using this approach, Guha et al.^[^ ^14^ ^]^ improved instantaneous maximum volumetric power density to 1.3 kW m^−3^. Nonetheless, the porous scaffold impeded ion transport and limited the minimum unit thickness to 1.4 mm, constraining further reductions in area‐normalized resistance and hindering miniaturization. Meanwhile, Zhang et al.^[^ ^15^ ^]^ applied the droplet interface bilayer (DIB) method to achieve volumetric miniaturization and structural complexity. While instantaneous maximum power density remained comparable to the paper‐gel assembly, the process is labor‐intensive and complex,^[^ ^12^, ^15^ ^]^, limiting scalability. Moreover, the droplet‐based geometry imposed a constraint in which reducing the thickness inherently decreased the cross‐sectional area, as the droplet diameter govern both dimensions. This coupling resulted in a relatively large aspect ratio, thereby limiting further geometric refinement and resistance reduction.^[^ ^15^ ^]^ Specifically, the power density of current systems is still at least one order of magnitude lower compared to their biological counterparts (Table 1). Despite these advances, substantial opportunities remain to improve the performance and design of hydrogel‐based power sources. Moreover, environmental stability, particularly long‐term hydration and anti‐freezing capacity, remains insufficiently addressed, despite its critical role in maintaining electrical performance and meeting requirements for wearable, implantable, or outdoor applications.^[^ ^12^, ^24^ ^]^ These challenges underscore the need for integrated design strategies that unify scalable fabrication, geometrical control, and material optimization to realize high‐performance, application‐ready hydrogel power systems.

**Table 1:** Performance metrics of biological electrocytes and electric‐fish‐inspired hydrogel power sources.

Source^a)^	Thickness of unit^a,b)^[µm]	Volume of unit^a,b)^[mm^3^]	Max open‐circuit potential per unit^a,b)^[mV]	Area‐normalized internal resistance per unit^a,b)^[Ω mm^2^]	Internal resistance per unit^a,b)^[Ω]	Maximum power density by unit^a,b)^[kW m^−3^]
Electric eel^e)^ (*Electrophorus electricus*)^c)^ ^[^ ^14^, ^19^, ^21^, ^26^, ^27^ ^]^	100	6.0	130	560	9.3	76
Torpedo ray^e)^ (*Tetronarce nobiliana*)^d)^ ^[^ ^14^, ^28^ ^]^	20	0.56	60	32	1.1	1500
Origami assembly,^e)^ Schroeder et al.^[^ ^13^ ^]^	2800	270	170	270000	3200	0.0096
Paper‐gel assembly,^e)^ Guha et al.^[^ ^14^ ^]^	1400	320	240	8000	36	1.3
Droplet assembly (1.84~nL),^e)^ Zhang et al.^[^ ^15^ ^]^	750	0.0092	86	1800	150000	1.3
Microfluidic assembly,^e)^ He et al.^[^ ^16^ ^]^	48000	620	140	95000	7300	0.0010
**Scaffold‐free thin layer assembly,** (**This work**)	**110**	**18**	**190**	**2000**	**12**	**44**

a) Unit refers to a single electrocyte in an electric fish or a single assembly of the four hydrogel types required to generate potential.

b) All values are rounded to 2 significant figures.

c) Mean values were derived from Guha et al.,^[^ ^14^ ^]^ which compiles data from three studies on *Electrophorus electricus*.^[^ ^21^, ^26^, ^27^ ^]^. Volume was estimated using a cross‐sectional area of 40~mm × 1.5~mm from Gotter et al.^[^ ^19^ ^]^

d) Values for *T. nobiliana* were estimated by approximating each electrocyte as a disc (6~mm diameter, 20~µm thick), following assumptions in Guha et al.^[^ ^14^ ^]^

e) Calculated from reported data or averaged across references.

One fabrication method well‐suited to addressing these needs is spin coating, which offers scalability, simplicity, reproducibility, and rapid deposition of thin polymeric layers.^[^ ^29^, ^30^, ^31^, ^32^ ^]^ In spin coating, centrifugal force uniformly distributes a polymeric precursor over a substrate,^[^ ^33^, ^34^ ^]^, and the final film thickness can be precisely tuned by adjusting parameters such as spin speed, spin time, and precursor rheology.^[^ ^33^, ^34^ ^]^ This control enables the fabrication of layers ranging from a few micrometers to several hundred micrometers, making spin coating an attractive fabrication method for hydrogel‐based power sources, where geometric optimization is critical to lowering internal resistance.^[^ ^33^, ^34^ ^]^ While spin coating is traditionally used for depositing single polymer layers, this technique has recently been adapted into layer‐by‐layer (LbL) processes for applications in medical dressings, flexible electronics, and antimicrobial coatings, enabling construction of multilayer assemblies with tailored architectures.^[^ ^29^, ^30^ ^]^

In this work, we fabricate thin, scaffold‐free, and environmentally stable hydrogel‐based power sources through an LbL spin coating procedure (Figure 1b,c). We use a binary solvent system composed of water and glycerol to enhance fabrication feasibility and impart long‐term hydration (>98.7% after 120 h at 60% RH) and anti‐freezing capabilities (down to −80 °C), ensuring consistent performance under diverse operating conditions. This approach enables precise control and reduction of the thickness of the unit assembly close to biological values (to 106.1 µm), thus lowering internal resistance and volume to maximize power output (44.0 kW m^−3^) (Table 1). We further investigate the influence of individual layer thicknesses on key performance metrics, such as energy density, and incorporate PEDOT:PSS hydrogel electrodes to yield ready‐to‐use, integrable hydrogel‐based power systems. Overall, this work introduces an innovative fabrication strategy that integrates material optimization with geometric control, offering a promising path toward high‐performance energy platforms for next‐generation wearable electronics, biomedical devices, soft robotics, and energy storage systems.

## 2 Fabrication of Scaffold‐Free Thin Hydrogel Assemblies

Layer‐by‐layer (LbL) spin coating involves the sequential deposition and polymerization of hydrogel layers onto a previously cured hydrogel base.^[^ ^29^, ^32^, ^35^, ^36^ ^]^ Most reported techniques rely on electrostatic attraction as the dominant adhesion mechanism between layers, which requires alternating positively and negatively charged layers.^[^ ^30^, ^37^ ^]^ While this approach is effective for polyelectrolyte assemblies, it is incompatible with hydrogel‐based power sources, where electrostatic interactions disrupt ion transport and compromise the concentration gradients necessary for generating electric potential. Recently, Zheng et al.^[^ ^29^ ^]^ demonstrated a non‐electrostatic LbL spin coating strategy by physically crosslinking two types of hydrogel using acrylic acid as copolymers. By partially curing the first layer before fully curing the second, they were able to fabricate thin bilayer hydrogel films intended for soft actuators. However, this method proves difficult to extend to multilayer architectures (⩾ 3 layers) due to the need for tightly controlled curing sequences and compositional compatibility. This limitation restricts the types of hydrogels that can be used and the control over layer thicknesses. Hydrogel‐based power sources require at least four functionally distinct layers to establish ionic gradients and regulate electrical output. Therefore, a modular fabrication strategy is necessary, in which each layer can be fully cured and independently controlled.

**Figure 2:**
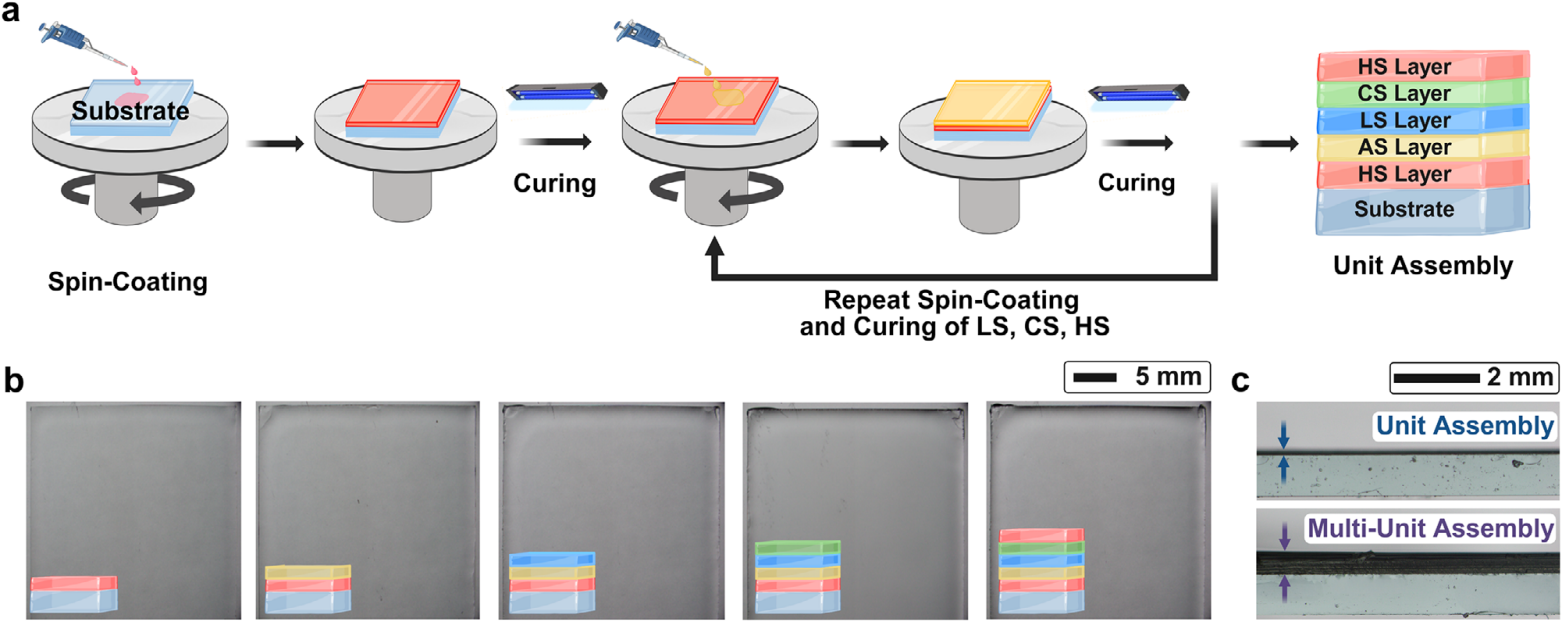
Process of layer‐by‐layer (LbL) spin coating and optical images of electrocyte units. a) Schematic illustration of the spin‐coating and in situ polymerization steps used to sequentially build hydrogel layers into an electrocyte unit assembly. b) Optical surface images showing progressive layer accumulation during successive spin‐coating cycles, confirming uniform film formation. c) Cross‐sectional optical microscopy images of a single‐unit and multi‐unit assemblies, demonstrating the scalability of the spin‐coating approach. Note that the cartoons representing the hydrogel layers are not to scale.

For our LbL spin coating fabrication, we employ photopolymerization, a simple and rapid curing method, to construct multilayer structures with enhanced structural integrity.^[^ ^31^, ^36^, ^38^ ^]^ The process begins by spin coating the first precursor solution, the HS layer, onto a glass substrate, followed by UV‐induced photopolymerization to covalently crosslink the layer (see Experimental Section for more details). The next precursor solution, the AS layer, is then deposited directly on top of the cured HS layer and photopolymerized (**Figure** **2**a; see Section S1, Figure S1, Supporting Information for more details). This process is repeated for each functional hydrogel, with each polymerized layer serving as the substrate for the next precursor solution, yielding a thin, smooth hydrogel‐based soft power source (Figure 2b). In situ photopolymerization facilitates covalent crosslinking both within and between layers via acrylamide chains, producing robust interlayer adhesion, enhanced mechanical strength, and reduced contact resistance,^[^ ^31^, ^36^ ^]^ all of which are critical for high‐performance ionic conduction. Cross‐sectional microscopy images of single‐unit and multi‐unit assemblies confirm the stacking of uniform layer thickness and scalability of our LbL fabrication (Figure 2c).

## 3 Tuning Hydrogel Composition for Enhanced Mechanical Properties, Hydration, and Anti‐Freezing Capabilities

**Figure 3:**
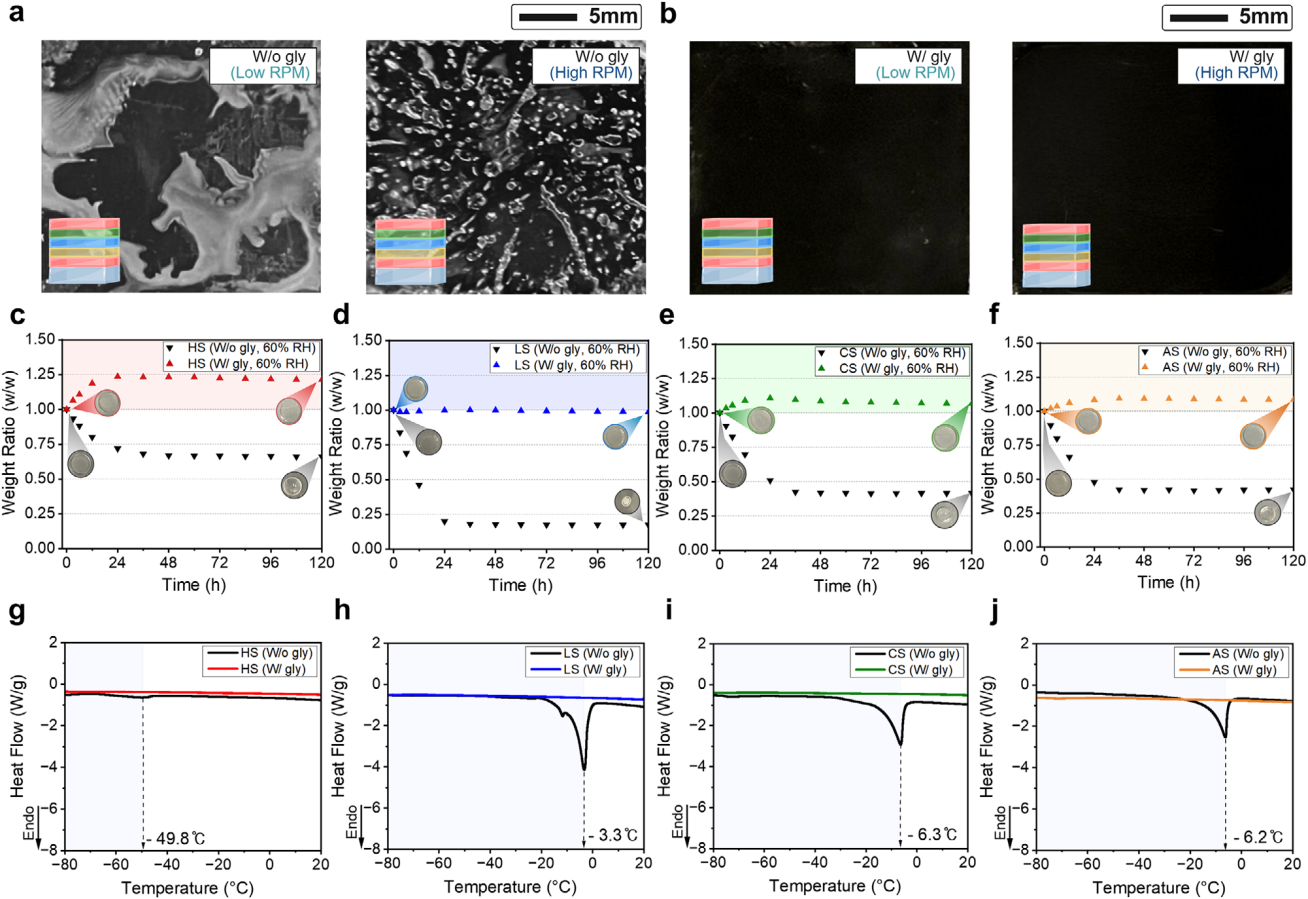
Comparison of fabrication feasibility and environmental stability between single‐ (water only) and binary‐ (water–glycerol mixture) solvent hydrogel systems. a) Optical images of unit assemblies fabricated using a single‐solvent system at low and high RPM, showing poor surface uniformity due to low solution viscosity and dehydration. b) Assemblies fabricated using a binary‐solvent system at low and high RPM, illustrating smooth surfaces and suppressed dehydration, demonstrating improved fabrication feasibility. c–f) Mass change of the four hydrogel types over 120 h at 25 ± 5 °C and 60 ± 5% RH. Binary‐solvent hydrogels (colored markers) show increased hydration compared to their single‐solvent counterparts (black markers), with retention values of 121.3% (HS), 98.7% (LS), 106.7% (CS), and 108.5% (AS) for binary‐solvent gels, and 66.0%, 17.0%, 41.4%, and 42.1% for single‐solvent gels. Insets display dimensional changes, highlighting shrinkage in single‐solvent hydrogels (up to −50.0%) and negligible deformation in binary‐solvent systems (⩽5.6%) (Figure S11 shows zoomed‐in images of the insets). g–j) Differential scanning calorimetry (DSC) curves of the hydrogels from −80 to 20 °C. Binary‐solvent hydrogels exhibit no clear phase transition peak, indicating superior anti‐freezing behavior, while single‐solvent systems show distinct endothermic peaks in the same temperature range.

The success of spin coating thin hydrogel layers requires balancing process parameters such as spin speed and spin time with solution properties, including viscosity and surface tension relative to the substrate.^[^ ^32^, ^33^, ^39^, ^40^ ^]^ In general, higher spin speeds, longer spin durations, and lower viscosities yield thinner layers.^[^ ^32^, ^33^, ^41^ ^]^ However, if the viscosity is too low, a factor strongly influenced by solution composition and substrate material, uncontrolled spreading and rapid solvent evaporation can occur, leading to non‐uniform coatings.^[^ ^32^, ^42^ ^]^ In our initial LbL spin coating attempts, such instabilities produced irregular films that hindered reliable multilayer stacking (**Figure** **3**a). To address this issue, we evaluated strategies commonly used to improve spin‐coating uniformity, including tuning solution viscosity by adjusting polymer concentration or incorporating additives.^[^ ^39^, ^40^ ^]^

     We selected to incorporate glycerol as an additive because it simultaneously improves spin‐coating feasibility by increasing solution viscosity while enhancing hydration and depressing the freezing point of the material.^[^ ^43^, ^44^ ^]^ Glycerol is a hydrophilic, hygroscopic, biodegradable, non‐toxic, water‐miscible solvent with high polarity and three hydroxyl groups, giving it a high intrinsic viscosity.^[^ ^43^, ^44^, ^45^, ^46^, ^47^, ^48^, ^49^ ^]^ Further, its hygroscopic nature promotes uniform film formation by absorbing and retaining environmental moisture, thus preventing dehydration during and after spin coating the electrocyte assembly.^[^ ^43^, ^44^, ^46^, ^47^, ^48^, ^49^ ^]^ For hydrogel‐based power sources, sustained hydration is essential to stabilize ionic resistance and maintain electrical performance.^[^ ^50^ ^]^ Beyond moisture retention, glycerol's hydroxyl groups form strong hydrogen bonds with water, disrupting water‐water bonding networks, enhancing hydration, and inhibiting ice crystallization.^[^ ^46^, ^49^ ^]^ Glycerol also improves mechanical and dimensional stability through interactions with amide groups in polyacrylamide, the primary polymer in all gel types (see Experimental Section).^[^ ^51^ ^]^ Thus, incorporating glycerol into a binary water–glycerol solvent enables reliable LbL spin coating of hydrogel power sources while ensuring long‐term hydration and freezing resistance under harsh conditions, without encapsulation.

Our finalized hydrogel compositions contain 65 % (v/v) glycerol in the LS and HS hydrogels and 70 % (v/v) glycerol in the CS and AS hydrogels. Section S2 (Supporting Information) (Figures S2– S6, Supporting Information) details the parametric study we conducted to identify these formulations. One drawback of including glycerol into our hydrogel precursor solutions is that the inherent increase in viscosity to enable LbL fabrication will lead to an increase in resistivity^[^ ^52^, ^53^ ^]^, reducing the electrical output of the hydrogel power source. Therefore, the finalized LS hydrogel composition included carobxylated chitosan (CCS) as the LS hydrogel dominates the hydrogel power source unit resistivity (Figure S7, Table S1, Supporting Information). CCS is a biocompatible, water‐soluble polymer known to increase the conductivity of hydrogels,^[^ ^54^, ^55^ ^]^ which upon incorporation into the LS hydrogel reduces the unit resistivity from 19.6 to 18.2 Ωm by reducing the energy barrier for ion transport (More information in Section S2.3.1 and Figure S8, Supporting Information).^[^ ^54^ ^]^ Adding glycerol and CCS into our finalized compositions promotes LbL fabrication of scaffold‐free thin hydrogel power sources by increasing viscosity and augmenting conductivity through material formulation.

To directly assess the effect of glycerol on hydrogel uniformity, we fabricate unit assemblies using a single‐solvent (water only) system and binary‐solvent (water‐glycerol) system. Figure S9 (Supporting Information) illustrates the effect of glycerol on the viscosity of the hydrogel precursors. Following fabrication, samples were incubated at 25 ± 5 °C and 60 ± 5% relative humidity (RH) for 10 min and visually inspected for uniformity at each step (Figure S10, Supporting Information). Assemblies made with the single‐solvent system produced highly nonuniform layers at all spin speeds, caused by uneven spreading from low viscosity and rapid evaporation (Figure 3a). We further hypothesize that dehydration during stacking caused the aqueous precursor solutions to be absorbed by the underlying dehydrated polymerized hydrogel layers, producing additional defects such as beading. In contrast, assemblies fabricated with the binary‐solvent system produced smooth, well‐defined multilayer structures (Figure 3b). With glycerol as a co‐solvent, precursor solutions and polymerized hydrogels remained hydrated and environmentally stable through fabrication, enabling uniform spreading across substrates and successive layers.

In addition to fabrication feasibility, long‐term hydration stability is essential for maintaining reliable ionic conductivity^[^ ^50^ ^]^ and mechanical integrity^[^ ^49^, ^56^ ^]^ in hydrogel‐based power systems. Hydrogels are inherently prone to water loss through evaporation under ambient or dry conditions, and dehydration reduces the availability of mobile ions while compromising structural stability, leading to deformation or wrinkles.^[^ ^57^, ^58^, ^59^ ^]^ These effects reduce the ionic conductivity and degrade overall electrochemical performance. We evaluate the hydration stability by monitoring the mass of each hydrogel type fabricated using either a single‐ or binary‐solvent system over 5 days at 25 ± 5 °C in both 60 ± 5% and 11 ± 1% RH (see Experimental Section and Figures S11 and S12, Supporting Information). Cast disk samples (18 mm diameter, 1.8 mm thick) were used to evaluate hydration, since single‐solvent hydrogels did not spin‐coat uniformly and were therefore challenging to analyze. We observed that single‐solvent gels dehydrated rapidly, losing most of their water content within 24 h at 60 % RH. In contrast, binary‐solvent gels retained or increased their hydration over 120 h, with final mass retention exceeding 98% (Figure 3c‐f). Geometrical measurements further confirmed that binary‐solvent gels maintained their structure (diameter change ⩽ 5.6%), whereas single‐solvent gels shrank by up to 50.0% (Figure S11a, Supporting Information). Notably, the HS hydrogel exhibited particularly high hydration retention due to its elevated lithium chloride (LiCl) content, a hygroscopic salt that promotes environmental moisture uptake.^[^ ^60^ ^]^

Under low‐humidity conditions (11 ± 1% RH), the binary‐solvent gels retained 78.5−86.7% of their initial mass, whereas single‐solvent gels retained only 16.4−42.4% of their initial mass (Figure S12, Supporting Information). Reduced hydration in binary‐solvent gels is attributed to the limited ambient moisture, which restricted uptake by the hygroscopic components. Nonetheless, even in low‐humidity environments, binary‐solvent gels preserved their geometry (0−5.6% shrinkage) (Figure S11b, Supporting Information), supporting consistent and reproducible electrical performance.

Finally, many environmentally stable bioelectronics and sensors require resilience to both dehydration and freezing,^[^ ^49^, ^56^ ^]^ demanding our hydrogel‐based power sources to sustain reliable electrical performance at subzero temperatures. Conventional hydrogels, which use water as the sole solvent, freeze under such conditions,^[^ ^49^, ^61^ ^]^ drastically reducing ionic conductivity and electrochemical performance.^[^ ^61^, ^62^ ^]^ To assess the anti‐freezing capabilities of both the single‐solvent and binary‐solvent hydrogels, we conducted differential scanning calorimetry (DSC) across a temperature range of −80 to 20 °C (Figure 3g–j). Single‐solvent hydrogels exhibited distinct thermal transition peaks at −49.8 °C (HS), −3.3 °C (LS), −6.3 °C (CS), −6.2 °C (AS), confirming their susceptibility to freezing. We found that the single‐solvent HS hydrogel had the lowest freezing point compared to the other single‐solvent gels, which we attribute to the high concentration of LiCl as the large hydration shells of Li^+^ ions disrupt hydrogen bonding.^[^ ^63^, ^64^ ^]^ The single‐solvent LS hydrogel, which incorporates carboxylated chitosan (CCS) (see Experimental Section) to reduce ionic resistivity (Figure S8, Supporting Information), displayed two distinct thermal peaks (Figure 3h). This secondary peak may arise from interactions between CCS hydrophilic groups and water molecules, promoting bound water formation and contributing to the complex DSC profile.^[^ ^54^ ^]^ In contrast, all binary‐solvent hydrogels exhibited no distinct phase transitions across the entire temperature range, demonstrating complete anti‐freezing capabilities and eliminating the need for encapsulation (visually demonstrated in Figure S13, Supporting Information). Furthermore, we compare the ionic resistivity of each hydrogel at room and sub‐zero temperatures. We observe that for all gel types, including the unit stack to form a complete power source, the ionic resistivity increased at subzero temperatures due to slower ionic mobility (Figure S14, Supporting Information).^[^ ^61^ ^]^ Despite the increase in ionic resistivity, our hydrogel power source still produces a maximum open circuit potential of 162 ± 1 mV at subzero temperatures, ≈30 mV less than at room temperature (Figure S14, Supporting Information).

## 4 Identifying Spin‐Coating Parameters to Control Hydrogel Layer Thickness

Building on our finalized hydrogel compositions, we next focused on fabricating hydrogel‐based power sources through LbL spin coating. While previous studies have demonstrated thin‐layer hydrogel fabrication,^[^ ^14^, ^29^, ^41^, ^65^ ^]^ precise control over individual layer thickness remains a challenge. In our approach, layer thickness can be systematically tuned by adjusting spin‐coating parameters such as spin speed (*vs*) and spin time (*ts*). Because the final thickness of each layer also depends on the hydrogel composition and the underlying substrate (*us*), experimentally determined spin curves are essential to understand how processing parameters influence film formation.^[^ ^39^ ^]^ In general, the hydrogel layer thickness decreases exponentially with increasing spin speed and time until a minimum saturation thickness is reached.^[^ ^29^ ^]^

**Figure 4:**
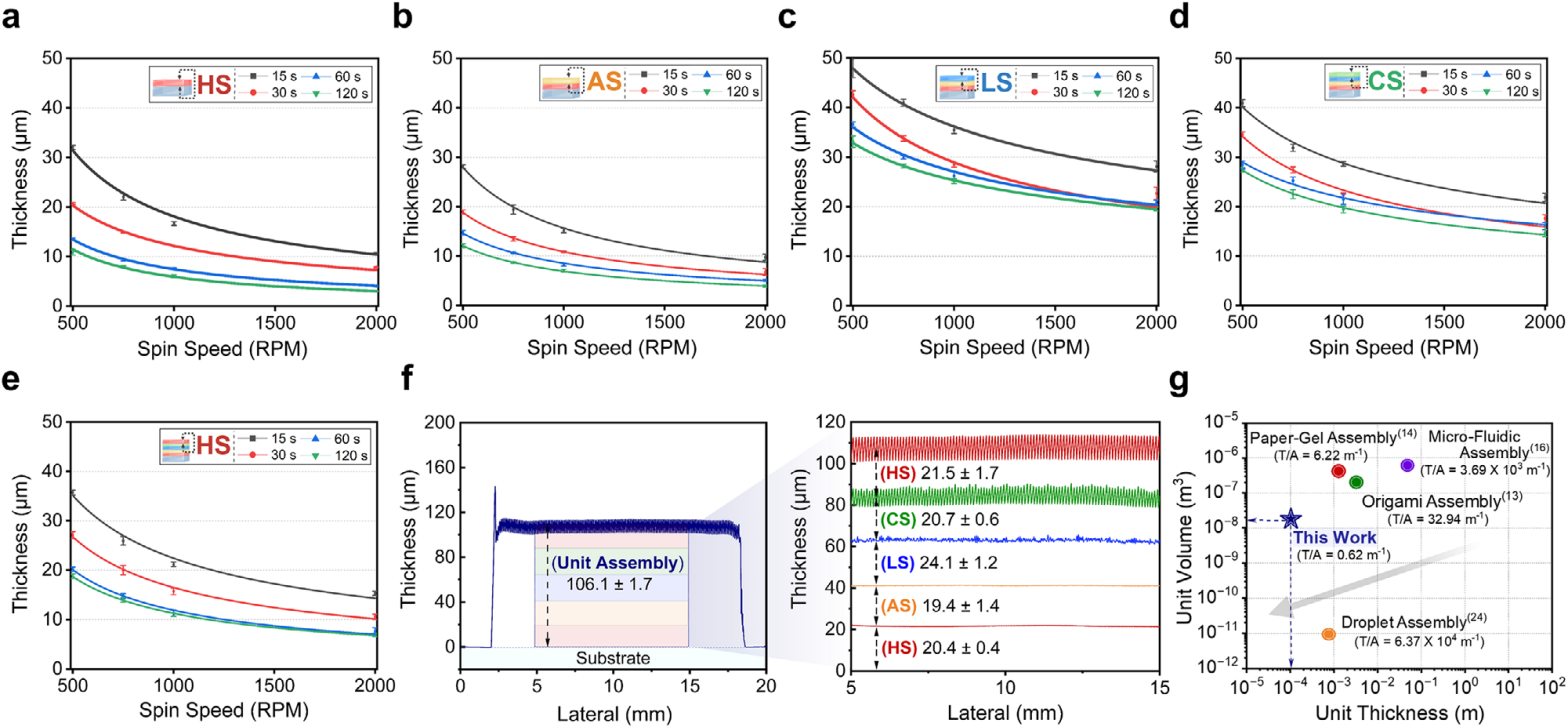
a–e) Spin curves for each hydrogel layer as a function of spin speed and spin time, showing thickness control with spin coating parameters. The thicknesses are presented as mean ± standard deviation (n = 3). At an initial condition (*vs* = 500 RPM, *ts* = 15 s), the thicknesses of each layer are: HS (underlying substrate (*us*) = glass substrate) 32.1 ± 0.4 µm; AS (*us* = HS) 28.1 ± 0.4 µm; LS (*us* = AS) 49.9 ± 3.9 µm; CS (*us* = LS) 40.9 ± 0.7 µm; and the final HS (*us* = CS) 35.7 ± 0.5 µm. Upon increasing spin speed and time (*vs* = 2000 RPM, *ts* = 120 s), thicknesses were significantly reduced to HS 3.0 ± 0.1 µm, AS 3.9 ± 0.2 µm, LS 19.6 ± 0.4 µm, CS 14.6 ± 0.4 µm, and the final HS 6.9 ± 0.3 µm. f) Tolerance stack‐up of a 106.1 ± 1.7 µm hydrogel unit assembly with individual layer thicknesses measuring: HS (21.5 ± 1.7 µm), CS (20.7 ± 0.6 µm), LS (24.1 ± 1.2 µm), AS (19.4 ± 1.4 µm), and final HS (20.4 ± 0.4 µm) (n = 3 samples). g) Benchmark comparison of the unit cell thickness and volume of our 106.1 ± 1.7 µm hydrogel‐based power source with previously reported hydrogel unit assemblies.^[^ ^13^, ^14^, ^15^, ^16^ ^]^ This work achieves the best thickness‐to‐area aspect ratio, critical for geometrically optimizing power sources for electrical performance.

To evaluate the degree of control over individual layer thicknesses in our LbL method, and to determine whether previously polymerized layers absorb the deposited precursor solution, we generated spin curves of each gel type at each sequential step in the fabrication process. We measured the final thickness of each hydrogel layer stacked in the order of HS (on the glass substrate), AS, LS, CS, and HS under varying spin speeds (*vs* = 500, 750, 1000, 2000 RPM) and spin times (*ts* = 15, 30, 60, 120 s). For layers deposited on previously polymerized gels, we determined the top‐layer thickness by measuring the total stack thickness and subtracting the average thickness of the underlying layers from prior measurements using identical conditions (Figure S15, Supporting Information). All thicknesses are reported as the mean ± standard deviation of three independently prepared samples (*n* = 3). Across all types of hydrogels, the layer thickness decreased with increasing spin speed or spin time, consistent with our expectations (**Figure** **4**a–e; Section S5, Tables S2–S6, Supporting Information). We observe a saturation limit in our layer thicknesses when the spin speed exceeded 2000 RPM and spin times exceeded 60 s. Minimum thicknesses varied slightly among hydrogels, likely due to differences in precursor viscosity and substrate wettability, both governed by surface energy.^[^ ^34^, ^66^ ^]^

To quantify the relationship between spin‐coating parameters and layer thickness—and thereby control both individual and total unit thickness—we fitted the data to a power‐law model (Equation 1).^[^ ^67^ ^]^

(1) $$T=a\cdot {\text{RPM}}^{b}$$

Here, *T* is the film thickness, and *a* and *b* are fitted constants. Power‐law equations were defined for each hydrogel layer in the unit assembly (Section S5, Supporting Information), allowing us to identify the spin speeds and times needed to fabricate a hydrogel‐based power source with a thickness comparable to that of an electrocyte in the electric eel, typically ≈100 µm (Table 1).^[^ ^19^ ^]^ Our resulting multilayer hydrogel unit assembly exhibited a total thickness of 106.1 ± 1.7 µm, with individual layers ranging from 19 to 24 µm (Figure 4f; Section S6, Table S7, Figure S17, Supporting Information). Deviations were generally below 10% except for the LS layer, which showed slightly larger errors near its saturated minimum thickness. Additional variability arose from the intrinsic softness and compliance of hydrogel layers (Figure S16, Supporting Information). Furthermore, we determine that the 106.1 ± 1.7 µm units have a Young's modulus of 9.86 ± 2.48 kPa and break around a 407 ± 90 % elongation (Figure S18, Supporting Information).

Compared to previously reported hydrogel‐based power sources,^[^ ^13^, ^14^, ^15^, ^16^, ^68^ ^]^ Our 106.1 µm compact design achieves the lowest thickness‐to‐area aspect ratio (0.62 m^−1^) and a minimized unit volume of 1.8 × 10^−8^ m^3^ (Figure 4g). Among prior studies, only Zhang et al.^[^ ^15^ ^]^ reported a hydrogel‐based power source with a smaller unit volume; however, because their droplet‐based fabrication technique inherently couples thickness and area, the high aspect ratio limits opportunities to modify the device to improve electrical performance geometrically. In contrast, our LbL fabrication strategy enables support‐free, multilayer hydrogel architectures with control over individual layer thickness, allowing systematic optimization of geometry for enhanced performance. We demonstrate this capability by fabricating 150 µm‐thick unit assemblies with either constant individual layer thicknesses (30 µm for each layer) or variable individual layer thicknesses (45|15|30|15|45 µm) (Tables S8 and S9, Figure S19, Supporting Information), and comparing their electrical performance (Section 5).

## 5 Characterizing the Electrical Performance of the Thin Hydrogel Assemblies with PEDOT:PSS Electrodes

**Figure 5:**
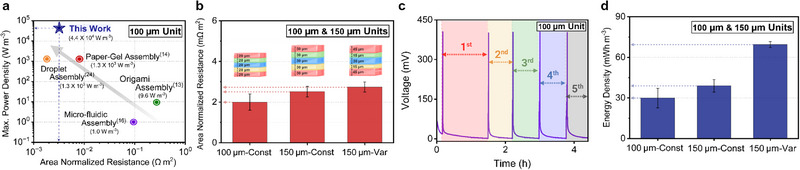
a) Benchmark comparison of area‐normalized internal resistance and instantaneous maximum power density of our thin (106.1 µm) unit assembly with previously reported electric‐eel‐inspired hydrogel systems.^[^ ^13^, ^14^, ^15^, ^16^ ^]^ The power source in this work achieves one of the lowest internal resistances and the highest volumetric power density (44.0 kW m^−3^), enabled by scaffold‐free spin‐coated fabrication. b) Area‐normalized internal resistance of 106.1 and 150 µm unit assemblies (Nyquist plots available in Figure S25, Supporting Information). Two 150 µm assemblies were fabricated with either constant (30 µm) or variable (45|15|30|15|45 µm) layer thicknesses (insets). Both 150 µm unit assemblies exhibited slightly higher resistance than the 106.1 µm unit, yet maintained comparable area‐normalized values (n = 6 samples for each unit). c) Charge–discharge curves of 106.1 µm unit using a constant current density of 6 µA m^−2^ to charge and 0.03 µA m^−2^ to discharge. The ability to recharge our power source is crucial because of spontaneous discharge during fabrication (n = 6). Charge–discharge of the 150 µm units are shown in Figure S28 (Supporting information). d) Energy density of the three unit assembly types calculated from galvanostatic discharge curves (at a current density of 0.03 µA m^−2^) (n = 3). The variable geometry (150 µm‐Var) exhibited significantly higher energy density (69.5 ± 2.2 mWh m^−3^) than both uniform‐layer configurations, confirming that control of the individual layer thickness greatly impacts electrical performance.

Having established control over unit thickness, we next evaluated the electrical performance of our 106.1 µm hydrogel‐based power source against values reported in previous studies^[^ ^13^, ^14^, ^15^, ^16^ ^]^ (**Figure** **5**a, Table 1; calculation details are provided in Section S9.1, Supporting Information). Prior work has most commonly reported open‐circuit potential (OCP), area‐normalized resistance, and instantaneous maximum power density (Table 1). Based on these benchmarks, our thin devices achieved one of the lowest area‐normalized internal resistances at (2.0 ± 0.4) × 10^−3^ Ω m^2^ and the highest instantaneous maximum power density at 44.0 kW m^−3^. Notably, these values are comparable to those of the electric eel, marking a significant step toward functional emulation of this biological system (Figure 5a, Table 1).

In addition to analyzing the resistance of a 106.1 µm unit assembly, we evaluated two 150 µm variants: one with constant individual layer thicknesses and one with variable thicknesses (Figure 5b; Section S9.2, Supporting Information). The 106.1 µm unit exhibited a slightly lower area‐normalized resistance of (2.0 ± 0.4) × 10^−3^ Ω m^2^, a 35% decrease, compared to the constant‐thickness 150 µm unit at (2.7 ± 0.3) × 10^−3^ Ω m^2^ and variable‐thickness 150 µm unit at (2.5 ± 0.3) × 10^−3^ Ω m^2^. This difference is attributed to the LS layer (24 µm) in the 106.1 µm unit being 37.5% thinner compared to the 150 µm device (33 µm‐thick LS layer), which dominates overall unit resistance (Sections S6 and S7, Figure S7, Supporting Information). Meanwhile, the 150 µm units exhibited similar area‐normalized resistances despite differences in internal layer distributions (Table S10, Supporting Information).

Recently, Tholen et al.^[^ ^50^ ^]^ emphasized that electrochemical characterization provides a more accurate assessment of the hydrogel‐based power source performance than simple electrical readouts. Since energy generation in these systems arise from ion diffusion across concentration gradients, electrochemical measurements are designed to non‐invasively and accurately measure various electrical metrics based on chemical reactions in the system.^[^ ^69^ ^]^ Electrochemical methods are commonly used in battery research,^[^ ^69^ ^]^ enabling direct comparison of performance metrics between conventional and hydrogel power sources.^[^ ^50^ ^]^ Furthermore, electrode composition and design remain critical for ensuring reliable long‐term electrical performance and efficient electrode‐electrolyte interactions. Guided by these considerations, we fabricated poly(3,4‐ethylenedioxythiophene) polystyrene sulfonate (PEDOT:PSS) gel electrodes (see Experimental Section), which offer efficient ionic‐to‐electronic conversion, stable interfacing with our hydrogel‐based power source, and a fully compliant, flexible, and biocompatible structure compatible with both flexible and rigid electronics.^[^ ^70^, ^71^ ^]^ These electrodes can also be fabricated with spin coating (Figure S20, Supporting Information), enabling seamless integration with our LbL process. We further verified that the PEDOT:PSS electrodes maintained stable cycling, reversible ion intercalation, and robust interfacial adhesion when interfaced with our hydrogel‐based power sources (Figures S21– S23, Supporting Information).

Using the selected PEDOT:PSS electrodes, we characterized the self‐discharge and charge–discharge curves of the hydrogel unit assembly (Figure 5c; Section S9.2, Supporting Information). Consistent with previous reports,^[^ ^13^, ^50^ ^]^ we observe a rapid exponential decay during self‐discharge and discharge, which indicates limited charge selectivity and susceptibility to co‐ion leakage in the CS and AS layers (Section S9, Supporting Information), thereby accelerating the collapse of the concentration gradient (Figure S24, Supporting Information).^[^ ^13^, ^50^ ^]^ A key limitation of the LbL spin coating technique is that self‐discharge begins spontaneously once a half‐cell (HS‐AS‐LS) is assembled, leading to substantial loss of potential and power during fabrication and measurement setup (Figure S24, Supporting Information). Since OCP is independent of layer thickness and instead is a thermodynamic property determined by the relative steepness of the concentration gradient, we measured the OCP by stacking hydrogel disks (18 mm diameter, 1.8 mm thick) immediately before testing to minimize losses between fabrication and measurement (Table S11, Supporting Information).^[^ ^25^ ^]^ Using this approach, we determined an OCP of 193 ± 18 mV, which aligns with other examples of hydrogel‐based power sources.^[^ ^13^, ^14^, ^15^ ^]^

To overcome the inherent loss of electric potential during fabrication, we demonstrated repeatable charge– discharge cycling using a constant current density of 6 µA m^−2^ to charge and 0.03 µA m^−2^ to discharge (Figure 5c; Figures S26 and S28, Supporting Information). From the galvanostatic discharge profiles, we further derived additional performance metrics used for benchmarking energy storage systems, such as average power density, which reflects energy delivery efficiency, and energy density, which captures the total storage capacity (Figure S27, Support Information). We analyze these metrics for hydrogel‐based power source units with total unit thicknesses of 106.1 and 150 µm, fabricated with both constant and variable layer thicknesses. Both the instantaneous maximum and average power density remained comparable across all three designs, despite similar resistances and concentration gradients (Figures S27 and S29, Supporting Information). Notably, the 150 µm device with variable layer thicknesses exhibited an approximate improvement of 180% in energy density compared to the 150 µm device with a constant layer thicknesses, increasing from 30.0 ± 6.0 mWh m^−3^ for the 106.1 µm unit to 39.0 ± 4.6 mWh m^−3^ for the 150 µm device with constant thickness, and up to 69.5 ± 2.2 mWh m^−3^ for the 150 µm device with variable thickness (Figure 5d). This increase in energy density is mostly attributed to thicker HS layers, which augment ionic storage capacity and extend discharge duration.^[^ ^72^ ^]^ These findings confirm that using LbL spin coating to tune the power source geometry can selectively enhance energy storage without compromising power output.

To further demonstrate the scalability and device‐readiness of our LbL spin coating approach, we quantify the discharge durations, average potential, and average power density of our 150 µm with a constant layer thickness at various constant current densities ranging from 50 nA to 23 mA to simulate performance under various external loads (Figures S30–S32, Supporting Information). We observe that as more power is required for a higher constant current, our discharge duration dissipates rapidly as ion transport equilibrates promptly (Figures S30–S32, Supporting Information). Additionally, we demonstrate integration of our device with a common electronic component by serially stacking ten 100 µm units with PEDOT:PSS electrodes on either end. After assembly and an initial recharging cycle, the scaled power source produced 2V, sufficient to power a red LED (Figure S33, Supporting Information). As expected from the rapid discharge profiles in these systems, the LED was illuminated for only half a second. Nevertheless, this proof‐of‐concept demonstration showcases LbL spin coating as a scalable and practical route for fabricating hydrogel‐based power sources with tunable electrical properties through controlled geometric design.

## 6 Conclusion

In this study, we developed a compliant, biocompatible, and environmentally stable hydrogel‐based power source using a scalable and straightforward LbL spin‐coating technique. This approach enabled the fabrication of the first hydrogel‐based device with a thickness (106.1 µm) and instantaneous power density (44.0 kW m^−3^) comparable to electrocytes of electric fish, marking a key step toward biological emulation in engineered power systems. Incorporation of glycerol into the hydrogel formulations ensured reliable thin‐film fabrication while imparting long‐term hydration stability under ambient conditions and anti‐freezing performance down to −80 °C. Unlike previous systems constrained by external supports, our scaffold‐free LbL process provided precise control over individual layer thickness, enabling systematic geometric optimization to enhance electrical performance. Electrochemical characterization confirmed the stability of cycling and tunability across charge–discharge behavior, energy density, and average power density, establishing a framework for designing hydrogel‐based power sources with tailored outputs.

Integration with PEDOT:PSS hydrogel electrodes further advanced the device toward application‐readiness, yielding fully compliant, ready‐to‐use systems that can be seamlessly interfaced with both flexible and rigid electronics.^[^ ^73^, ^74^, ^75^, ^76^ ^]^ The rapid discharge dynamics, reminiscent of electric double‐layer capacitors (EDLCs), make these systems well suited for high‐power, short‐duration applications when combined with longer‐duration storage modules.^[^ ^74^, ^77^ ^]^ Such hybrid strategies are critical in soft robotics, where bursts of power are needed for rapid actuation, braking, or lifting heavy loads.^[^ ^73^ ^]^ Beyond robotics, these hydrogel‐based power sources show promise for intermittent‐demand sensors such as smart packaging for food and pharmaceuticals,^[^ ^75^ ^]^ and for implantable medical devices requiring pulsed energy delivery, including defibrillators^[^ ^78^ ^]^ and irreversible electroporation (IRE) systems for ablation or drug delivery.^[^ ^79^, ^80^ ^]^

Furthermore, we benchmark our hydrogel power source against both partially polymeric flexible microbatteries, in which one of the active components (anode, cathode, or electrolyte) is polymeric (e.g., aqueous zinc‐ion systems)^[^ ^81^, ^82^ ^]^ and fully polymeric flexible microbatteries^[^ ^83^, ^84^ ^]^ in which all active components are polymeric solutions or gels (Section S10, Supporting Information). In general, fully polymeric devices exhibit electrical performance that is one to two orders of magnitude lower than partially polymeric systems. Still, they offer full biocompatibility and can be fabricated using simpler, more accessible methods. Our power source, in combination with the layer‐by‐layer (LbL) fabrication approach, provides a semi‐automated, scalable route that minimizes overall device dimensions. While our instantaneous power density is comparable to that of partially polymeric microbatteries, our average and energy densities fall within the lower‐median range of reported values for fully polymeric devices.

Further refinements could broaden impact and accelerate translation. Incorporating sacrificial or stimuli‐responsive layers would mitigate premature self‐discharge, enabling more efficient scalability and on‐demand device activation. Improving the ion selectivity of CS and AS layers would reduce leakage, extend discharge duration, boost energy density, and improve cycle life. Addressing these challenges will drive hydrogel‐based power sources beyond laboratory demonstrations toward scalable, application‐ready systems for next‐generation electronics.

## 7 Experimental Section

### Materials and Equipment

All chemicals were purchased from Sigma–Aldrich (Merck‐KGaA), except for carboxylated chitosan (CCS) powder, which was obtained from Aladdin. MilliQ water (18.2 MΩ ·cm) was prepared using a Duo Two‐In‐One water purification system (Avidity Science). Soda lime glass substrates (25 mm × 25 mm × 1.1 mm; Part # CCC3722, Colorado Concept Coatings LLC) were plasma‐cleaned (30 W, PDC‐001, Harrick Plasma) prior to spin coating. Hydrogel precursor solutions were deposited using a spin‐coater (Polos SPIN 200i; WS‐650Mz‐23NPPB, Laurell Technologies). All samples were photopolymerized under a UV lamp (302 nm, 50 W; UVM‐225D, Analytik Jena). Copper plates (EISCO Labs) and PEDOT:PSS hydrogels were used as electrical contacts. Custom 3D‐printed supports and disk‐cell molds were fabricated using a Form 3 printer (Formlabs) and cast with polydimethylsiloxane (PDMS, Dow Sylgard 184).

### Composition of Hydrogel Solutions

All hydrogel precursor solutions were prepared using a binary‐solvent system consisting of water and glycerol with the following formulations:

1) High‐salinity gel: 3.5 M LiCl, 1.72 M acrylamide (AM), 0.021 M N,N′‐methylenebis(acrylamide) (bis), 0.002 M 2‐hydroxy‐4′(2‐hydroxyethoxy)‐2‐methylpropiophenone (Irgacure 2959), and 65% (v/v) glycerol in a binary‐solvent (water and glycerol).
2) Low‐salinity gel: 0.035 M LiCl, 1.72 M AM, 0.021 M bis, 0.002 M Irgacure 2959, 0.01 M carboxylated chitosan (CCS), and 65% (v/v) glycerol in a binary‐solvent.
3) Cation‐selective gel: 1.0 M 2‐acrylamido‐2‐methylpropane sulfonic acid (AMPS), 1.52 M AM, 0.019 M bis, 0.002 M Irgacure 2959, and 70% (v/v) glycerol in a binary‐solvent.
4) Anion‐selective gel: 1.0 M (3‐acrylamidopropyl)trimethylammonium chloride (ATPAC), 1.11 M AM, 0.014 M bis, 0.002 M Irgacure 2959, and 70% (v/v) glycerol in a binary‐solvent.

### Hydration and Dimensional Stability Characterization

Casted disk hydrogel samples (18 mm diameter, 1.8 mm thick) were placed under two conditions: 25 ± 5 °C with 60 ± 5% relative humidity (RH), and 25 ± 5 °C with 11 ± 1% RH. The samples were stored under these conditions for 120 h to evaluate environmental stability. The hydration of each gel was calculated by monitoring the difference in mass at time *t* of each sample using the following equation:

(2) $$\text{Mass ratio}=\frac{m_{t}}{m_{0}}$$

where *m*_0_ and *m*_*t*_ are the initial and instantaneous masses of the hydrogel samples, respectively.

In parallel, the diameter of each sample was measured before and after incubation (0 and 120 h) for both conditions using a digital caliper (NEIKO 01407A, accuracy: ± 0.02 mm) to quantify dehydration‐induced dimensional changes.

### Anti‐Freezing Characterization

The anti‐freezing properties of hydrogel samples were evaluated using differential scanning calorimetry (DSC, TA Q2000). Approximately 5 − 10 mg of each sample was sealed in a standard aluminum pan (Tzero pan and Lid). The samples were cooled from 20 to −80 °C at a rate of 5 °C min^−1^, held at −80 °C for 5 min, and subsequently heated back to 20 °C at the same rate. Heat flow was recorded during the heating process, and the temperature of the endothermic peak was used to determine the freezing point.

### Thickness Characterization of Individual Hydrogel Layers

The thickness of each hydrogel layer was measured using a stylus profilometer (Bruker, model 838‐031‐3) with a 1 mg stylus force, 20 mm scan length, and 25 s duration. Each sample was measured at two positions, and values were averaged over a 10 mm lateral range to ensure consistency across the sample.

Each hydrogel layer was individually measured on a glass substrate. Additionally, for layer‐by‐layer stacking, the individual layer thickness of the newly deposited layer was determined by measuring the total layer thickness of the entire assembly and subtracting the average thicknesses of the layers previously measured (Figure S15, Supporting Information).

### Tensile Measurements of 100 µm Spin Coated Unit

Uniaxial tension measurements of 100 µm spin coated units (n = 3) were measured with MTS Criterion Model 43 with a 50 N load cell (MTS Load Cell LSB.501 D) (Figure S18, Supporting Information). The samples were pulled at a rate of 1 mm s^−1^ until failure.

### Fabrication of Process of Thin‐Layer Hydrogel Units

The procedure for sequential layer‐by‐layer (LbL) spin coating fabrication for a 100 µm unit is described below. This procedure was also used to fabricate the 150 µm units (spin‐coat parameters for these units can be found in Section S7, Supporting information).

Initially, a glass substrate (25 mm × 25 mm) was plasma‐cleaned for 10 min to improve surface wettability. Each hydrogel precursor solution (0.5 mL) was sequentially deposited and spin coated under the following conditions: HS (500 rpm, 30 s), AS (750 rpm, 15 s), LS (2000 rpm, 120 s), CS (1000 rpm, 120 s), and HS (750 rpm, 30 s). After each layer was spun, in situ photopolymerization was performed under a UV light for 60 s exposure at distance of 25 mm to polymerize the layer. The next hydrogel precursor was then deposited, spun, and polymerized. This process was repeated for as many layers were desired.

### Formulation and Fabrication of PEDOT:PSS Hydrogel Electrodes

Initially, (10%) (wt/v) polyvinyl alcohol (PVA, average MW 30 000–70 000) was dissolved in MilliQ water at 88 °C and magnetically stirred at 200 − 300 rpm until the solution was clear. Separately, 5 g of acrylamide (AM) was dissolved in 7 mL of MilliQ water to prepare an AM stock solution. The PVA and AM solutions were magnetically stirred (200 rpm, 25 °C) at a 1:1 ratio until the solution was fully mixed. Afterward 30% (v/v) of poly(3,4‐ethylenedioxythiophene)‐poly(styrenesulfonate) (PEDOT:PSS, 1.1% dispersed in water) and 7.1 mM of bis were added and mixed thoroughly. The resulting PEDOT:PSS hydrogel precursor solution was stored at 2 − 8 °C until use. Before polymerization, the solution was purged with nitrogen for 15 min, followed by the addition of 25% (v/v) glycerol and 0.01 M PI.

It was demonstrated that both the PEDOT:PSS electrodes and subsequent hydrogel layers can be deposited on to each other by spin coating (Figure S18, Supporting Information). However, to ensure the capacity for the PEDOT:PSS electrodes were sufficient for the hydrogel‐based power source, thicker PEDOT:PSS hydrogels layers were required, and chose to cast them for a more efficient fabrication process. To cast PEDOT:PSS electrodes, glass capillary molds were fabricated by clipping two glass slides with 0.5 mm‐thick spacers in between. The PEDOT:PSS hydrogel precursor solution was then introduced into the mold and photopolymerized under UV light (Mineralight UVM‐225D, Analytik Jena, 50 W, 302 nm) at a distance of 15 mm for 10 min. The hydrogel‐based power source was then fabricated through spin coating, removed from the glass slide, and sandwiched between two casted PEDOT:PSS hydrogels for electrical measurements.

### Cyclic Voltammetry of PEDOT:PSS Electrodes

Cyclic voltammetry (CV) measurements were conducted using a three‐electrode configuration. The PEDOT:PSS hydrogel served as the working electrode with a platinum wire acting as a current collector (0.25 mm diameter, Sigma). A standard Ag/AgCl electrode (Fisherbrand, 13‐620‐53) was used as the reference electrode and a platinum wire (1 mm diameter, Sigma) served as the counter electrode. Prior to measurement, the PEDOT:PSS hydrogel was soaked in 3M LiCl solution for 30 min. CV scans were performed from −0.8V to 1V at a scan rate of 0.15 V s^−1^ using a potentiostat (EmStat4S HR, PalmSens). The PEDOT:PSS working electrode and the Ag/AgCl reference electrode were fixed at a center‐to‐center distance of 4.5 cm, resulting in an average ohmic resistance of 43.3 ± 5.7 kΩ (*n* = 3) (Section S8, Supporting Information).

### Electrical Characterization of Hydrogel‐Based Power Source

Electrical measurements of the hydrogel‐based power source were performed using PEDOT:PSS hydrogel electrodes with copper plates as current collectors, except for internal resistance measurements, which were conducted using only copper plates. A light pressure of 10 mg was applied to the copper plates to ensure uniform contact with the hydrogels. Electrical outputs were recorded using a potentiostat (EmStat4S HR, PalmSens) under the following measurement conditions:

1) **Open‐Circuit Potential**: Open‐circuit potentiometry, duration: 1500 s, sampling interval: 3 s
2) **Internal Resistance**: Electrochemical impedance spectroscopy (EIS), frequency range: 0.01 Hz to 3 MHz
3) **Charge–Discharge Cycles**: Mixed mode of chronoamperometry; charging current density: 6 µA m^−2^ with potential cutoff at 0.4 V, discharging current density: 0.03 µA m^−2^ with potential cutoff at 0.001 V

The resistivities (ρ, Ω m) of individual hydrogel layers and the total unit assemblies were calculated by dividing the geometrical dimensions from the measured resistance using EIS (ρ = *R* · *A*/*T*, where *R* is the measured resistance (Ω), *A* the cross‐sectional area (m), and *T* the sample thickness (m)). Resistance was determined by fitting the Nyquist plot to a Randles circuit model in the PSTrace 5.1 software.

### Power and Energy Density Calculations

Instantaneous maximum power density (kW m^−3^) used in Table 1 was calculated based on the maximum power transfer theorem and the measured open‐circuit potential (V) and internal resistance (Ω), as was done in previous work (Section S9.1, Supporting Information). Briefly, the instantaneous maximum power output was calculated as:

(3) $$P_{\max}=\frac{V_{\text{OC,max}}^{2}}{4R_{\text{int}}}$$

The volumetric instantaneous maximum power density (*PD*_max_, W m^−3^) was obtained by dividing *P*_max_ (W) with the total hydrogel unit volume (*V*_Unit_, m^−3^):

(4) $$PD_{\max}=\frac{P_{\max}}{V_{\text{Unit}}}$$

Additional electrical metric such as average power density and energy density were derived from galvanostatic discharge measurements from the charge–discharge curves (Section S9.2, Supporting Information). Average power was calculated by determining the instantaneous power (*P*(*t*) = *I*(*t*) · *V*(*t*)) at each point along the discharge profile by multiplying the applied constant current by the measured voltage and then averaging over all points along the discharge curve. The total energy output was obtained by integrating the instantaneous power *P*(*t*) over the discharge period. Both quantities were normalized with total unit volume (Section S9.2, Supporting Information).

## Conflict of Interest

The authors declare no conflict of interest.

## Supporting information

Supporting Information

## Data Availability

The data that support the findings of this study are available from the corresponding author upon reasonable request.
