# Supplementary material for: Electric‐Fish‐Inspired Thin Hydrogel Electrocytes Achieve High Power Density and Environmental Robustness
Source: Adv Sci (Weinh). 2025 Dec 7;13(9):e19348. doi: 10.1002/advs.202519348 (PMC12904037; doi:10.1002/advs.202519348)
Supplement: Supplementary file 1 — Supporting Information [file ADVS-13-e19348-s001.pdf]

ADVANCED SCIENCE

Supporting Information

Electric-Fish-Inspired Thin Hydrogel Electrocytes Achieve High Power Density and Environmental Robustness

Dor Tillinger§, Wonbae Lee §, Haley M. Tholen,
Derek M. Hall, Joseph S. Najem*

Section S1. Fabrication Process of Unit Layer Assembly

Section S2. Determining Final Material Composition

Section S3. Key Features of the Final Material Composition

Section S4. Procedure for Measuring Individual Layer Thickness

Section S5. Experimental Spin Curve Data for Figure 4a-e

Section S6. Spin Coating Parameters, Fabrication, and Mechanical Characterization of a 100 µm Unit Assembly

Section S7. Spin Coating Parameters and Fabrication of 150 µm Unit Assemblies (Constant and Variable Layer Thicknesses)

Section S8. PEDOT:PSS Hydrogel Electrode Fabrication and Characterization

Section S9. Electrical Characterization

Section S10. Comparison of this work to current flexible microbatteries

References

Section S1. Fabrication Process of Unit Layer-Assembly

1. 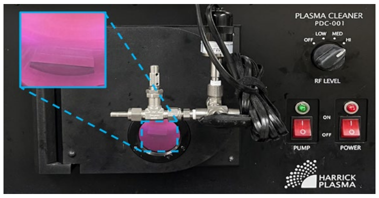

Plasma cleaning glass substrate (25×25 mm^2) for 10 min.

2. 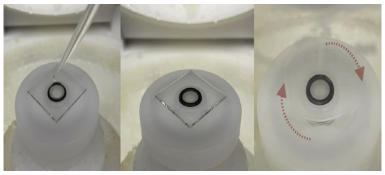
Dispense high-salinity (**HS**) hydrogel precursor solution (0.5 mL) on the entire area of the glass substrate and spin coat.

3. 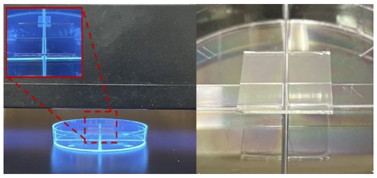
Right after spin coating is complete, cure with UV light source (50-W, 302-nm) 25 mm above the substrate for 60 seconds.

4. 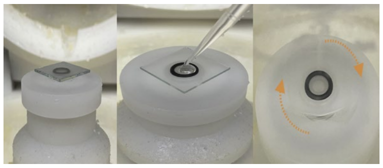
Dispense anion-selective (**AS**) hydrogel precursor solution 0.5 mL on top of underlying cured gel layer and spin coat.

5. 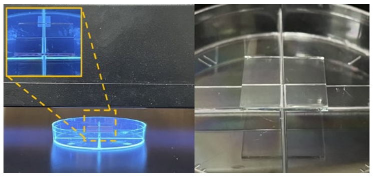
Right after coating, cure with UV light source (50-W, 302-nm) 25 mm above the surface for 60 seconds.

6. 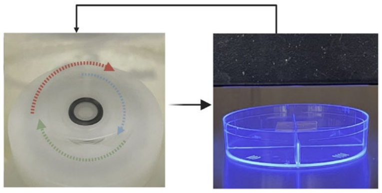
Repeat steps 4~5 for each additional hydrogel precursor solution (low-salinity (**LS**), cation-selective (**CS**), and high-salinity (HS) hydrogel precursor)

Figure S1. Fabrication process of thin-layer unit assembly.

Section S2. Determining Final Material Composition

S2.1 | Introduction

We add glycerol to our final hydrogel formulation as a binary solvent (Experimental Method) mainly to address two main challenges with layer-by-layer (LbL) spin coating: low viscosity and quick dehydration. We selected glycerol over other methods like increasing polymer concentration because glycerol boosts solution viscosity, enhances long-term water retention, and offers anti-freezing properties. **[1–3].** These features enable stable fabrication and dependable performance of hydrogel-based power sources across various environmental conditions. However, adding glycerol increases the resistivity of our hydrogels by reducing ion mobility and lowering the open-circuit potential (OCP), thereby impacting the electrical performance of the power source **[1,4].** To ensure we maximize the electrical output of our material composition, we systematically identified the minimum amount of glycerol required to balance fabrication, environmental stability, and electrical output. This involved (1) tuning glycerol content to optimize fabrication and stability while minimizing its adverse impact on resistivity, and (2) modifying the material composition further to recover and enhance electrical performance.

S2.2 | Optimization of Glycerol Content

To determine the minimum glycerol content needed to enhance the fabrication and stability of hydrogel-based power sources, we first examined environmental stability, as it is a key factor in overcoming dehydration issues during spin coating and electrical measurements. We then verified the feasibility of fabrication using our glycerol content to ensure the viscosity could produce uniform thin layers. Finally, we studied how glycerol content affected electrical performance. We tested each condition independently and selected the final glycerol content based on the combined results of all experiments.

S2.2.1 | Environmental stability

To reduce variability, uneven surface uniformity, and rapid dehydration, we evaluated the water retention and anti-freezing capabilities of our hydrogels by casting hydrogel disks (18 mm diameter, 1.8 mm thick).

To determine suitable hydration, samples with varying glycerol content (0, 20, 40, and 60% (v/v)) were placed in a custom-made humidity chamber at 60% RH (relative humidity), 25 ± 5 °C for 120 h. Based on our observations, hydrogels containing ≥ 60% (v/v) glycerol retained more than 90% of their initial mass, while those below this threshold showed rapid dehydration within the first 24–48 h (**Figure S2**).

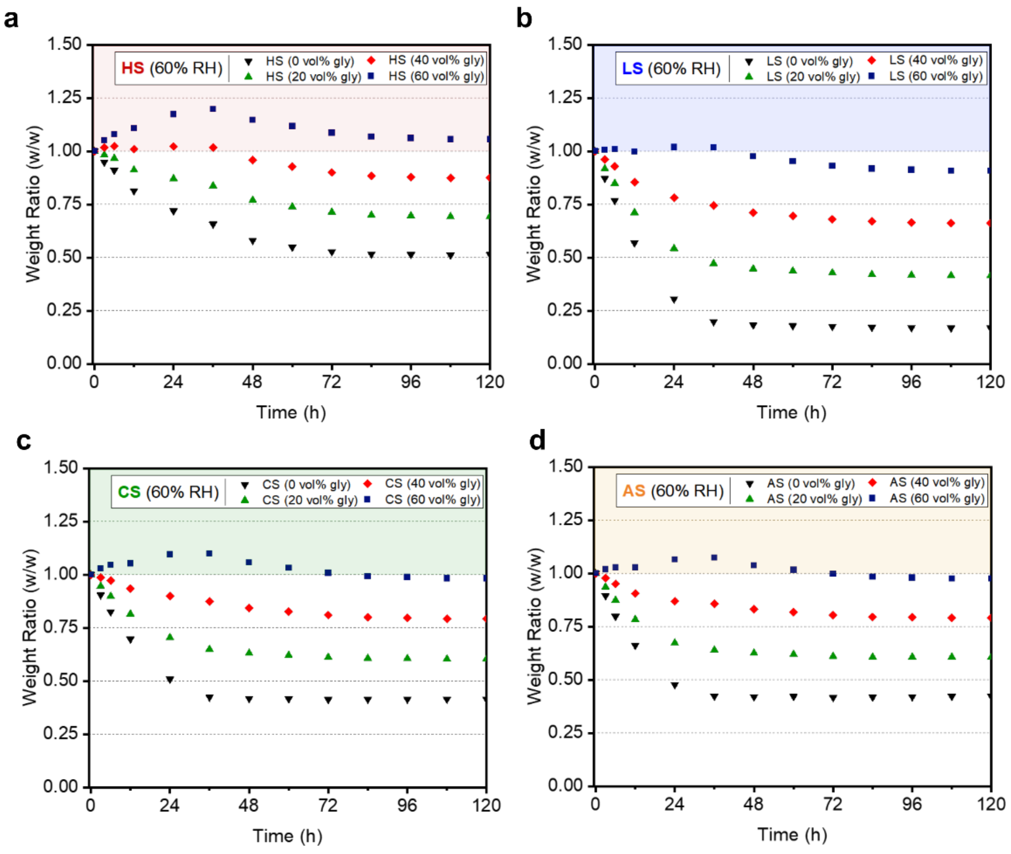

Figure S2. Hydration behavior of individual hydrogel types—(a) HS, (b) LS, (c) CS, and (d) AS—measured over 5 days at 60% RH and 25 ± 5 °C, as a function of glycerol concentration (0, 20, 40, and 60% (v/v)) in the binary solvent system. Each data point represents the normalized mass ratio (m/m_0), where m_0 is the initial gel mass. Hydrogels with 60% (v/v) glycerol retained 95–98% of their initial mass after 5 days across all layer types. In contrast, samples with 40 % (v/v) glycerol showed moderate mass loss, retaining 70–82%, while 20% (v/v) and 0% (v/v) glycerol conditions resulted in significant dehydration, retaining only 45–65% and 30–50%, respectively. The data confirm that at least 60 % (v/v) glycerol is required to effectively suppress water loss and ensure long-term hydration under ambient RH conditions, regardless of hydrogel composition.

In addition, we determined the minimum glycerol content required for anti-freezing capability by visually observing water crystallization of the hydrogels after 12 hours of exposure at –30 °C. Only samples with ≥ 60% (v/v) glycerol-maintained transparency, whereas those with lower glycerol content became visually opaque and cracked due to crystallinity of the water (**Figure S3**).

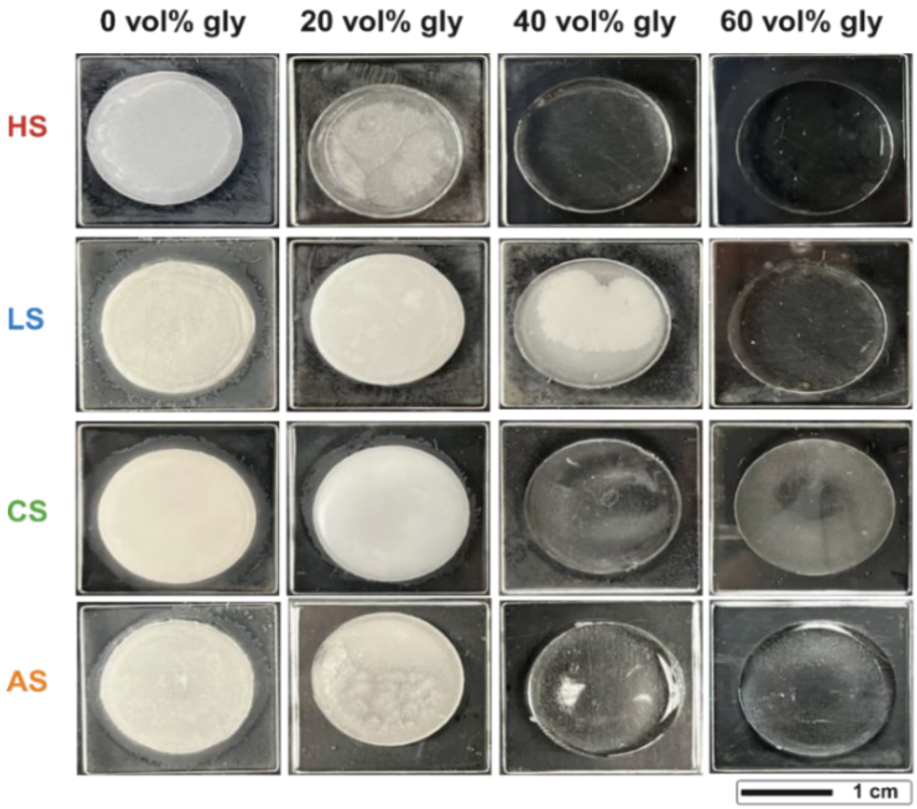

Figure S3. Optical images of disk-type hydrogel samples—HS, LS, CS, and AS—after being exposed to −30 °C for 12 hours, at varying glycerol concentrations (0, 20, 40, and 60% (v/v)). Hydrogels containing 0–40% (v/v) glycerol exhibited partial or complete opacity, internal cracking, and visible ice crystal formation, indicating phase separation and poor freeze tolerance. In contrast, gels with 60% (v/v) glycerol retained optical clarity and structural integrity across all compositions, with no signs of freezing or physical damage. This visual evidence confirms that incorporating ≥ 60% (v/v) glycerol is essential for imparting effective anti-freezing capability under sub-zero conditions.

Based on our evaluations, our hydrogels need at least 60% (v/v) glycerol to ensure environmental stability. We also used this value as a starting point for further optimization of fabrication.

S2.2.2 | Spin Coating Compatibility

We evaluated the minimum glycerol content needed to increase the viscosity of our precursor hydrogel solution for successful uniform LbL spin coating assembly. Our approach involved two steps: (1) spin-coating individual hydrogel layers on glass substrates at various glycerol concentrations; (2) fabricating multilayer structures using the same spin-coating parameters (500 rpm for 50 s). For all hydrogel types spun on glass, 60% (v/v) glycerol was the minimum volume at which all gel types showed surface uniformity, consistent with the environmental stability analysis above (**Figure S4**). However, for multilayer structures, smooth interfacial layering was only achieved starting at 65% (v/v) glycerol (**Figure S5**). Below

this threshold, poor spreading and incomplete coverage occurred, likely due to insufficient viscosity in some hydrogel precursors, premature dehydration, or unfavorable interfacial energy between the solution and substrate. Based on these findings, 65 vol% glycerol was initially chosen for all hydrogel types in the LbL spin coating process. During assessment of fabrication reproducibility, only 57% of samples formed uniform, complete assemblies. To achieve 100% reproducibility, the glycerol content in the selective layers (CS and AS) was increased to 70% (v/v), while maintaining 65% (v/v) glycerol in the salinity layers (HS and LS) (**Figure S6**). We opted to increase glycerol in the selective layers rather than the salinity layers, as the selective layers have minimal impact on the overall resistance of the hydrogel-based power source, raising the overall unit resistivity from 19.13 to 19.57 Ω·m (**Figure S6**) (discussed in more detail in **Section S2.2.4)**.

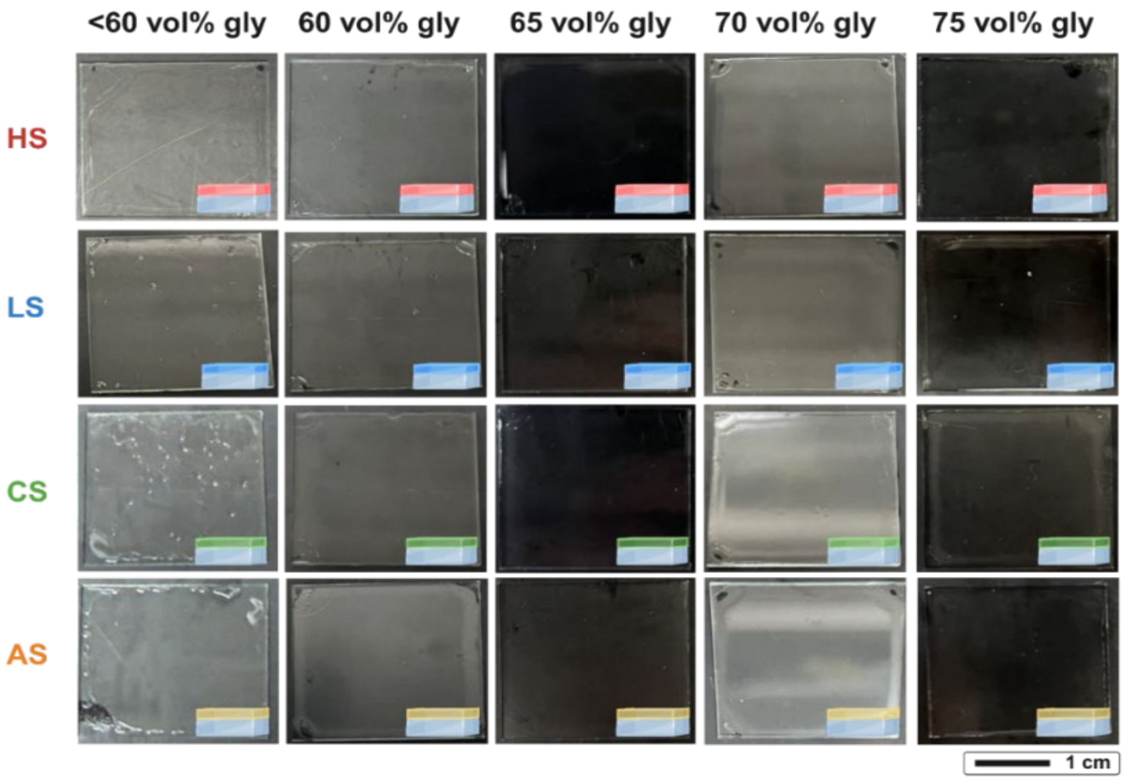

Figure S4. Optical images of spin-coated hydrogel layers—HS, LS, CS, AS—on glass substrates at various glycerol concentrations (< 60, 60, 65, 70, and 75% (v/v)). Each row represents a different hydrogel composition, and each column corresponds to a specific glycerol content in the binary solvent system (water/glycerol). Hydrogels containing 60% (v/v) or more glycerol exhibited smooth, continuous, and uniform film coverage across all layer types. Below 60% (v/v), incomplete spreading and surface defects were observed on all gel types except HS most likely due to low viscosity and premature dehydration during spin-coating. The results indicate that 60% (v/v) glycerol is the threshold for achieving uniform single-layer spin-coated hydrogel films across all compositions.

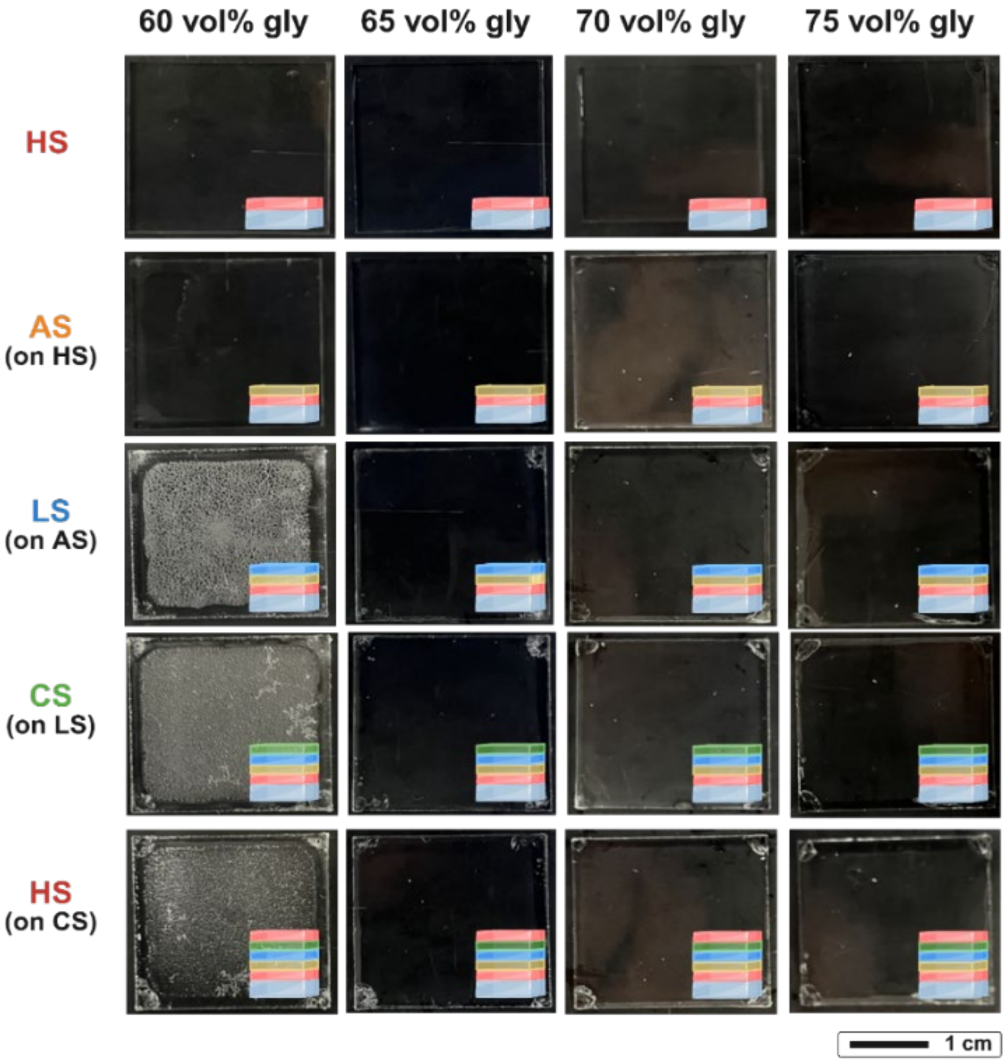

Figure S5. Optical images of sequentially spin-coated hydrogel layers at varying glycerol concentrations (60–75% (v/v)). The layer stacking order follows the configuration: HS → AS → LS → CS → HS, with each layer coated atop the previous one. At ≥ 65% (v/v) glycerol, all hydrogel types formed smooth and continuous interlayer interfaces, resulting in uniform multilayer assemblies. In contrast, at 60% (v/v), significant surface roughness and incomplete coverage were observed, especially at early stacking stages such as LS on AS and CS on LS. These interfacial defects are attributed to low viscosity of the selective hydrogels and premature dehydration, which disrupt uniform film formation during sequential spin-coating. These results indicate that a minimum of 65% (v/v) glycerol is required to achieve reliable and reproducible stacking of multiple hydrogel layers without compromising interfacial integrity.

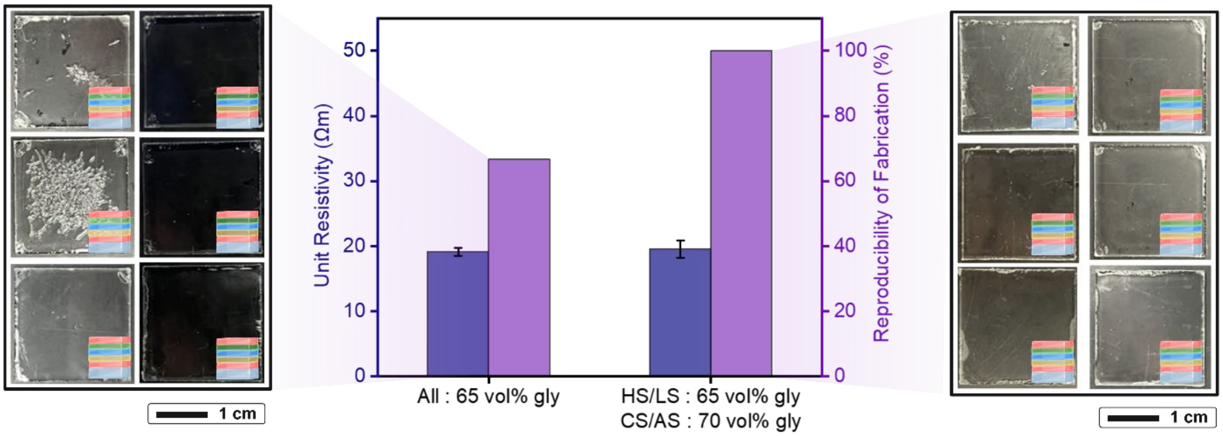

Figure S6. Optimization of fabrication reproducibility in spin-coated thin-layer hydrogel assemblies. Thin-layer-based hydrogel units (HS → AS → LS → CS → HS) were fabricated using a LbL spin-coating process and evaluated for electrical resistivity (left) and reproducibility of successful fabrication (right). The unit resistivity was measured using electrochemical impedance spectroscopy (EIS), while fabrication reproducibility was determined by the percentage of samples (n = 6) forming complete and uniform multilayer stacks. Glycerol content was fixed at 65% (v/v) for HS and LS layers and varied between 65% (v/v) and 70 % (v/v) in the selective layers (CS and AS). Increasing glycerol content in the selective layers to 70% (v/v) significantly improved reproducibility from 57 to 100%, with a negligible increase in resistivity (19.13 → 19.57 Ω·m). These results demonstrate that selective glycerol optimization enhances interfacial integrity and stack reliability without substantially compromising electrical performance.

S2.2.3 | Resistivity

Since adding glycerol naturally increases the viscosity of our hydrogel precursors, we expected it to raise the hydrogel's resistivity, which could negatively affect electrical performance. To study how glycerol concentration impacts ionic resistivity, we measured the ionic resistance of individual gel layers and full unit assemblies using electrochemical impedance spectroscopy (EIS). Glycerol concentrations of 60, 65, 70, and 75% (v/v) were tested with disk-shaped samples (18 mm diameter, 1.8 mm thick), chosen because this geometry was easier to handle, reduced variability, and allowed for isolating compositional effects. Resistivity (ρ) was calculated using the equation $\rho = R \cdot A/L$, where R is the EIS-measured resistance, A is the cross-sectional area, and L is the sample thickness (**Figure S7a**). As glycerol content increased, resistivity values rose consistently across all gel types, primarily due to reduced free water content, which hinders ion mobility (**Figure S7b–f**).**[1,4]** Among all layers, the low-salinity (LS) hydrogel exhibited the

highest resistivity, dominating the total resistance of the hydrogel-based power source unit. Consequently, total unit resistivity increased from 19.2 ± 0.6 to 33.9 ± 0.6 Ω·m across the glycerol concentration range (60-75% (v/v)).

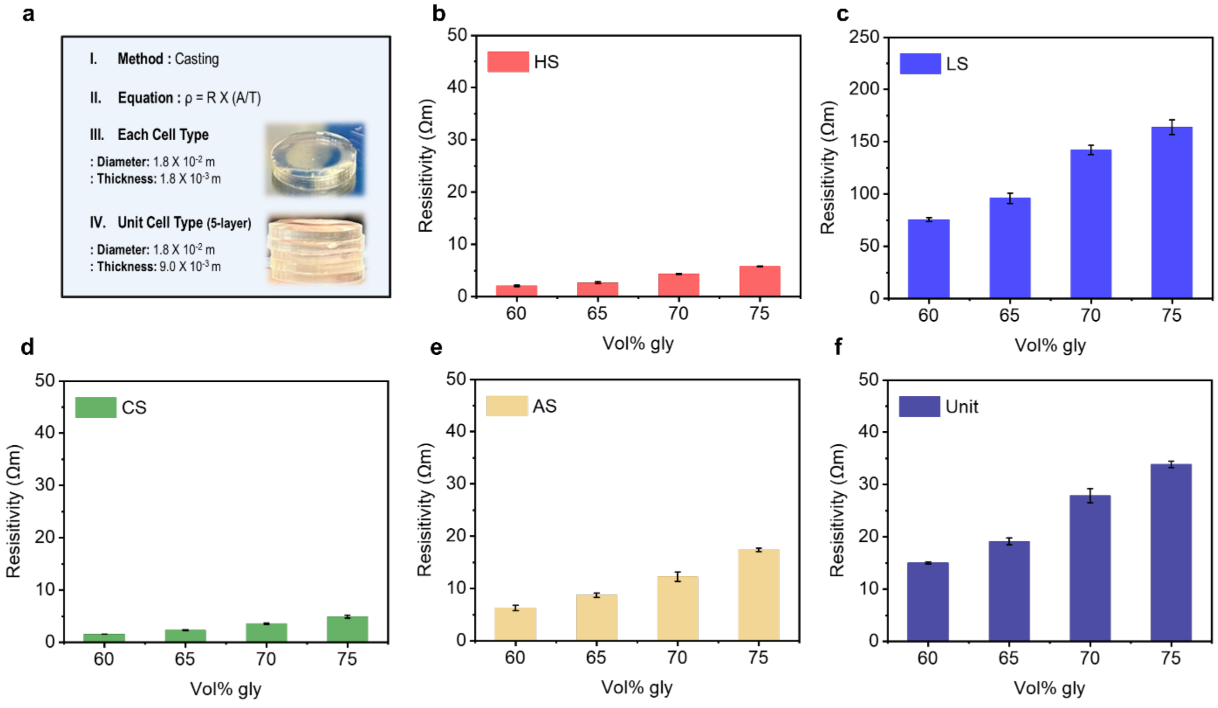

Figure S7. Ionic resistivity of hydrogel samples measured at varying glycerol concentrations (60 – 75% (v/v)) in the binary solvent system (n = 6). (a) Schematic of the casting-based measurement method and sample dimensions. Disk-shaped individual gels had a diameter of 1.8×10^{-2} m and thickness of 1.8×10^{-3} m, while the 5-layer stacked unit cell had a total thickness of 9.0×10^{-3} m. Resistivity (ρ) was calculated using the equation $\rho = R \cdot A/L$, where R is the EIS-measured resistance, A is cross-sectional area, and L is thickness. (b–e) Resistivity trends of each hydrogel type (HS, LS, CS, AS) show a consistent increase with increasing glycerol content due to elevated viscosity and reduced ionic mobility. Among all layers, LS exhibited the highest resistivity across all conditions. (f) The total unit resistivity of the stacked hydrogel assembly also increased with glycerol content, ranging from 19.2 ± 0.6 Ω·m at 60% (v/v) to 33.9 ± 0.6 Ω·m at 75% (v/v).

Since increasing the glycerol content in our hydrogel-based power system impedes electrical output by raising ionic resistivity but is necessary for successful fabrication, we examine the addition of further additives to decrease ionic resistivity while keeping the current glycerol levels (65% (v/v) in the salinity gels and 70% (v/v) in the selective gels).

S2.3 | Maximizing electrical performance by Tuning Solute Composition

In this section, we focus on enhancing electrical performance by reducing ionic resistivity and augmenting open-circuit potential with the selected glycerol volumes for each hydrogel type by examining the effects of (1) incorporating carboxylated chitosan (CCS) into our gels and (2) tuning LiCl concentrations. All tests were conducted using the disk-shaped samples (18 mm diameter, 1.8 mm thick), consistent with prior evaluations.

S2.3.1 Adding Carboxylated chitosan

CCS is a well-known biocompatible, biodegradable, and water-soluble polymer known to enhance ionic conductivity of hydrogels containing both carboxyl and amine groups. [**6**] Since CCS contains carboxyl and amine functional groups with comparable charge densities to those found in our hydrogel compositions, CCS reduces the energy barrier for ion transport, thereby lowering ionic resistivity.[**6**] When incorporating CCS into all hydrogel types, we observe the hydrogel unit (all five hydrogel layers stacked together) ionic resistivity decrease from 19.6 to 18.0 **Ω**·m (a 7.7 % reduction). However, it is accompanied by a noticeable drop in OCP from 185 to 173 mV (a 6.5 % decrease), likely due to the potential protonation of amine groups under acidic conditions, which may disrupt the ion concentration gradient and reduce maximum OCP.**[7]** In contrast, when we selectively incorporate CCS into the LS gel (the most resistive gel type, **Figure S7**) only, the unit ionic resistivity is reduced from 19.6 to 18.2 **Ω**·m. At the same time, the OCP minimally decreased from 185 to 182 mV (**Figure S8a**). This improvement is attributed to the minimized protonation effect, which helps preserve the ion concentration gradient.[7]

S2.3.2 | Adjusting LiCl concentration in Salinity Layers

It is known that for infinitely dilute to semi-dilute concentrations of LiCl, increasing the salt concentration reduces resistivity up to a certain point. [8, 10] We first examined the highest salinity (HS) salt concentration achievable with our optimized binary solvent mixture. We found that our maximum salt concentration in the HS gel is 3.5 M LiCl, and we showed that increasing our concentration of the HS gel from 2.4 M to 3.5 M raised the maximum OCP from 182 to 202 mV with negligible effect on unit resistivity

(**Figure S8b**). Once the maximum salt concentration is determined, we increased the salt concentration in the low-salinity (LS) gel from 0.024 M to 0.035 M while maintaining the HS gel at the maximum salt concentration of 3.5 M (**Figure S8c**). We observe a modest decrease of 5.9% in OCP (202 to 190 mV) as the concentration gradient drops from a 145x difference (LS: 0.024 M, HS: 3.5 M) to a 100x difference (LS: 0.035 M, HS: 3.5 M) but a significant reduction in unit resistivity of approximately 19.3% (18.2 to 14.6 Ω·m) which is desired. One of our main hypotheses is to demonstrate that our spin coating fabrication method significantly enhances the instantaneous maximum power density, based on geometrical optimization, compared to previous works. Therefore, to maintain a more accurate comparison, we choose to maintain a concentration fold of 100x rather than increasing our concentration gradient to 145x. **[8]** If we decide to maintain a 145x concentration gradient, we could have increased our instantaneous maximum power density greater than what we currently report in the manuscript, as our OCP magnitude would be large (refer to SI Section 9 for more details on calculating maximum power density).

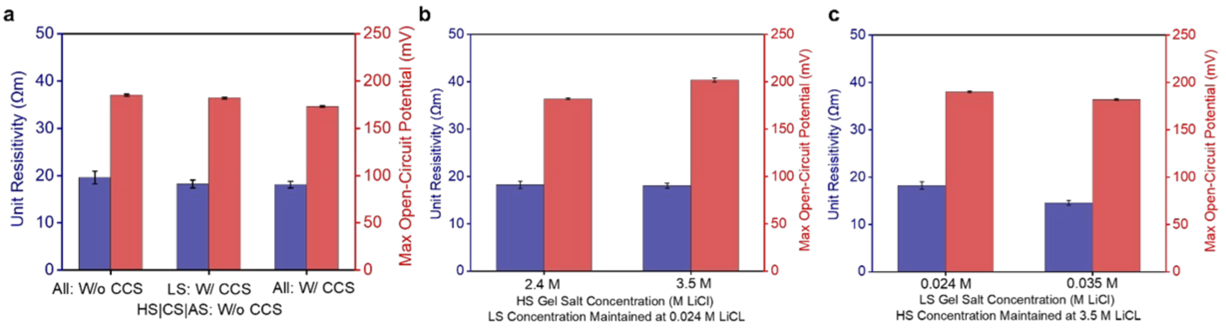

Figure S8. Optimization of thin-layer hydrogel assemblies to simultaneously reduce unit resistivity and maximize open-circuit potential (OCP). All assemblies consisted of five stacked layers in the sequence HS → AS → LS → CS → HS and were tested at room temperature (n = 6). (a) Effect of carboxylated chitosan (CCS) incorporation. Adding CCS to all hydrogel types slightly reduced resistivity (19.6 → 18.0 Ω·m) but caused a marginal OCP drop (185 → 173 mV). In contrast, selective addition of CCS only into the LS hydrogel reduced the unit resistivity to 18.2 Ω·m while maintaining higher OCP (182 mV). (b) Increasing the LiCl concentration in the HS hydrogel from 2.4 M to our maximum possible concentration of 3.5 M, increased the OCP from 182 to 202 mV with minimal change in unit resistivity, indicating improved voltage generation due to higher concentration gradient. (c) Increasing the LiCl concentration in the LS hydrogel from 0.024 M to 0.035 M while maintaining 3.5 M in the HS gel, decrease the unit resistivity significantly by ~19.3% (18.2 → 14.6 Ω·m), while only reducing the OCP by 5.9% (202 → 190 mV).

S2.4 | Conclusion

We identified and modified our material composition by incorporating glycerol to optimize spin coating fabrication and achieve environmental stability in harsh conditions. Additionally, we included carboxylated chitosan (CCS) in the low salinity hydrogel and modified our salt concentrations to enhance our electrical performance, which is hindered by the addition of glycerol. Our final optimized hydrogel composition includes:

(i) Glycerol content: 65 vol% in salinity layers (HS and LS), and 70 vol% in selective layers (CS and AS),

(ii) selective incorporation of carboxylated chitosan (CCS) in the LS hydrogel only,

(iii) And a 100x LiCl concentration gradient in our power source (HS: 3.5 M; LS: 0.035 M).

An additional in-depth comparison between our final hydrogel composition and an unmodified hydrogel composition is provided in Section S3.

Section S3. Key Features of the Final Material Composition

To better demonstrate the benefits of glycerol as an additive regarding fabrication feasibility and environmental stability, we compare our final hydrogel composition containing glycerol (in this section also known as a binary solvent) versus a hydrogel composition without glycerol (henceforth referred to as a single solvent). Table S1 below compares the composition of the single hydrogel composition versus the binary hydrogel composition:

Table S3.1 Comparison of the hydrogel composition of single solvent hydrogel composition and binary solvent hydrogel composition. Single solvent composition is an unoptimized composition, while the binary solvent composition is our optimized composition, discussed more in Section S2.

**Type**	**HS**		**LS**		**CS**		**AS**	
	Single (w/o gly)	Binary (w/ gly)	Single (w/o gly)	Binary (w/ gly)	Single (w/o gly)	Binary (w/ gly)	Single (w/o gly)	Binary (w/ gly)
LiCl (M)	3.5		0.035		-		-	
AMPS (M)	-		-		1.0		-	
APTAC (M)	-		-		-		1.0	
CCS (M)	-		0.01		-		-	
AM (M)	1.72		1.72		1.52		1.11	
Bis (M)	0.021		0.021		0.019		0.014	
PI (M)	0.002		0.002		0.002		0.002	
Glycerol in solvent (vol%)	**0**	**65**	**0**	**65**	**0**	**70**	**0**	**70**

S3.1 | Viscosity of hydrogel precursor solution

As discussed in section 2, glycerol increases the viscosity of the hydrogel composition. This increase in viscosity is essential for successfully fabricating a uniform thin layer using spin coating. **Figure S9** shows that all binary solvents (those with glycerol) had higher viscosity than single solvents (those without glycerol).

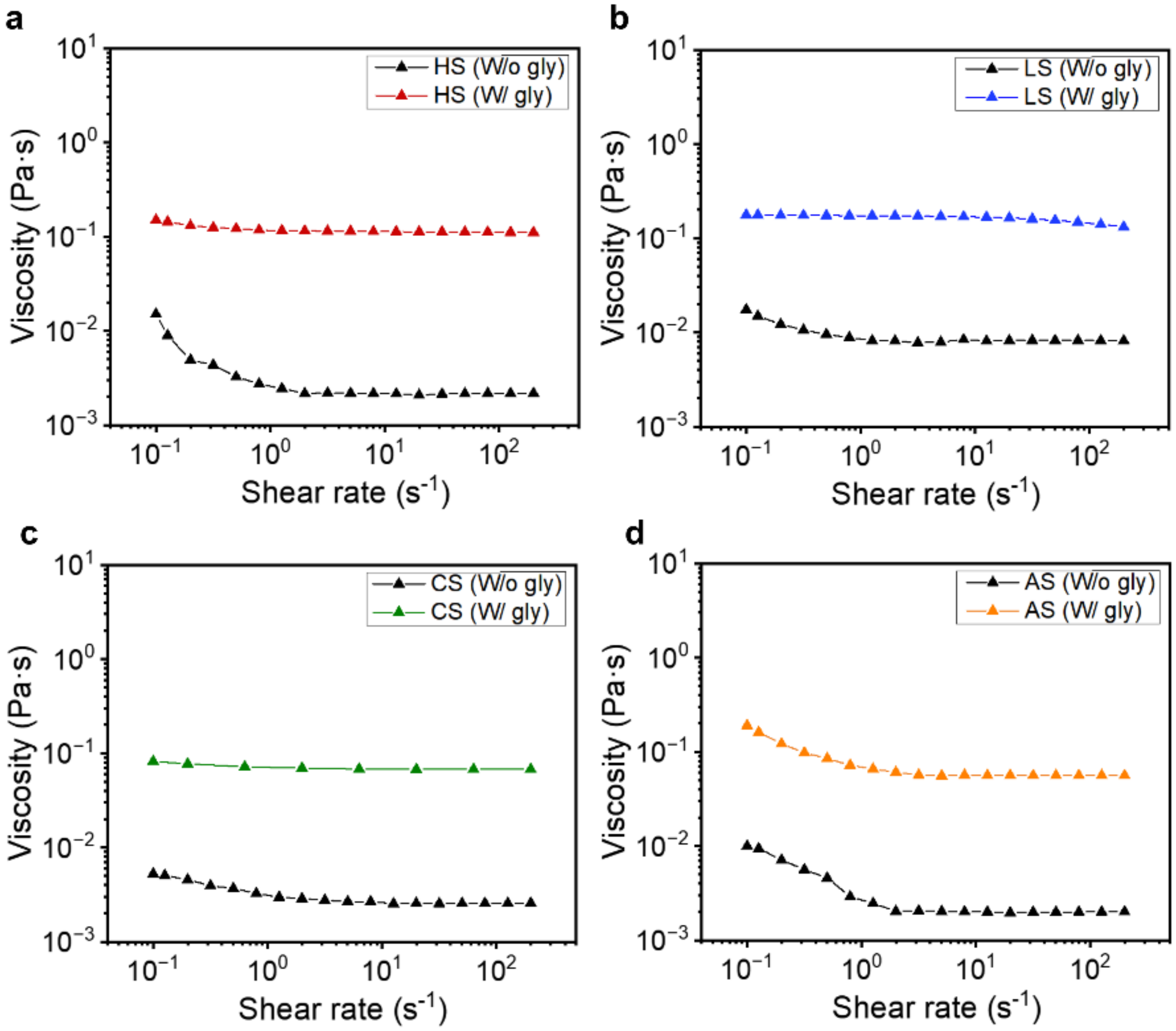

Figure S9. Steady-shear viscosity and Newtonian-plateau viscosities of hydrogel precursor formulations, HS, LS, CS, and AS, prepared with binary-solvent (w/ glycerol) and single-solvent (w/o glycerol) systems. Viscosity was measured over a shear rate range of 0.1–200 s^{-1} at 20 °C using a Kinexus Ultra rheometer (Malvern Instruments). Binary-solvent systems (colored markers) exhibited significantly higher viscosities and broader Newtonian plateaus compared to single-solvent counterparts (black markers). Newtonian viscosities were extracted via sliding-window power-law fitting analysis to identify the plateau region ($|n-1| \approx 0$). Final viscosity values were: 113.0 ± 0.4 mPa·s (HS), 174.5 ± 0.9 mPa·s (LS), 68.2 ± 0.8 mPa·s (CS), and 56.2 ± 0.1 mPa·s (AS) for binary systems, compared to 2.1 ± 0.03, 8.2 ± 0.02, 2.6 ± 0.01, and 2.0 ± 0.02 mPa·s for single-solvent systems, respectively. These results confirm that glycerol incorporation in the binary-solvent system significantly increases the viscosity of hydrogel precursor solutions, thereby enhancing coating stability under fabrication-relevant shear conditions.

S3.2 | Feasibility of fabrication

As demonstrated in Section S3.1 and discussed in Section S2, the addition of glycerol increases viscosity and stabilizes the long-term hydration of our hydrogels. Regarding spin coat fabrication, adding glycerol allows for both a consistent, smooth surface and uniform fabrication, which otherwise is lacking in a single solvent due to low viscosity and rapid dehydration.

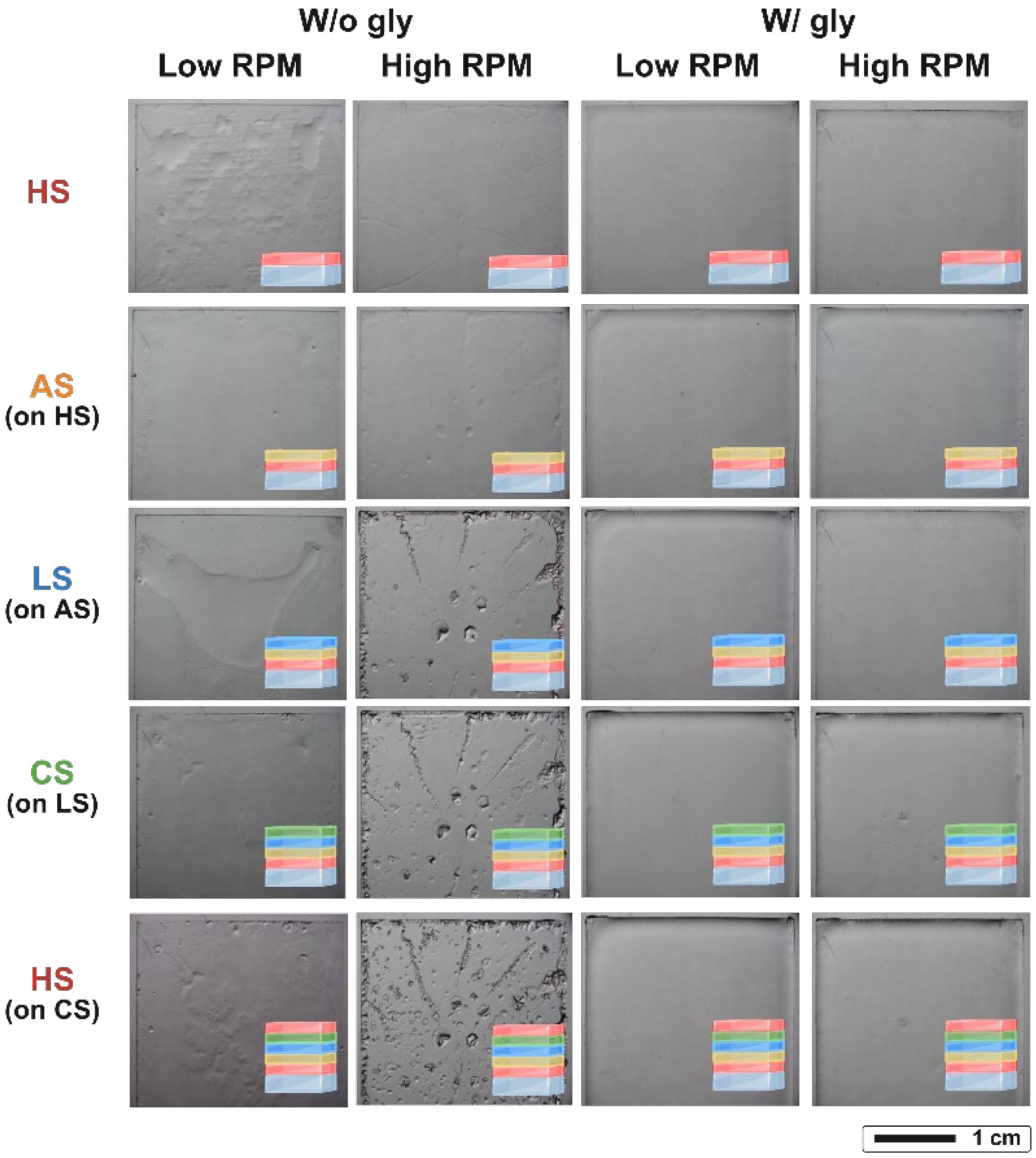

Figure S10. Surface images of stacked LbL spin-coating (Sequence of HS, AS, LS, AS, and HS) under different spin speeds and solvent types. Regardless of RPM, uniform and smooth stacking was consistently achieved when using optimized binary solvent system (w/ glycerol). In contrast, single-solvent system (w/o glycerol) exhibited non-uniform and rough surfaces during fabrication.

S3.3 | Dimensional stability & long-term hydration at harsh environmental conditions

As discussed in section S2, glycerol is hygroscopic and therefore promotes long-term hydration. [1-3] Maintaining long-term hydration is essential for practical electrical operation, as it facilitates ion transport and prevents hydrogel shrinking, which could increase contact resistance between different hydrogel layers. **Figure S11** shows the dimensional stability of hydrogel disks at both 60% and 11% RH, while **Figures 3c-f** in the main text and **Figure S12** depict the overall dehydration of all gel types at these humidity levels. At 60% RH, the binary solvent (with glycerol) gels either retain their initial hydration level or swell over 5 days while preserving their disk shape. Conversely, the single solvent (without glycerol) dehydrates within the first 24 hours and shrinks, reducing contact area and increasing contact resistance when stacking a hydrogel-based power source. Under harsher conditions of 11% RH, where less moisture is available in the environment, both the single and binary solvent gels dehydrate over 5 days, but the binary solvent maintains its dimensional stability during this period.

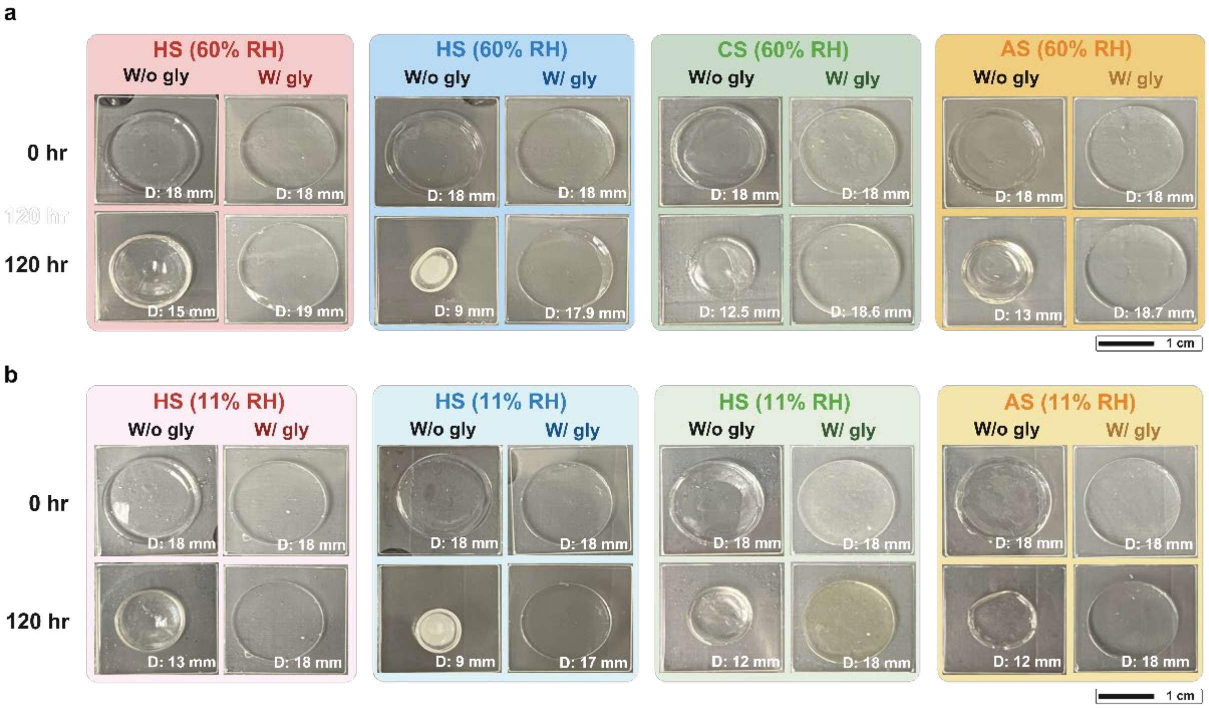

Figure S11. Optical images of disk-shape hydrogels (HS, LS, CS, and AS) prepared with either a single-solvent system (water only) or a binary-solvent system (water and glycerol). Samples were stored for 5 days under (a) 60% RH and (b) 11% RH at 25 ± 5 °C. Diameter values at 0 and 120 h indicate dimensional stability over time. Under 60% RH, single-solvent LS gels showed up to 50.0% shrinkage (18 → 9 mm), while their binary-solvent counterparts maintained dimensional integrity with only 0.6% change. CS and AS gels exhibited 27.8–30.6% shrinkage without glycerol, compared to a slight swelling (–3.3 to –3.9%) with glycerol. Under 11% RH, dimensional loss was even more pronounced in single-solvent gels (up to 50.0%), whereas binary-solvent gels consistently retained ≥ 94% of their original diameter across all gel types. These results confirm that binary-solvent formulations significantly improve dimensional stability under both moderate and severe dehydration conditions

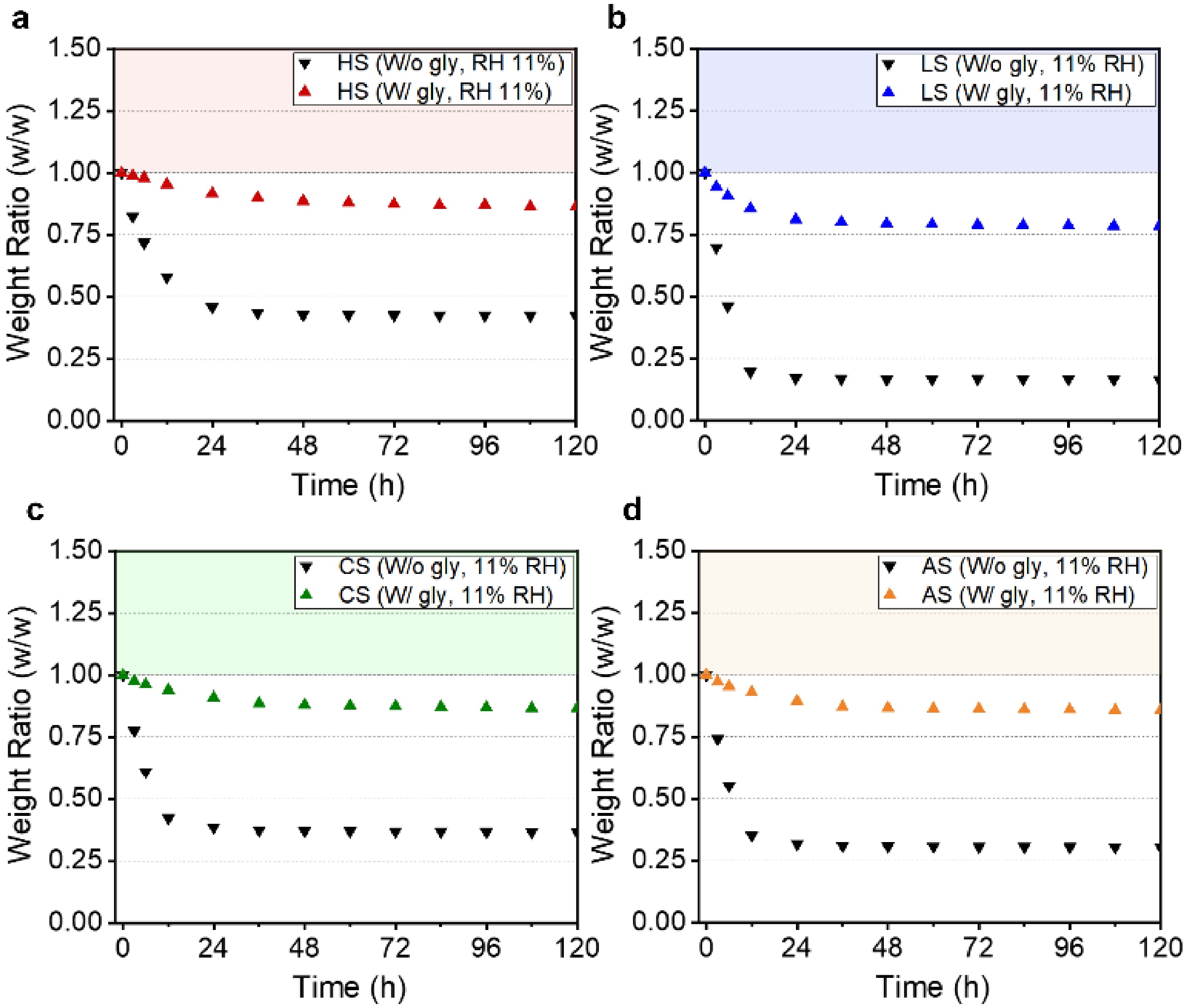

Figure S12. Time-dependent weight ratio (w/w) of four hydrogel types—HS, LS, CS, and AS—over 120 h under controlled conditions (25 ± 5 °C, 11 ± 1% RH). Binary-solvent hydrogels (colored markers) showed significantly higher weight ratios after 120 h: 86.2% (HS), 78.5% (LS), 86.7% (CS), and 85.8% (AS), compared to their single-solvent counterparts (black markers), which retained 42.4%, 16.4%, 36.6%, and 30.4%, respectively.

S3.4 | Anti-freezing Capability

As discussed in Section S2, glycerol offers anti-freezing properties to hydrogel compositions. **Figure S13** shows that all gel types without glycerol crystallize except for high salinity because of the high LiCl concentration, which lowers the freezing point. In contrast, all gel types with glycerol, both as disks and thin layers, did not visibly freeze or crystallize.

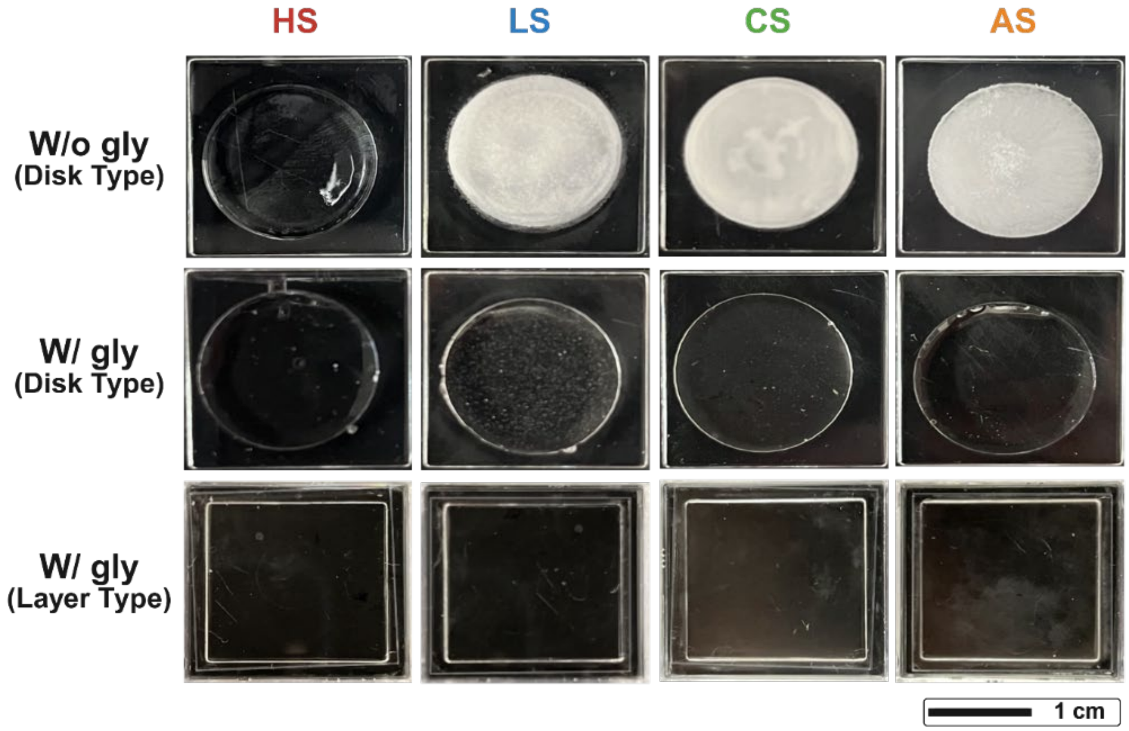

Figure S13. Optical images of hydrogel samples (HS, LS, CS, and AS) prepared using single-solvent (w/o glycerol) and binary-solvent (w/ glycerol) formulations in disk-shape and thin-layer configurations, after storage at –30 °C for 12 h. Note that thin-layer samples could not be fabricated using the single-solvent-based composition due to poor layer formation during spin-coating.

Furthermore, we quantify the ionic resistivity of each gel type and the full unit at sub-zero temperatures. Utilizing the cast disk hydrogels (18 mm in diameter, 1.8 mm in thickness), we stored the gels in the freezer at -25 to 30 °C for 30 hours post polymerization, after which EIS measurements were conducted for each gel in the freezer at -25 °C. We observe that at subzero temperature the ionic resistivity of all gel types and the units increase compared to measurements at room temperature. However, all gel types were still conductive and produced a maximum potential of 162 ± 1 mV (n = 3) at subzero temperature compared to

the 193 mV at room temperature. The increase in ionic resistivity is expected due to slower ionic mobility at subzero temperature and is consistent with literature [17].

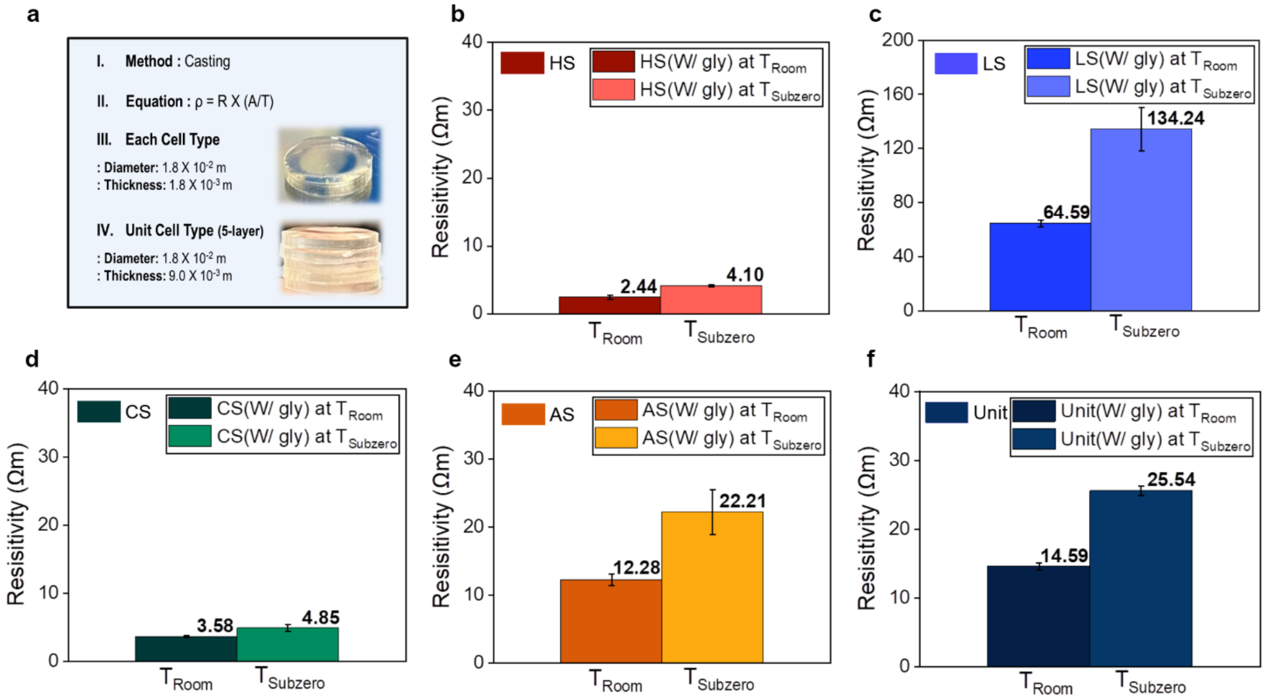

Figure S14. Ionic resistivity of hydrogel samples at subzero temperatures (n = 3). (a) Schematic of the casting-based measurement method and sample dimensions. Disk-shaped individual gels had a diameter of 1.8×10^{-2} m and thickness of 1.8×10^{-3} m, while the 5-layer stacked unit cell had a total thickness of 9.0×10^{-3} m. Resistivity (ρ) was calculated using the equation $\rho = R \cdot A/L$, where R is the EIS-measured resistance, A is cross-sectional area, and L is thickness. (b–e) Ionic resistivity of each hydrogel type (HS, LS, CS, AS) increases at subzero temperature due to reduced ionic mobility. (f) The total unit resistivity of the stacked hydrogel assembly also increased at subzero temperature but the unit is still ionically conductive producing a max open circuit potential of 162 ± 1 mV.

Section S4. Procedure for Measuring Individual Layer Thickness

S4.1 | Protocol for determining individual layer thickness

We measure individual layer thicknesses of multilayer unit assemblies using a stylus profilometer to determine spin curves and control the layer thicknesses of our hydrogel-based power source units (**Figure 4** of the main text). It is essential to measure the thickness of each hydrogel type on the substrate where it is spin-coated, as the underlying substrate significantly influences the thickness and uniformity of the subsequent layer. Therefore, our procedure for determining each layer's thickness in the multilayer assembly starts by measuring the average thickness of the previous hydrogel layer(s). We then measure the total thickness of the multilayer stack and subtract the known average thickness of the last layer. For example, we initially measure the average thickness of the first high-salinity layer at specific spin-coating parameters. Next, we spin-coat the anion-selective layer on top of the initial high-salinity layer and measure the total thickness of the bilayer stack. To find the average thickness of the anion-selective layer, we subtract the total height from the average thickness of the first high-salinity layer. This process is repeated for each layer in the power source stack (**Figure S14**).

1

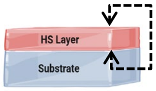

T_{HS} = Total Thickness

2

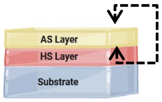

Applying the condition of $T_{HS/Ref}$

T_{AS} = Total Thickness − $T_{HS/Ref}$

3

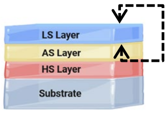

Applying the condition of $T_{HS/Ref}$, and $T_{AS/Ref}$

T_{LS} = Total Thickness − $T_{HS/Ref}$ − $T_{AS/Ref}$

4

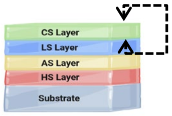

Applying the condition of $T_{HS/Ref}$, $T_{AS/Ref}$, and $T_{LS/Ref}$

T_{CS} = Total Thickness − $T_{HS/Ref}$ − $T_{AS/Ref}$ − $T_{LS/Ref}$

5

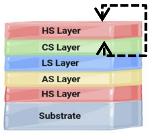

Applying the condition of $T_{HS/Ref}$, $T_{AS/Ref}$, $T_{LS/Ref}$ and $T_{CS/Ref}$

T_{HS} = Total Thickness − $T_{HS/Ref}$ − $T_{AS/Ref}$ − $T_{LS/Ref}$ − $T_{CS/Ref}$

Figure S15. Protocol for determining individual layer thicknesses in spin-curve measurements using reference subtraction method.

S4.2 | Noise from stylus profilometer measurements

When measuring the individual layer thickness of the upper gels in the stack, we observed increased noise in the stylus profilometer data. We attribute the added noise to the displacement of the stylus on the soft hydrogel surface. This limitation remains even when the stylus tip force is minimized (0.5 mg, equivalent to 4.91×10^{-6} N) (**Figure S15**).

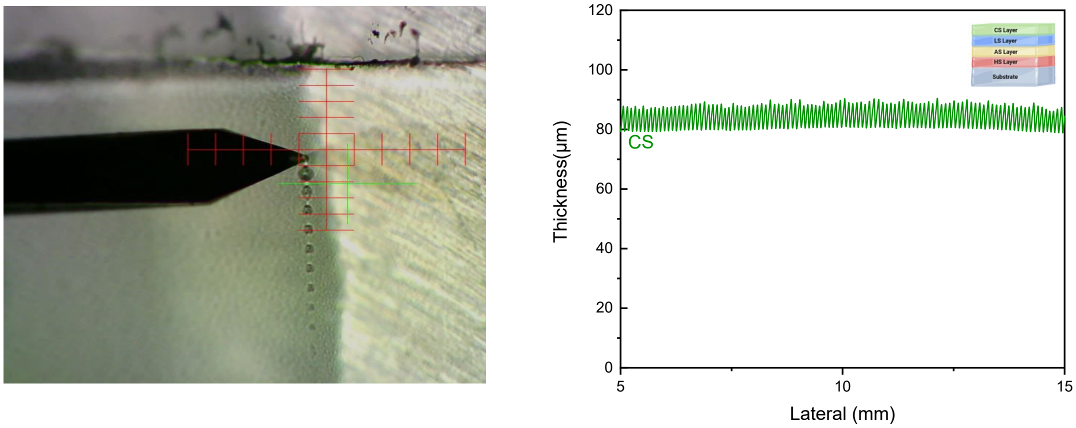

Figure S16. Limitation of stylus profilometer for thickness measurement of soft hydrogel layers. (a) Optical image showing stylus contact on the surface of a spin-coated hydrogel. (b) Thickness profile of the stacked layer (HS–AS–LS–CS) measured over 10 mm. Surface deformation caused by the soft nature of the hydrogel introduces signal noise, even when using a minimized stylus force.

Section S5. Experimental Spin Curve Data for Figure 4a-e

5.1. Average thickness (µm) of HS on glass substrate (n=3)

Spin time (s)	Spin speed (RPM)				Fitted spin-curve equation
	500	750	1000	2000	
15	32.1 ± 0.4	22.1 ± 0.7	16.7 ± 0.4	10.7 ± 0.2	$h = 6378.18 \times RPM^{-0.85}$
30	20.7 ± 0.2	14.9 ± 0.1	12.1 ± 0.0	7.9 ± 0.1	$h = 1871.94 \times RPM^{-0.73}$
60	13.5 ± 0.2	9.3 ± 0.2	7.6 ± 0.2	4.1 ± 0.2	$h = 2777.66 \times RPM^{-0.86}$
120	10.9 ± 0.6	7.9 ± 0.2	6.0 ± 0.3	3.0 ± 0.1	$h = 2672.50 \times RPM^{-0.88}$

1) Unit of thickness is µm

5.2. Average thickness (µm) of AS on HS gel (n=3)

Spin time (s)	Spin speed (RPM)				Fitted spin-curve equation
	500	750	1000	2000	
15	28.1 ± 0.4	19.4 ± 0.9	15.2 ± 0.5	9.8 ± 0.6	$h = 4418.32 \times RPM^{-0.82}$
30	18.9 ± 0.4	13.5 ± 0.4	10.9 ± 0.2	6.8 ± 0.7	$h = 2208.63 \times RPM^{-0.77}$
60	14.8 ± 0.4	10.7 ± 0.2	8.3 ± 0.3	5.1 ± 0.2	$h = 2034.23 \times RPM^{-0.79}$
120	12.1± 0.4	8.7 ± 0.1	7.0 ± 0.3	3.9 ± 0.2	$h = 1738.73 \times RPM^{-0.80}$

1) Unit of thickness is µm
2) Applying fabricating condition of HS reference (1000 RPM/30 s)
3) Average AS thickness is total thickness substrates thickness of HS reference

5.3. Average thickness (µm) of LS on AS gel (n=3)

Spin time (s)	Spin speed (RPM)				Fitted spin-curve equation
	500	750	1000	2000	
15	49.9 ± 3.9	41.0 ± 0.7	35.5 ± 0.7	28.1 ± 1.1	$h = 704.91 \times RPM^{-0.43}$
30	42.6 ± 0.8	33.8 ± 0.5	28.5 ± 0.5	22.7 ± 1.3	$h = 832.05 \times RPM^{-0.48}$
60	36.6 ± 0.5	30.0 ± 0.4	26.3 ± 0.8	20.9 ± 0.5	$h = 485.37 \times RPM^{-0.42}$
120	32.2 ± 1.2	28.2 ± 0.4	25.2 ± 0.5	19.6 ± 0.4	$h = 356.18 \times RPM^{-0.38}$

1) Unit of thickness is µm
2) Applying fabricating condition of HS reference (1000 RPM /30s), and AS reference (1000 RPM/30 s)
3) Average LS thickness is total thickness substrates thickness of HS and AS reference

5.4. Average thickness (µm) of CS on LS gel (n=3)

Spin time (s)	Spin speed (RPM)				Fitted spin-curve equation
	500	750	1000	2000	
15	40.9 ± 0.7	31.9 ± 0.7	28.7 ± 0.4	21.9 ± 0.9	$h = 720.58 \times RPM^{-0.46}$
30	34.6 ± 0.5	27.5 ± 0.6	22.3 ± 0.5	17.6 ± 0.8	$h = 867.14 \times RPM^{-0.52}$
60	28.5 ± 0.8	25.3 ± 0.7	21.5 ± 1.0	16.1 ± 0.7	$h = 376.06 \times RPM^{-0.41}$
120	27.5 ± 0.5	22.5 ± 0.9	19.4 ± 0.4	14.6 ± 0.4	$h = 480.57 \times RPM^{-0.46}$

1) Unit of thickness is µm
2) Applying fabricating condition of HS reference (1000 RPM /30s), AS reference (1000 RPM/30 s), and LS reference (2000 RPM/120 s)
3) Average CS thickness is total thickness substrates thickness of HS, AS, and LS reference

5.5. Average thickness (µm) of HS on CS gel (n=3)

Spin time (s)	Spin speed (RPM)				Fitted spin-curve equation
	500	750	1000	2000	
15	35.7 ± 0.5	26.0 ± 0.9	21.2 ± 0.5	15.3 ± 0.4	$h = 2081.56 \times RPM^{-0.66}$
30	27.1 ± 0.7	20.0 ± 0.9	15.7 ± 0.6	10.6 ± 0.5	$h = 2278.91 \times RPM^{-0.71}$
60	20.2 ± 0.4	14.7 ± 0.5	11.3 ± 0.6	7.7 ± 0.7	$h = 2057.50 \times RPM^{-0.75}$
120	18.8 ± 0.5	14.0 ± 0.5	11.0 ± 0.4	6.9 ± 0.3	$h = 1811.68 \times RPM^{-0.74}$

1) Unit of thickness is µm
2) Applying fabricating condition of HS reference (1000 RPM /30s), AS reference (1000 RPM/30 s), LS reference (2000 RPM/120 s), and CS reference (1000 RPM /120 s)
3) Average HS thickness is total thickness substrates thickness of HS, AS, LS, and CS reference

Section S6. Spin Coating Parameters, Fabrication, and Mechanical Characterization of a 100 µm Unit Assembly

6.1. Fabrication condition and layer thickness of 100 µm unit-assembly (n=3)

Type	Fabrication Condition		Thickness (µm)
	Spin speed (RPM)	Spin time (s)	
HS	500	30	20.4±0.4
AS	750	15	19.4±1.4
LS	2000	120	24.1±1.2
CS	1000	120	20.7±0.6
HS	750	30	21.5±1.7
Unit-Assembly	-	-	106.1± 1.66

1) Roughness is surface height profile from the mean line over a given evaluation length and provides a statistical measure of surface variations
2) Roughness is the actual measured lateral range from 5 to 15mm.
3) Sample number is 6.

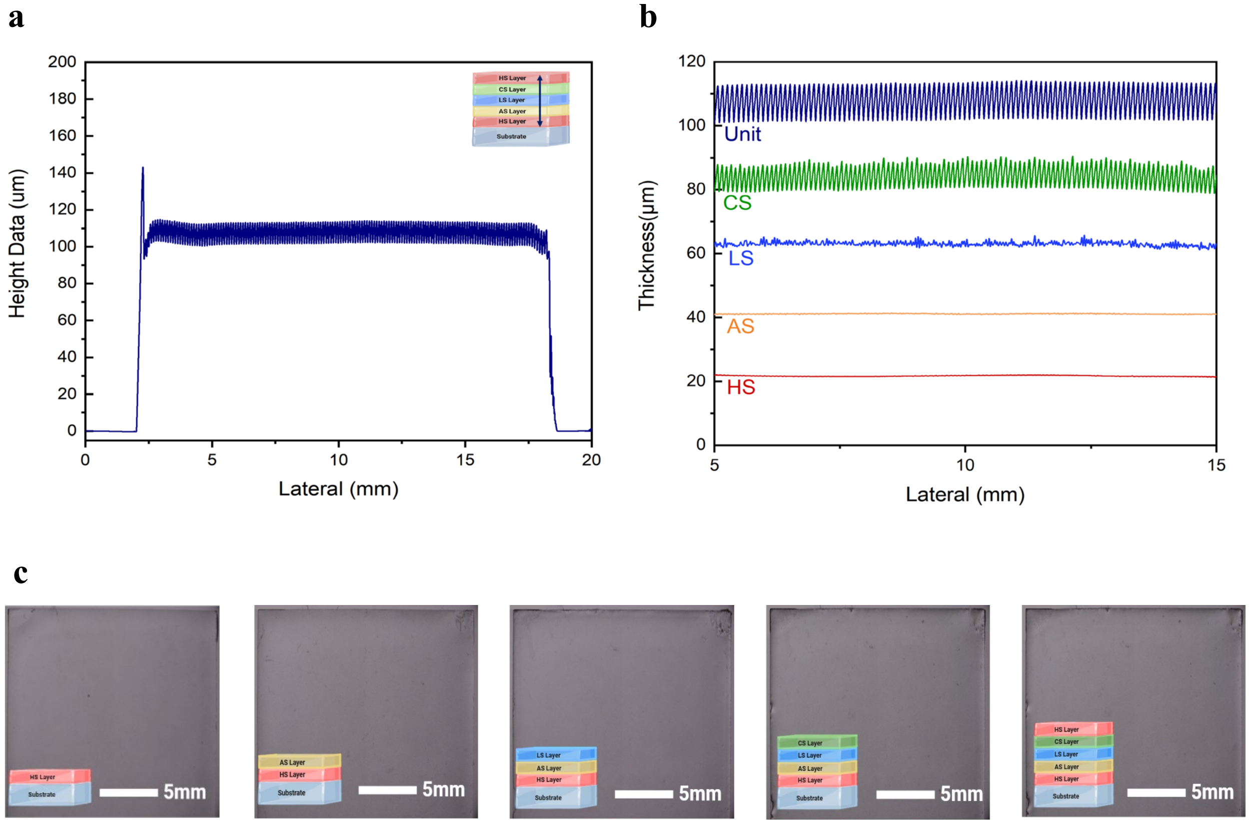

Figure S17. Profilometer data of (a) unit assembly and (b) each layer. (c)Surface image of stacking layers of uniform 100µm thin layer-assembly.

To demonstrate the flexibility and compliance of our hydrogel power source, we performed tensile tests on three 100 μm unit devices. From the stress-strain curves **(Figure S18)**, we calculated the Young's modulus of the gels to be 9.86 ± 2.48 kPa and the percent elongation at break to be 407 ± 90%. Compared to other hydrogels, which usually have a Young's modulus in the range of 10^0 to 10^2 kPa [18], our gels tend to be on the lower end. However, the mechanical strength of hydrogels varies greatly depending on polymer type, solvent, concentration, and crosslinking density [20].

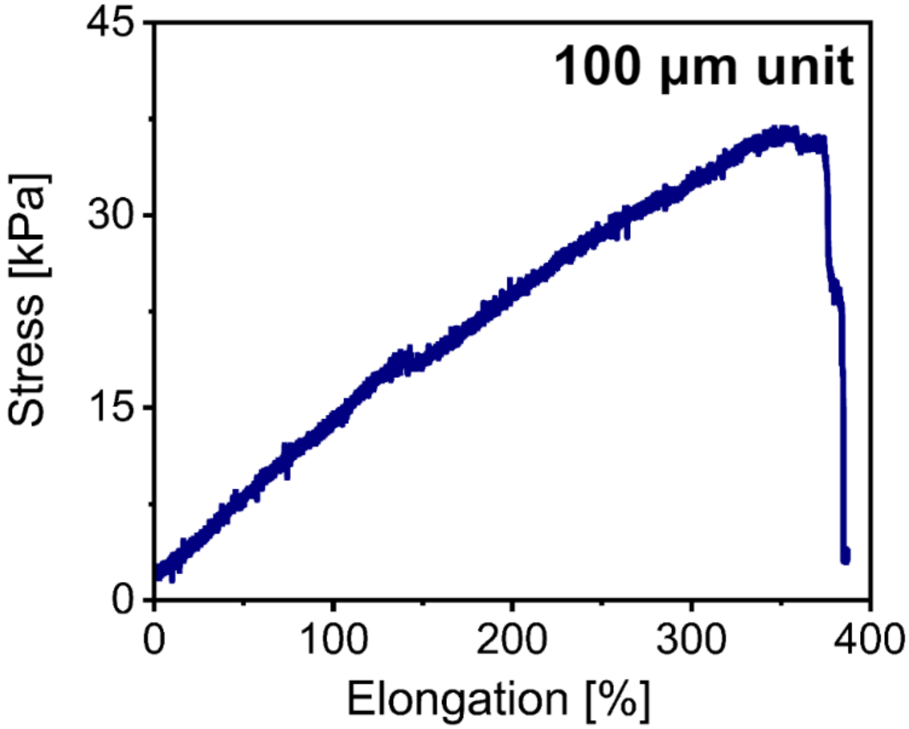

Figure S18. Stress-strain curve of a single 100 μm thick hydrogel power source.

Section S7. Spin coating Parameters and Fabrication of 150 μm Unit Assemblies (Constant and Variable Layer Thicknesses)

7.1. Fabrication condition and layer thickness of 150 μm variable unit-assembly (n=3)

homogeneous Type	Fabrication Condition		Thickness			Roughness (μm)
	Spin speed (RPM)	Spin time (s)	Expected (μm)	Actual (μm)	Error (%)	
HS	310	15	45	45.4 ± 1.0	3.8	3.47± 1.50
AS	880	15	15	14.9 ± 0.8	6.7	2.07± 0.30
LS	1000	60	30	33.2 ± 0.4	23.0	1.61± 0.78
CS	1750	120	15	13.8 ± 1.2	0	5.11± 2.17
HS	400	15	45	44.3 ± 2.7	2.0	5.10± 0.49
Unit-Assembly	-	-	150	151.5± 2.7	1.0	5.10± 0.49

1) Expected (Value) is the thickness based on spin curve section, and Actual (Value) is profilometer data.
2) Error ($= \frac{|Actual - Expected|}{Expected} \times 100$) is calculated based on average value
3) Roughness is surface height profile from the mean line over a given evaluation length and provides a statistical measure of surface variations
4) Roughness is the actual measured lateral range from 5 to 15mm.
5) Sample number is 3.

7.2. Fabrication condition and layer thickness of 150 μm constant unit-assembly (n=3)

homogeneous Type	Fabrication Condition		Thickness			Roughness (μm)
	Spin speed (RPM)	Spin time (s)	Expected (μm)	Actual (μm)	Error (%)	
HS	510	15	30	27.5 ± 1.4	8.4	0.58± 0.60
AS	410	15	30	27.4 ± 0.6	8.5	0.82± 0.51
LS	1000	60	30	33.6 ± 0.6	12.0	0.91± 0.34
CS	580	30	30	28.4 ± 1.2	5.4	1.04± 0.46
HS	625	15	30	32.5 ± 0.4	8.2	3.98± 0.58
Unit-Assembly	-	-	150	149.4± 1.6	0.4	3.98± 0.58

1) Expected (Value) is the thickness based on spin curve section, and Actual (Value) is profilometer data.
2) Error ($= \frac{|Actual - Expected|}{Expected} \times 100$) is calculated based on average value
3) Roughness is surface height profile from the mean line over a given evaluation length and provides a statistical measure of surface variations
4) Roughness is the actual measured lateral range from 5 to 15mm.
5) Sample number is 3.

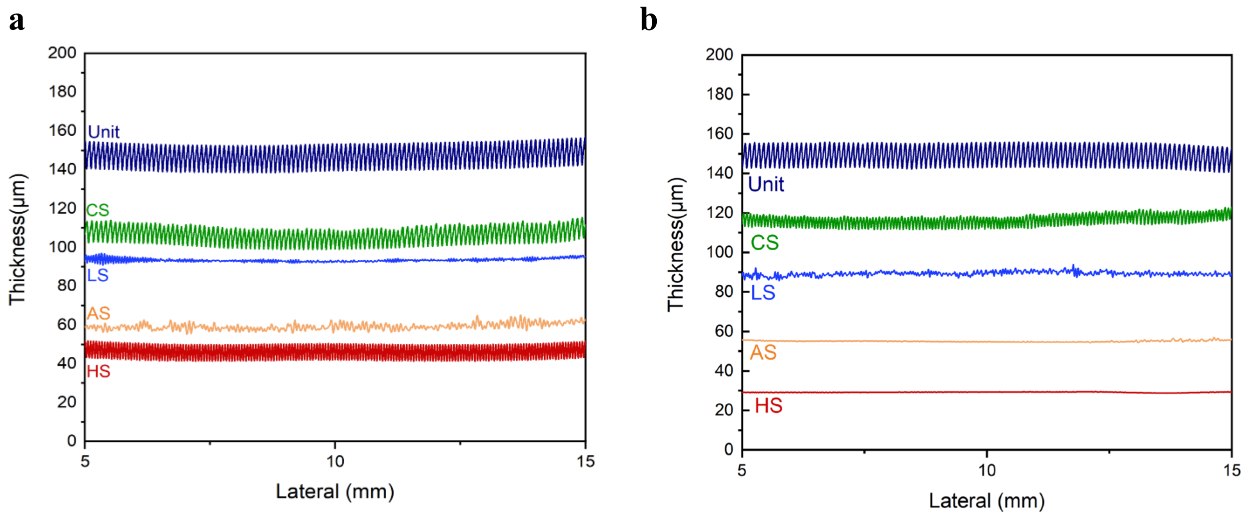

Figure S19. Profilometer data of unit assembly and each layer of uniform (a) 150μm variable layer-assembly and (b) 150μm constant layer-assembly

Section S8. PEDOT:PSS Hydrogel Electrode Fabrication and Characterization

To easily integrate our hydrogel-based power source with current solid-state electronics, we use PEDOT:PSS hydrogel as electrodes (**see Experimental Method**). PEDOT:PSS is a p-type polymer which enables efficient ionic to electrical conversion [13]. PEDOT:PSS ionic to electrical conversion occurs due to its morphological arrangement of a colloidal mixture between PEDOT and PSS [14-16]. PEDOT is a positively charged redox active conjugated polymer enabling electric transport while PSS is a negatively charged polymer that primarily promotes ionic mobility of the free ions from the electrolyte into the PEDOT:PSS colloidal matrix to electrically neutralize the matrix upon reaction [14-16]. Upon oxidation, the free ions are expelled from the PEDOT:PSS matrix as the PEDOT is oxidized [14-16]. By incorporating PEDOT:PSS hydrogel electrodes into our system, we have a complete power source that is environmentally stable, compliant, and able to interface with any electronic component (**Figure S18**). Additionally, the PEDOT:PSS hydrogel and the hydrogels composing the power source can all be spin-coated on top of each other with layer-by-layer (LbL) spin coating (**Figure S18a,b**).

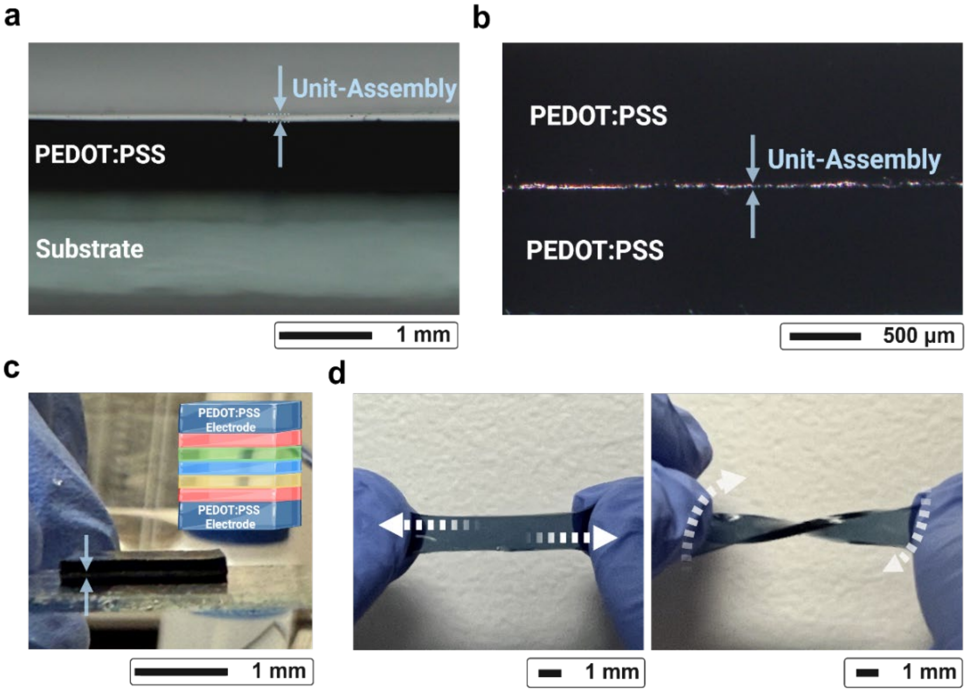

Figure S20. Demonstration of layer-by-layer (LbL) spin coating of 100 µm hydrogel power source unit on top of PEDOT:PSS hydrogel electrode. (a) Side view of 100 µm hydrogel unit spin coated on top of PEDOT:PSS hydrogel. (b) Zoom-in side view of complete hydrogel power source with electrodes. (c) Zoom-out isometric view of hydrogel power source with electrodes. (d) Hydrogel power source with electrodes remains compliant, flexible, biocompatible, and biodegradable.

We also assessed the interfacial robustness between our PEDOT:PSS hydrogel electrodes and our hydrogel power source. We fabricated three units with 150 µm constant layer thickness. We measured the pre-cycling and post-cycling impedance after 50 charge-discharge cycles at a constant current of 10 µA and 500 nA, respectively. We observe that there is a minimal change in impedance post cycling, indicating that the interfacial contact between the PEDOT:PSS hydrogel and the hydrogel power source is fully in contact and robust (**Figure S21**).

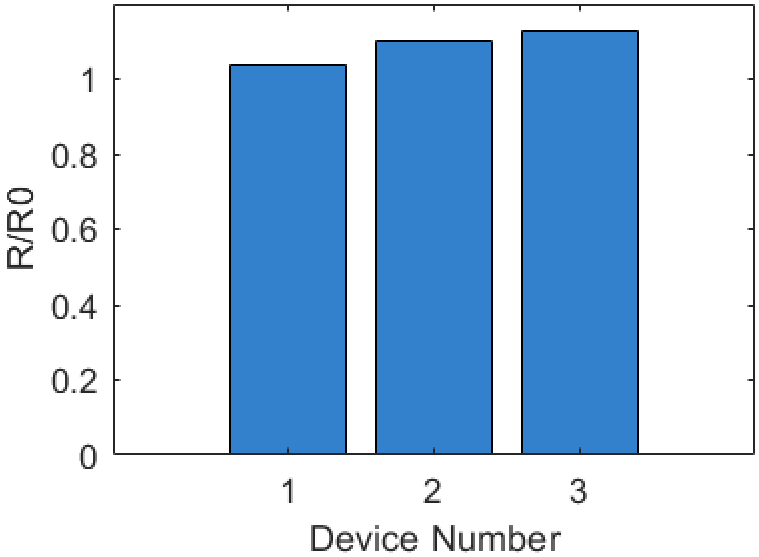

Figure S21. Normalized impedance of three 150 μm units with constant layer thickness after 50 cycles of charge-discharge cycling at 10 μA and 500 nA, respectively. The impedance post cycling is very similar to the impedance pre-cycling indicating there is a robust interfacial adhesion between the PEDOT:PSS hydrogel electrodes and our hydrogel power sources.

For electrical measurements of our hydrogel-based power source, we characterize the cyclability and reversibility of the PEDOT:PSS hydrogels by performing cyclic voltammetry (CV) curves. We constructed a 3-electrode chemical setup: our working electrode is PEDOT:PSS hydrogel with a platinum wire (diameter: 0.25 mm) as current collector, our reference electrode is a standard Ag/AgCl reference electrode, our counter electrode is a platinum wire (diameter: 1 mm), and our electrolyte solution is 3 M LiCl. We soaked the PEDOT:PSS hydrogel in the electrolyte solution for 30 minutes before starting the CV runs. The electrolyte concentration is chosen to be lower than the salt concentration in the high salinity gel (3.5 M), with which the PEDOT:PSS electrodes interface, to account for some of the inefficiencies and resistance caused by the hydrogel crosslinking network. The center-to-center distance between the working PEDOT:PSS electrode and the Ag/AgCl reference electrode is 4.5 cm and has an average ohmic resistance of 43.3 ± 5.7 kΩ (n = 3). The PEDOT:PSS hydrogel is cycled from -0.8 V to 1 V in reference to Ag/AgCl electrode at a scan rate of 0.15 V/s. The PEDOT:PSS hydrogel is both reversible by having cathodic (0.35 - 0.4 V) and anodic (-0.15 V) peaks as well as cyclable based on the consistent trend observed for 20 cycles (**Figure S22**).

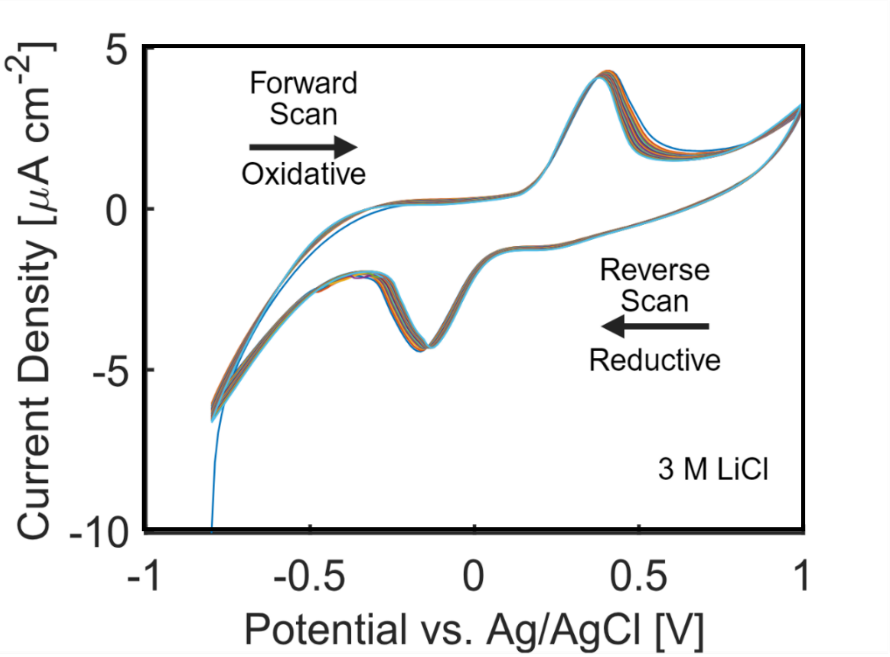

Figure S22. 20 cyclic voltammetry (CV) curves for PEDOT:PSS hydrogel electrode in 3 M LiCl solution at a scan rate of 0.15 V/s. Peaks are observed at 0.4 V and -0.15 V.

Additionally, we assessed the long-term cyclability of our PEDOT:PSS hydrogel by performing 1000 CV scans. We observe that the hydrogel begins to lose its cycling stability after about 250 cycles, with the peaks fully dampening by the 500th cycle (**Figure S23**). The decline in cyclability is due to the swelling of the PEDOT:PSS hydrogel in the electrolyte solution, which reduces the active surface area of the electrode [22] and traps unused ions, leading to less efficient ionic-to-electrical conversion [23]. Compared to our power source and other electric-fish-inspired hydrogel power sources, which often experience significant capacity loss within the first few cycles [9], our current PEDOT:PSS hydrogel formulation offers good long-term cyclability and reversibility. Moreover, studies have shown that PEDOT:PSS hydrogels can maintain cycling stability over 1000 to 20,000 cycles [24-25], a durability that can be leveraged in future electric-fish-inspired power sources once their longevity is further optimized.

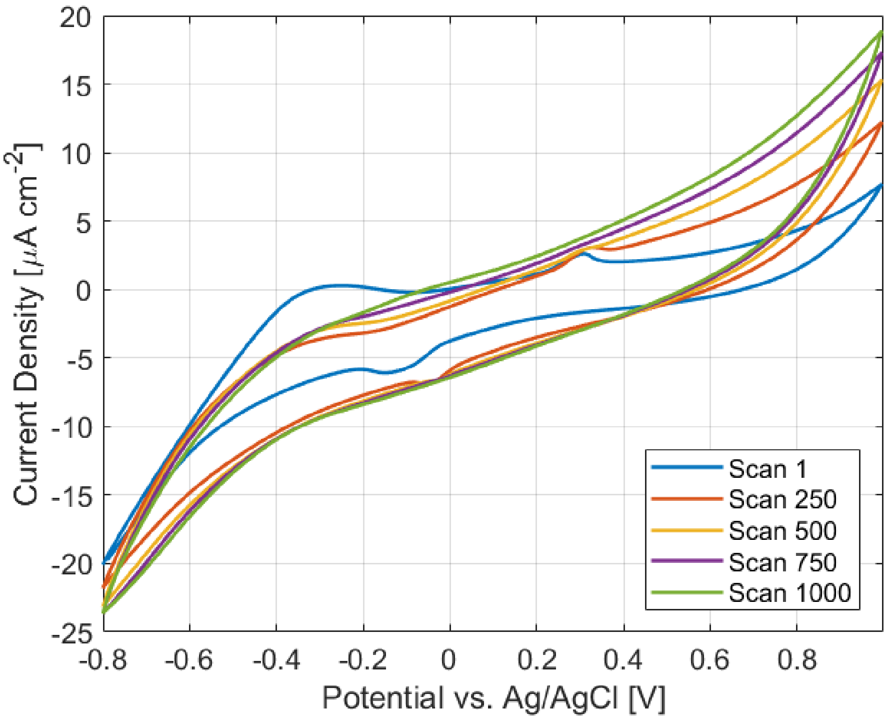

Figure S23. Long-term CV curves of PEDOT:PSS electrodes. The cathodic and anodic peaks begin to dampen after 250 cycles and fully dampen by cycle 500 due to hydrogel swelling reducing the active electrode surface area and increasing the amount of trapped unused ions.

Section S9. Electrical Characterization

In this section, we summarize the electrical characterization of the hydrogel-based power source at 100 μm total unit thickness and at 150 μm total unit thickness with constant and variable layer thicknesses. Section S9.1 explains the electrical metric calculations used by previous literature, which we use to compare our power source metrics to theirs (**Table 1, Figure 5a**). Section S9.2 discusses the various methodologies used to characterize the hydrogel-based power sources as an electrochemical system and to derive additional electrical metrics such as energy density (Experimental Method). A summary of key observations is discussed here:

(1) The self-discharge and discharge profiles of these hydrogel-based power sources follow an exponential decay trend, as seen in previous work, due to limited selectivity in the selective layers. [5,9] Tholen et al. developed a computational model that analyzes various factors such as selectivity in electric-fish-inspired hydrogel power sources. [9] In this model, they analyze how adjusting the fixed charge concentration impacts the permselectivity of the selective layers. The permselectivity is the ability for each selective layer to transport counter-ions while preferentially inhibiting co-ion movement which can be determined from the model by analyzing the molar flux ratio of counter-ions to co-ions in each selective layer. [9] At lower fixed charge concentration of 0.19 M and 0.25 M for the cation and anion selective layers, the permselectivity was 1.06 and 2.56, respectively. [9] While at higher fixed charge concentrations of 1.5 M and 2 M, the permselectivity increased to 1.32 and 3.45 for the cation and anion selective gels, resulting in a 24.5% and 34.8% increase in permselectivity. [9]

    In terms of electrical performance, the model demonstrates that increasing the fixed charge concentration increases the discharge cycle length at a current density of 20 μA cm^{-2} for gel disks with each gel dimension equating to 18 mm in diameter and 1.8 mm in thickness. [9] At lower fixed charge concentrations, the power source fully discharges at 6.7 hours while at the upper fixed charge concentrations the power source fully discharges at 39.6 hours. [9]

Since our power source is 90x thinner, the discharge duration is much more rapid. However, through future optimizations such as improving selectivity such as increasing the fixed charge concentration and other material properties such as crosslinking density, the discharge duration and operational longevity of the power source can be maintained.

(2) We measure the maximum OCP from our self-discharge profiles. For thin, spin-coated hydrogel-based power sources, self-discharge begins during the fabrication process, as ion transport is spontaneously driven once charge separation is established, which starts when a half-cell (HS-AS-LS hydrogels) is fabricated. Since OCP is a thermodynamic property that only changes with concentration and ionic activity, and not with geometrical changes such as decreasing thickness, we determine the maximum OCP of our system by measuring the OCP of disk-stacked power sources, as there is minimal OCP loss between fabrication and measurement. [11] Therefore, for full transparency, we report in Tables 9.1 the maximum OCP for our thin 100 μm and 150 μm units immediately after fabrication but establish the maximum OCP of our hydrogel system through OCP measurements of the disk-shaped power sources.

(3) We demonstrate the ability to charge and discharge these power sources for multiple cycles and derive the average power density and energy density for each cycle. One major limitation is determining the maximum OCP after recharging is tough to ascertain since it is difficult to calculate or experimentally discern the transition between the overpotentials of our system and the start of our exponentially decaying discharge profile. Future work will utilize more selective separators to improve the discharge profile and capacity retention to make the power source more applicable.

S9.1 | Maximum Power Estimation Based on Ohm's Law

In this section, we describe the equations and calculations previous studies have used to evaluate their hydrogel-based power sources [5, 8-9]. Previous work measured their maximum open-circuit potential (V_{OC}) and internal resistance (R_{int}) and determined their other metrics based on Ohmic relations. [5] Ohm's law is defined as [11]:

$$V = IR \quad (S1)$$

where V is the voltage across the device, I is the current, and R is the resistance. [11]

Resistance is governed by the intrinsic material properties and geometry of the conductive path. it is defined by:

$$R = \rho \frac{L}{A} \quad (S2)$$

where ρ is the resistivity, L is the conductive path length, and A is the cross-sectional area. [11] For constant or minimal material resistivity, the resistance is heavily impacted by the geometrical structure of the system.

The power P is calculated by multiplying the potential by the current. [11] Power can be rearranged with Ohm's law (equation S1) to be represented as the voltage drop V_L dissipated across a load resistor R_L: [5, 11]

$$P = \frac{V_L^2}{R_L} \quad (S3)$$

According to the maximum power transfer theorem, maximum power is achieved when the load resistance (R_L) matches the internal resistance ($R_L = R_{int}$).[**5,8**] Under this condition, the voltage across the load is equal to half the open-circuit voltage: [5, 11]

$$V_L = \frac{V_{OC}}{2} \quad (S4)$$

Substituting Equation (S4) into Equation (S3), the instantaneous maximum power is expressed as:

$$P_{max} = \frac{V_{OC}^2}{4R_{int}} \quad (S5)$$

The instantaneous volumetric maximum power density is then calculated by dividing the instantaneous maximum power by the hydrogel volume, Vol_{gel}.

$$\boldsymbol{PD_{max}} = \frac{\boldsymbol{P_{max}}}{\boldsymbol{Vol_{gel}}} \quad (S6)$$

We utilize these equations to calculate the instantaneous maximum power output of our hydrogel-based power sources to compare against previous work (**Table 1, Figure 5a**).

S9.2 | Electrochemical Characterization

However, the measurement approaches in section S9.1 overlook the full electrochemical behavior of this system.[9] As recently emphasized by Tholen et al., hydrogel-based energy systems are better understood through electrochemical analysis methods rather than resistive models.[9]

We utilize a potentiostat (EmStat4S HR, PalmSens) to comprehensively analyze our self-discharge and maximum open circuit potential (OCP) through open-circuit potentiometry, resistance of our power source through electrochemical impedance spectroscopy (EIS), and charge-discharge capabilities through chronopotentiometry and chronoamperometry.

S9.2.1 Maximum open-circuit potential and self-discharge profiles

Through open circuit potentiometry, we measure the self-discharge profile of our hydrogel-based power source and determine the maximum OCP value. Using the simplified Nerst equation (S7) to calculate the potential of our hydrogel-based power source, we determine the maximum OCP that we can achieve based on our final concentrations (HS: 3.5 M, LS: 0.035 M) is 236 mV. [11]

$$E = \frac{RT}{zF} \ln\left(\frac{C_{HS}}{C_{LS}}\right) \quad \text{(S7)}$$

E is the potential, R is the universal gas constant, T is the temperature, z is the valence of the ion charge (in this case z = 1), and C is the concentration of the high salinity (HS) and low salinity (LS). [11] However, this estimation assumes that our solvent is pure water and not a polymer network with a glycerol mixture, that our ionic activity coefficients are ideal and equal to one, both of our high and low salt concentrations are fully diluted, and that our selective membranes provide identical half-cell potentials. [11]

In our system, we measured the maximum OCP to be an average of 193 ± 18 mV, which is about 40 mV below the theoretically calculated maximum OCP. We reviewed the general Nerst equation (equation S8) to determine which of the assumptions above may lead to a reduction in OCP.

$$E = \frac{RT}{zF} \ln\left(\frac{a_{HS}}{a_{LS}}\right) = \frac{RT}{zF} \ln\left(\frac{\gamma_{HS} b_{HS}}{\gamma_{LS} b_{LS}}\right) \quad \text{(S8)}$$

Where a is the activity of the high salinity and low salinity, in which activity is defined as the activity coefficient (γ) of the ions multiplied by the molality (b) of the gel type, which can be determined from the known concentration. [11] From the general Nerst equation, we determine our reduction in OCP is due to our non-fully dilute salt concentration, which leads to high ionic strength limiting ion mobility. Additionally, our hydrogel composition consists of glycerol, which increases the viscosity and further impedes ion mobility. Therefore, practically, the activity coefficient in our system is less than one and lowers the maximum achievable OCP. Future electromotive force (EMF) experimentation will help quantify the activity of our system to determine improvements.

Significantly, our maximum OCP of 193 ± 18 mV is based on the hydrogel disk-stacked power sources, as the time between assembly for this configuration of the power source and measurement is negligible. For our thin-stacked spin-coated power source, self-discharge begins during our fabrication process as soon as a half-cell is fabricated, since the movement of ions is a thermodynamically spontaneous process (**Figure S24**). Thus, by the time measurements of the spin-coated unit are taken, about 5-10 min. have passed, and with our rapid discharge profile, identifying the maximum OCP is difficult (more information in section 9.2.4). Therefore, to properly determine the maximum OCP of our hydrogel-based power source, we use a disk-stacked unit (**Table 9.1**).

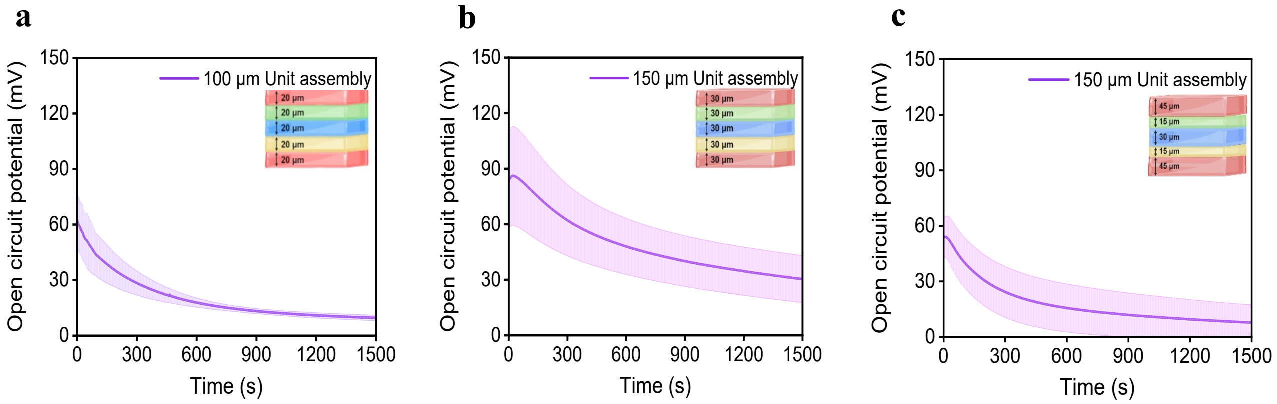

Figure S24. Self-discharge plots for (a) 100 µm unit (20|20|20|20|20 µm). (b) 150 µm unit with uniform configuration (30|30|30|30|30 µm). (c) 150 µm with variable configuration (45|15|30|15|45 µm). All self-discharge plot show an exponential decay trend with the maximum open circuit potential (OCP) ranging from 60-90 mV rather than the expected maximum OCP being around 190 mV due to self-discharge starting during fabrication. The maximum OCP for the 150 µm unit in (b) had a higher variation in maximum OCP as it was the easiest to handle post fabrication, and therefore shows a higher maximum OCP since we measure more of the self-discharge (n = 4 samples)

9.1. Average maximum open circuit potential (OCP) for hydrogel disks with different total thicknesses

Disk unit-assembly thickness	2.5 mm disk unit-assembly (0.5 | 0.5 | 0.5 | 0.5 | 0.5)	9 mm disk unit-assembly (1.8 | 1.8 | 1.8 | 1.8 | 1.8)	25 mm disk unit-assembly (5 | 5 | 5 | 5 | 5)
Maximum OCP [mV] (n=3)	190 ± 24	187 ± 18	203 ± 8
Total Average [mV]	193 ± 18		

S9.2.3 Resistive and capacitive components

We perform electrochemical impedance spectroscopy (EIS) to determine the resistance of each hydrogel type and the entire hydrogel unit. We obtain the ohmic resistance of our sample by fitting the Nyquist plot to Randles circuit model. The ohmic resistance is the first resistance in the model that is in series with the Warburg-RC circuit section. The ohmic resistance can also be determined by the minimum point in the Nyquist plot as at this frequency, no capacitive effects and minimal mass transfer effects between the system and the electrode are observed. [12] **Figure S25** demonstrates the Nyquist plot for multiple samples, and **Table 9.2** summarizes the results. The experimental section in the main manuscript details the frequency range and electrodes used to measure EIS.

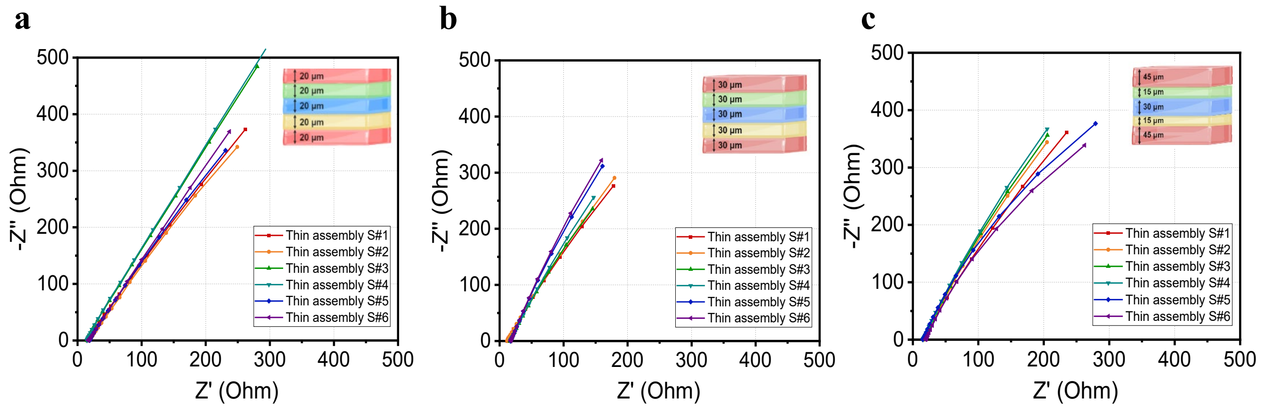

Figure S25. Nyquist plots derived from EIS measurements for (a) 100 µm unit (20|20|20|20|20 µm). (b) 150 µm unit with uniform configuration (30|30|30|30|30 µm). (c) 150 µm with variable configuration (45|15|30|15|45 µm). (n = 6 samples)

9.2. Summary of measured resistance and OCP after fabrication for 100 µm and 150 µm units

Type	Resistivity4) 5) (Ωm)	Resistance5) (Ω)	Area Normalized Resistance5) (Ωm^2)	Maximum open-circuit potential after fabrication6) 7) (mV)
100 µm Unit-ssembly1) (20|20|20|20|20)	14.40 ± 4.34	11.75 ± 1.73	(2.00 ± 0.40) × 10^{-3}	62.7 ± 15.5
150 um Unit-Assembly2)	18.38 ± 1.66	16.37 ± 2.31	(2.74 ± 0.25) × 10^{-3}	86.6 ± 34.3

(30|30|30|30|30)				
150 um Unit-Assembly[3)] (45|15|30|15|45)	16.52 ± 1.71	16.59 ± 1.62	(2.51 ± 0.26) X 10^{-3}	54.8 ± 11.0

[1)] Unit thickness of unit-assembly is 106.1 μm and cross-sectional area is 1.71 × 10^{-4} m^2.
[2)] Unit thickness of unit-assembly is 149.4 μm and cross-sectional area is 1.70 × 10^{-4} m^2.
[3)] Unit thickness of unit-assembly is 151.5 μm and cross-sectional area is 1.51 × 10^{-4} m^2.
[4)] Resistivity is calculated ($= \text{Resistance} \times \frac{\text{Cross-sectional Area}}{\text{Unit thickness}}$).
[5)] Sample number is 6.
[6)] Sample number is 4.
[7)] Maximum open-circuit potential was figured out based on discharge curve right after fabrication.

S9.2.4 Charge-discharge curves

We performed constant voltage and constant current charge-discharge curves to assess whether our power source is rechargeable and cyclable, as well as to quantify the energy delivered by the system. Self-discharge curves under constant voltage were conducted to evaluate the system's rechargeability and to determine the current level needed for galvanostatic discharge at a constant current (**Figure S28b, S28d**). The constant voltage charging potential was set at 200 mV for three different charge durations, which were either based on reaching specific current cutoffs (10 μA, 1 μA, 0.1 μA) or a fixed time period (10 hours), determined through trial and error. We observed that all self-discharge curves tend to reach the same plateau value, which is set at the minimum potential cutoff value (1 mV). However, as the constant voltage charging duration increases, self-discharge duration also increases, such that at 10 μA current cutoff, the self-discharge rate is the fastest, and for the 0.1 μA current cutoff, the self-discharge rate is the slowest (**Figure S28b, S28d**). This observation indicates that our hydrogel power system is rechargeable, as the self-discharge duration increases. However, since all self-discharge profiles reach the same plateau level, our selective membranes cannot maintain the charge separation and discharge quickly. Additionally, we believe that our significant initial potential drop occurs from ion rearrangement, in which local concentration gradients are built up during charging and disappear when charging stops. Future work with more selective membranes and separators will help characterize the loss of potential to ion rearrangement, as well as identify other selective membranes to improve the self-discharge profile and duration.

To evaluate specific electrical metrics and demonstrate the cyclability of our power source, we performed constant-current charge-discharge curves (**Figures S26 and S28**). Initially, we recorded the self-discharge immediately after fabrication, followed by charging at a constant current density of 6 μA m^{-2} until reaching a potential cutoff of 400 mV. Then, we discharged at a constant current density of 0.03 μA m^{-2}. The first charge-discharge cycle recovers the highest capacity and has the longest discharge duration, while subsequent cycles follow the same charge-discharge profile but last for shorter durations than the first cycle. This pattern aligns with charge-discharge simulations previously analyzed for hydrogel-based power systems. [9] From the constant discharge curves, we derive additional metrics such as average power density and energy density for each cycle and compare these metrics between power sources with constant and variable layer thicknesses (**Figures S26-S29**). We developed a custom MATLAB script to analyze the constant current discharge curves for each cycle. The calculations of the various metrics are as follows. The instantaneous discharge power output is calculated by multiplying the constant current by the potential:

$$P_{discharge}(t) = I(t) * V(t) \quad \text{(S8)}$$

The average volumetric power density (*PD*) for each cycle is calculated by dividing the average discharge power by the hydrogel volume Vol_{gel}.

$$PD = \frac{\left(\frac{1}{t_f}\sum_{t=1}^{t_f} P_{discharge}(t)\right)}{Vol_{gel}} \quad \text{(S9)}$$

The total energy output of the system is calculated by integrating the instantaneous power over the discharge time.

$$E = \int_{t_i}^{t_f} P(t)\, dt \quad \text{(S10)}$$

Energy density (ED) is then calculated by the total energy output normalized by the hydrogel volume Vol_{gel}:

$$ED = \frac{E}{Vol_{gel}} \quad \text{(S11)}$$

As we expected, for each cycle, the average power density remains constant, but the energy density is much larger in the first cycle and then plateaus in subsequent cycles (**Figures S27**). Additionally, we observe that the average power density is in *milli*watts m^{-3} compared to our instantaneous maximum power density which is in *kilo*watts m^{-3} due to the discharge profile following an exponential decay. When we compare hydrogel-based power sources with constant layer thickness versus variable layer thickness, we observe that both our instantaneous maximum and average power density are very similar. However, the energy density for units with the same volume but with variable layer thickness is 180% higher than for units with the constant layer thickness due to its increase in salt capacity (**Figure S29**).

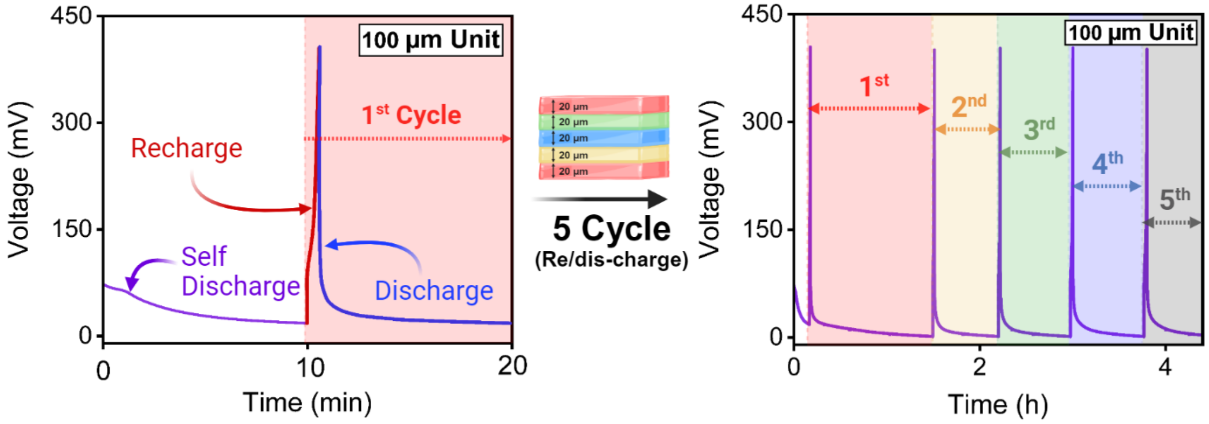

Figure S26. Constant current charge discharge curves for the 100 µm power source unit. The left side shows a zoom-in version of the full 5 cycles on the right to improve the visualized transition between self-discharge, recharge, and discharge. The first charge cycle has the most capacity as can be observed by it large discharge duration compared to the other cycles in right figure.

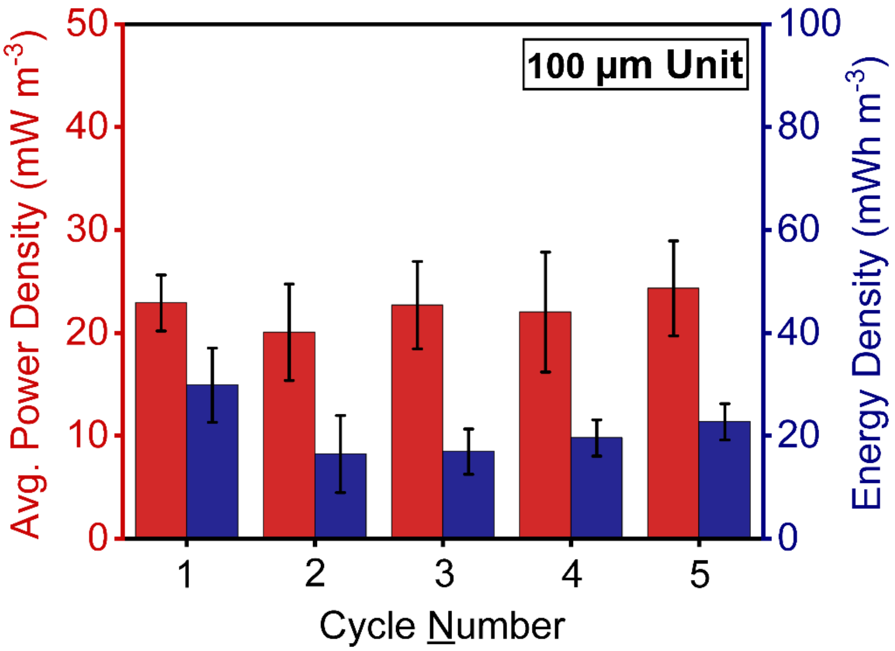

Figure S27. Average power density and energy density of the 100 µm unit for each discharge cycle shown in Figure S26. The average power density remains relatively constant between cycles but the energy density decreases after the first cycle and plateaus for the other cycles.

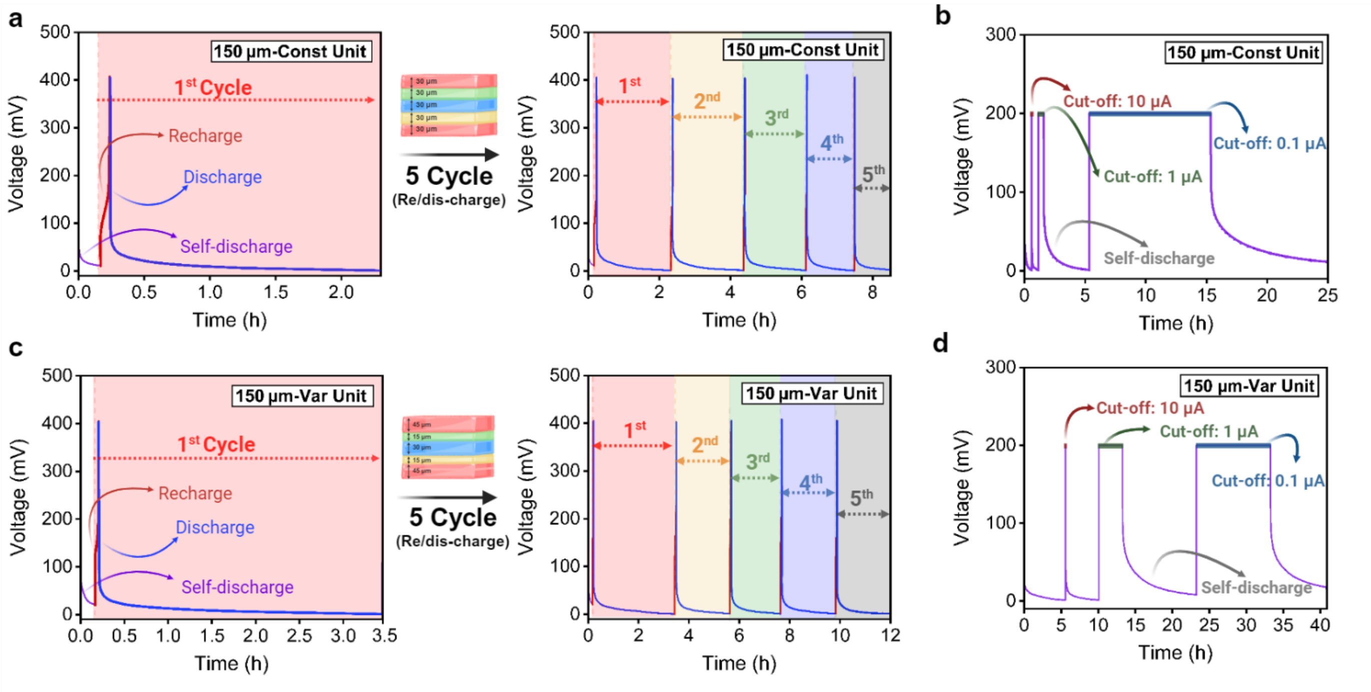

Figure S28. (a) Constant current discharge curves for the 150 µm unit with constant layer thickness. The left side shows a zoomed-in image of the first 2 hours of the 5-cycle charge-discharge curve on the right. Similar observations are seen here as seen with the 100 µm unit is Figure S26. (b) Constant voltage curves for the 150 µm unit with constant layer thickness. We demonstrate the ability to retain charge as our self-discharge curves increase in duration, as the constant potential is applied for longer. (c) and (d) are constant current and voltage curves for the 150 µm unit with variable layer thickness. Similar observations to the 150 µm unit with constant layer thickness and the 100 µm unit are observed.

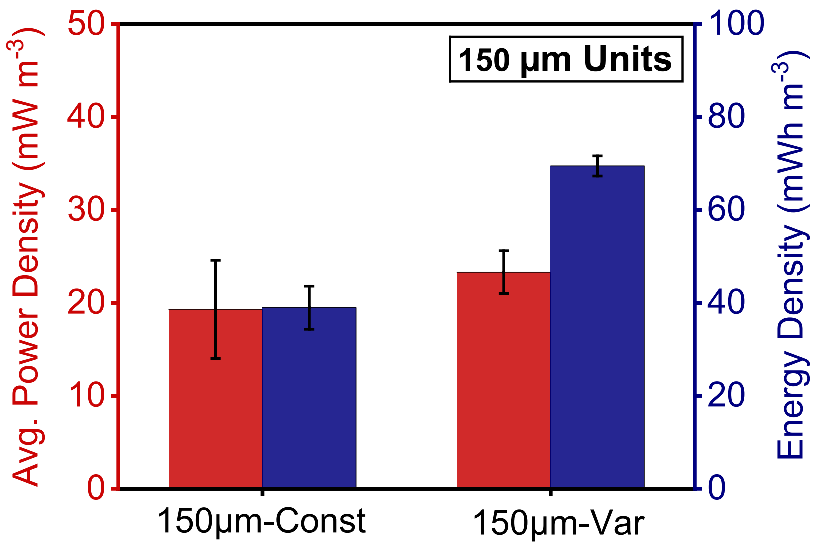

Figure S29. Comparison of average power density and energy density for the 150 µm constant layer thickness versus variable layer thickness. We observe a similar average power density between the constant and variable 150 µm units but a significant improvements in the energy density of the variable 150 µm unit. The mean and standard deviation here, are the average power density and energy density derived from the first discharge curve for 3 different samples (n = 3 samples).

To further characterize the operational usage of this power source, we provide additional discharge curves at various constant currents of 50 nA, 500 nA, 5 µA, 50 µA, and 23 mA (**Figure S30**) for the 150 µm unit with constant layer thickness to simulate operation under various load resistors. We observe that as the applied constant current increases, the discharge duration decreases. The discharge profile mechanically describes the rate of ion transport through the gels with the duration corresponding to the time required for the concentration of the salinity layers to equilibrate. [9] Notably, we observe that at 50 µA constant current, the discharge duration is less than 0.5 seconds as the higher current transport ions faster and reach equilibrium faster. At 23 mA constant current, the discharge rate was too fast to be recorded based on our operating window range of 0 – 400 mV, even at higher sampling frequencies.

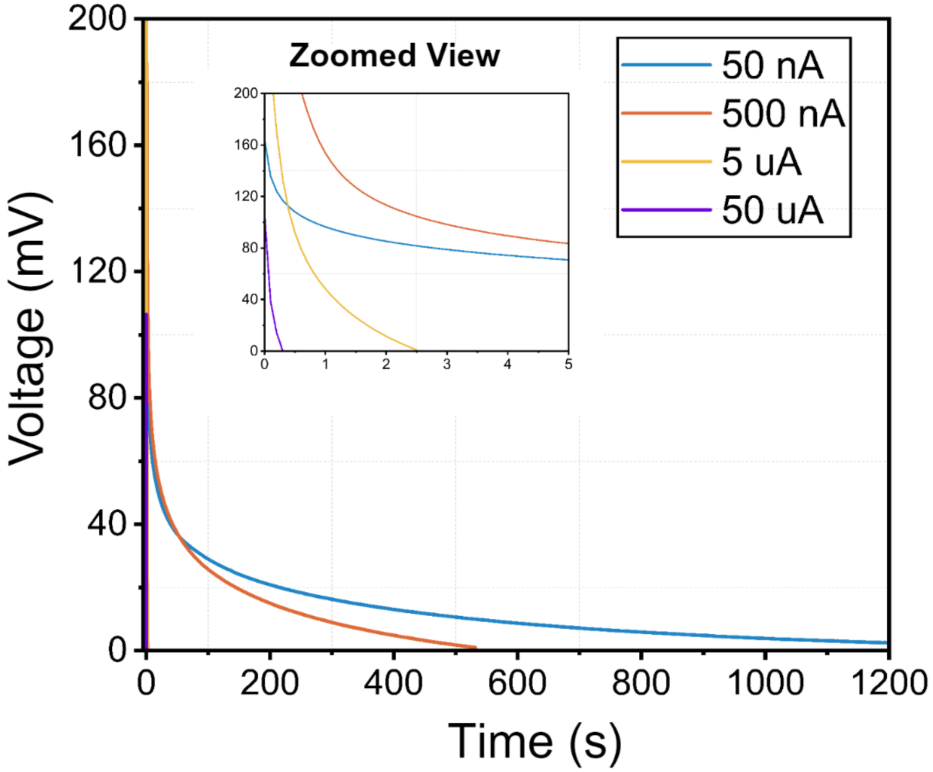

Figure S30. Single traces of constant current discharge curves of the 150 µm units with constant layer thickness at various constant currents (50 nA, 500 nA, 5 µA, and 50 µA). 23 mA constant current discharge dissipated too rapidly to be recorded. As the constant current magnitude increase the discharge duration decreases due to faster ionic transport towards equilibrium.

Additionally, we derived an IV curve based on the discharge curves in **Figure S30**. To form the IV curve, we averaged the potential for three different units at the same constant current over a selected absolute voltage range (38-152 mV) which is within 20-80% of a fixed starting potential which we set to our

maximum measured OCP of 190 mV. We chose an absolute voltage range with a fixed reference potential as it provides a more direct comparison between the various constant currents rather than normalizing each curve to its own initial potential. Furthermore, normalizing the curve to its own initial potential is difficult as the nonlinear decay makes it challenging to distinguish between the overpotentials and the start of the discharge profile.

Figure S31 demonstrates that as the applied constant current increases, the average potential measured within the fixed potential window also increases. This increase in average potential is caused by the nonlinear decay of the potential. At lower constant currents, the potential decays more gradually and a substantial portion of the discharge trend is within the lower portion of the fixed potential window, resulting in a lower average voltage. On the other hand, at larger constant currents, although the potential decay is steeper, the potential includes higher absolute values in the same fixed voltage range, resulting in a higher average voltage. Thus, the average voltage increases with larger constant currents. Since our power source is ionically driven, the discharge duration in **Figure S30** and the IV curve demonstrate the dependance of faster ionic transport increasing with current.

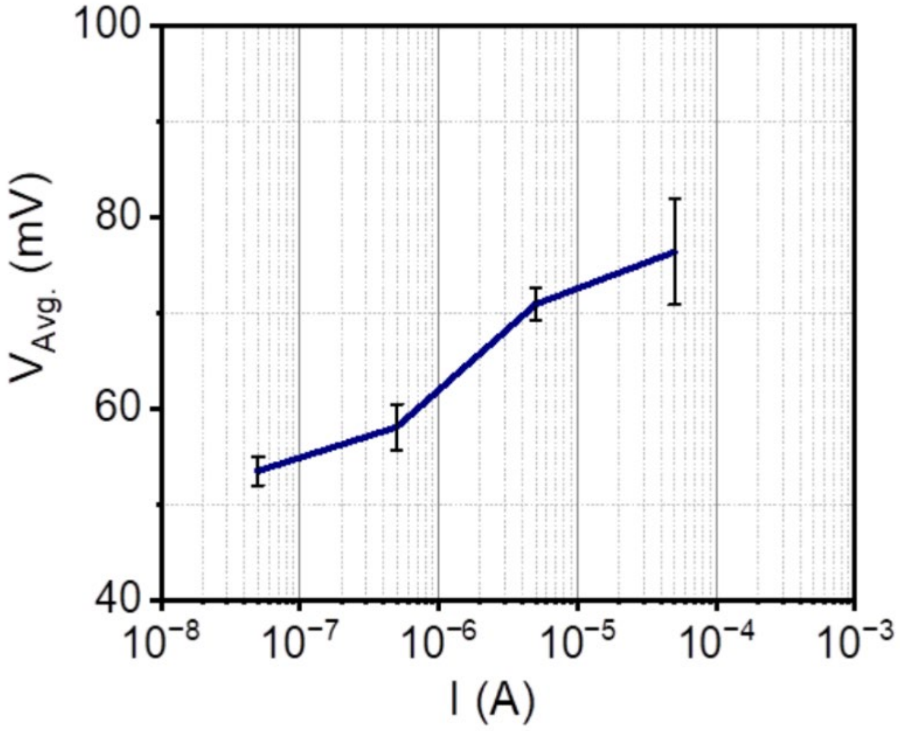

Figure S31. IV curve of the 150 μm unit with constant layer thickness in an absolute potential window range of 38-152 mV with a common fixed initial potential of 190 mV. The average potential is lower at lower constant currents as a substantial portion of the discharge trend is in the lower portion of the fixed potential window. The average potential is higher at larger constant currents, as the potential magnitude is larger within the fixed potential window, regardless of the steep discharge rate.

Moreover, we generate constant power density output curves based on the average voltage values from **Figure S31**. We determine the average power output by multiplying the average potential by the constant current applied in the selected potential range. Since our average potential magnitudes are relatively similar in magnitude but constant currents vary significantly in order of magnitude, the power output curve varies exponentially based on the constant current applied, as seen in **Figure S32**. These results further support that at higher constant current and higher power output, we will have faster discharge rates as the ions equilibrate much more rapidly which can be observed in the demonstration in section S9.3.

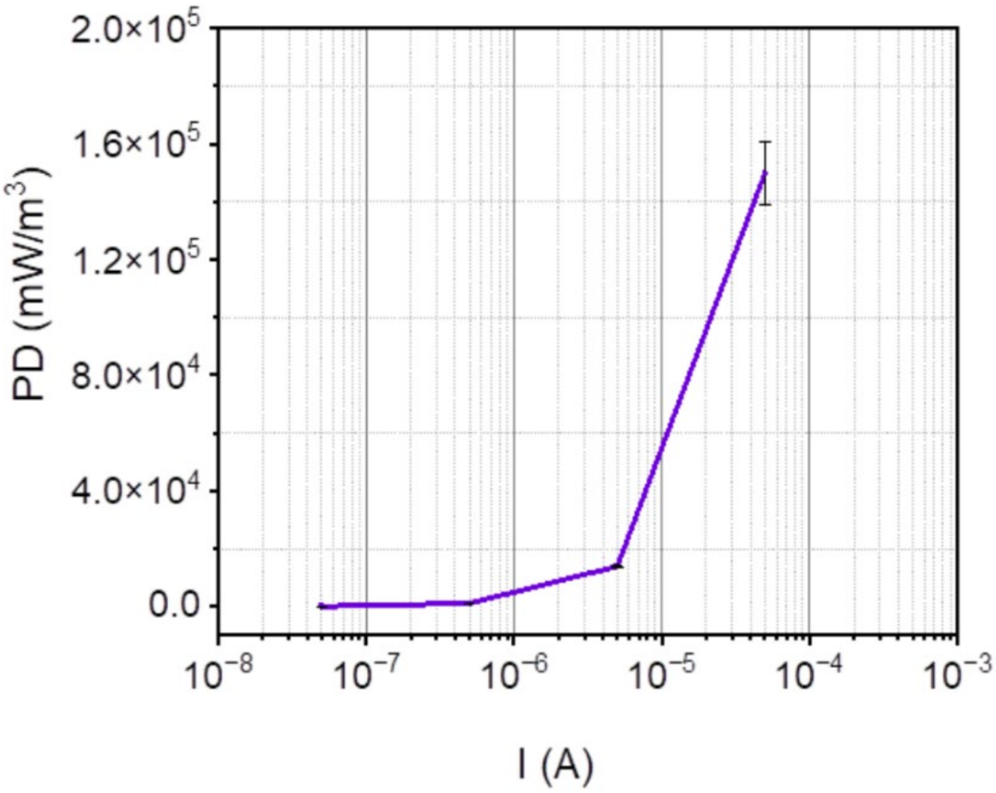

Figure S32. Average power density the 150 μm unit with constant layer thickness in an absolute potential window range of 38-152 mV with a common fixed initial potential of 190 mV. The average power density is calculated by multiplying the average potential by the constant current. Since the average potential magnitudes are relatively similar but the constant current magnitudes increase significantly in order of magnitude, the average power density curve increase exponentially with increase constant current. This trend supports that our discharge rate will be faster at higher required power output.

S9.3 | Demonstration

To demonstrate the scalability of our fabrication method and capability of supplying sufficient power to common electrical components, we spin-coated ten 100 μm hydrogel-based power source units in series to reach a potential of 2 V (**Figure S33**). After fabrication, the maximum open-circuit potential was around 600 mV, which is about 10 times the maximum open-circuit potential of a single hydrogel-based power source unit right after fabrication. We then charged our in-series hydrogel-based power source and connected it to a 2V red LED. Still images show the LED off while the power source is recharging after fabrication and on once recharging is complete. However, due to the fast discharge rate of the hydrogel-based power source, the LED turns on for only half a second before turning off as the potential drops below the level needed to power it. Here, we demonstrate that we can briefly power an LED with this fabrication

method, but further improvements to the discharge profile of the hydrogel-based power source are necessary to enhance its practicality.

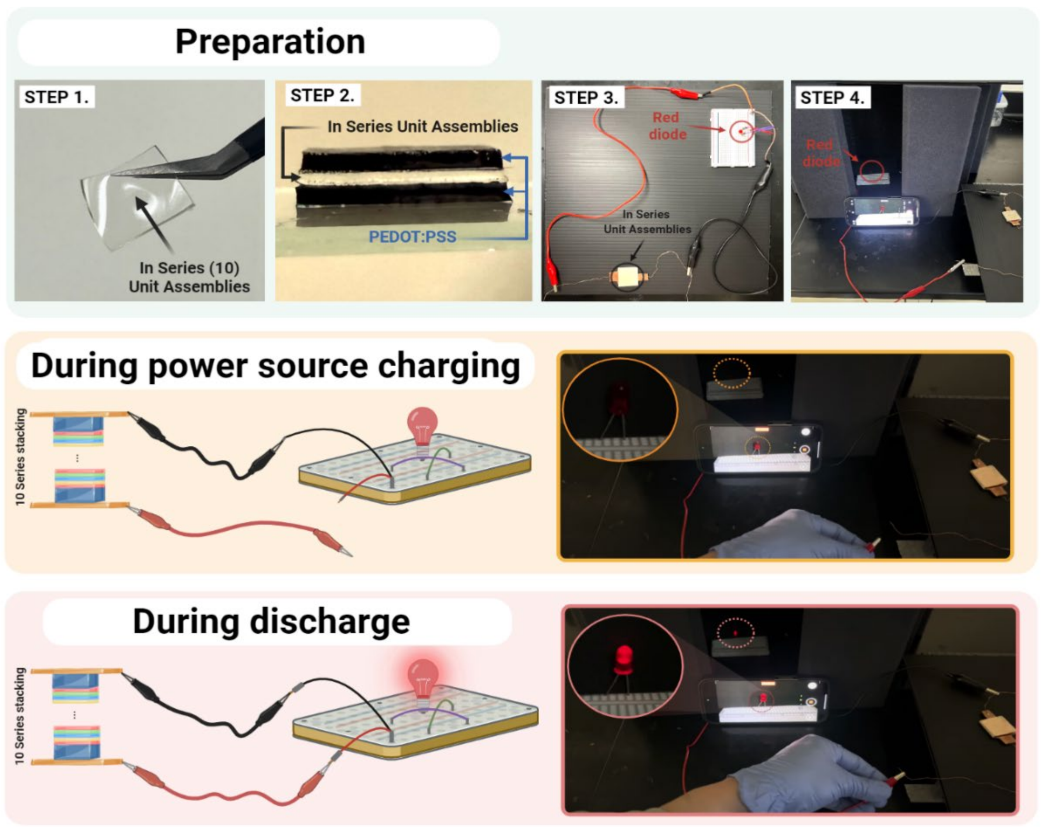

Figure S33. Fabrication of ten 100 µm units stacked in series to power a red LED. When the power source is recharging after fabrication, the light is off. When the power source finishes charging, we switch the circuit for the battery to interface with the LED to turn it on.

Section S10. Comparison of this work to current flexible microbatteries

We benchmarked our proposed system against existing classes of flexible microbatteries. Broadly, these devices can be divided into two categories: partially polymeric microbatteries, in which conventional lithium- or zinc-based batteries incorporate a polymeric anode, cathode, or electrolyte; and fully polymeric microbatteries, in which all active components, the anode, cathode, and electrolyte are polymeric solutions or gels.

In terms of electrical performance, partially polymeric microbatteries generally exhibit much higher power and energy densities (typically in the mW/mWh range) but rely on potentially hazardous materials and often involve complex, intricate fabrication methods. In contrast, fully polymeric microbatteries typically provide power and energy densities one to two orders of magnitude lower (from nW/nWh to µW/µWh), and their fabrication processes are still largely manual. However, their material compositions are usually fully biocompatible.

Compared with these systems, our hydrogel-based power source achieves an instantaneous maximum power density on the same order of magnitude as partially polymeric devices, while our average power density and energy density fall within the lower end of fully polymeric systems due to the inherently rapid discharge behavior. Nonetheless, our device footprint is significantly smaller than other fully polymeric power sources and our fabrication method is semi-automated. Additionally, whereas most fully polymeric systems still require metallic electrodes such as Ag/AgCl or carbon-aluminum interfaces to connect to external electronics, our approach employs PEDOT:PSS hydrogel electrodes, enabling a completely polymeric system that can interface seamlessly with both metallic and soft components.

10. Comparison of flexible microbatteries

Ref	Battery (electrode)	Electrodes	Electrolyte	Thickness	Fabrication method	Electrical performance	Fully polymeric
26	Zn‖$Zn_{0.25}V_2O_5*nH_2O$ (ZVO) battery	Zn‖$Zn_{0.25}V_2O_5*nH_2O$ (ZVO)	3 M $ZnSO_4$ in eletrogelated chitosan hydrogel (E-CS-GE)	200 µm	In situ electrogelation	PD: 18.3 Wh Kg^{-1} ;	No
27	Solid state graphene-based micro-supercapacitor	Graphene Oxide (GO) ‖ Gold (Au)	H_2SO_4-PVA hydrogel	~15 nm	Photolithography etching	PD: 1270 W cm^{-3}<br>ED: 3.6 mWh cm^{-3}	No
28	Aqueous-based zinc batteries	Zn‖MnO_2 printed on flexible PET	2 M $ZnSO_4$ (Aq) and 0.5 M $MnSO_4$ (Aq)	6.4 – 9.8 µm	Screen printing	PD: 150 mW cm^{-3}<br>ED: 17.3 mWh cm^{-3}	No
29	Interdigitated Lithium ion microbattery	$Li_4Ti_5O_{12}$ (LTO) ‖ $LiFePO_4$ (LFP)	1 M $LiClO_4$ in 1:1 ethylene carbonate:dimethyl carbonate solution	128 µm (8 layers, ~30 µm per layer)	3D printing	PD: 2.7 mW cm^{-2}<br>ED: 2.69 mWh cm^{-2}	No
30	3D Silicone array lithium ion microbattery	Anode: crystalline silicone<br>Cathode: slurry composed of NCA, graphite, PVDF, LiTFSI, TG, and PEO	1 M $LiClO_4$	400 µm	Photolithography - Silicone dry etching	PD: ~0.3 mW cm^{-2}<br>ED: 5 mAh cm^{-2}	No
31	Aqueous zinc ion batteries	Zn‖MnO_2	2 M $ZnSO_4$ and 0.2 M $MnSO_4$	74 µm	Screen printing and electrodeposition	PD: ~120 mW cm^{-2}<br>ED: 5.27 mWh cm^{-2}	No
32	Quasi-solid-state zinc ion batteries (CaV_3O_7 nanobelts)	Zn‖Binder-free CaV_3O_7 nanobelts	1 M $ZnSO_4$ /PVA hydrogel	0.76 mm	Packing/manual assembly	PD: 170.9 mW cm^{-3}<br>ED: 9.2 mWh cm^{-3}	No
33	Quasi-solid-state zinc ion batteries (V_2O_5 nanorods)	Carbon cloth (CC) Zn ‖ CC/oxygen defect modulated binder-free V_2O_5 nanorods	1 M $ZnSO_4$/PAM hydrogel	0.7 mm	Packing/manual assembly	PD: 33.4 mW cm^{-3}<br>ED: 10.5 mWh cm^{-3}	No
34	Moisture Enabled water generator (MEWG) through hydrogel heterojunction	Silver plates (Ag)	PAAS polyanion (PA)‖DMAPAA-Q polycation (PC) activated by moisture (20-100% RH)	3 mm	Casting	PD: 0.36 nW cm^{-2}	Yes
35	Asymmetric biohydrogel batteries	Carbon fibers	Na^+, Li^+, or K^+ ions traversing sodium alginate polyanion (PA) ‖ Protonated chitosan polycation (PC)	Variable; not specified	Drop-casting and 3D printing	PD: 135-190 mW m^{-2}<br>(13.5-19 µW cm^{-2})	Yes
36	Bi-ionic gradient battery	Folded carbon-coated aluminum foil (C-Al)	PANa-PVA gel ‖ HAAC-PVA gel (Na^+/Cl^- gradients)	2 mm	Casting	PD: ~8.8 µW cm^{-3}<br>ED: ~16.3 µWh cm^{-3}	Yes
37	Zwitterionic hydrogel power sources	Ag/AgCl	LiCl zwitterionic polymer; anion selective membrane P(BA-r-VBC) ‖ Nafion cation selective membrane	500 µm	Manual stacking	PD: 60 nW cm^{-2}	Yes
This Work	Spin Coated electric-fish-inspired hydrogel power source	PEDOT:PSS hydrogels	3.5 M LiCl (Aq) across APTAC‖AMPS hydrogels	100 µm	Spin coating	Maximum instantaneous PD: 44 kW m^{-3} (44 mW cm^{-3})<br><br>Average PD: 23 mW m^{-3} (23 nW cm^{-3})<br><br>ED: 30 mWh m^{-3} (30 nWh cm^{-3})	Yes

References

1. Han, L. *et al.* Mussel-Inspired Adhesive and Conductive Hydrogel with Long-Lasting Moisture and Extreme Temperature Tolerance. *Adv Funct Materials* **28**, 1704195 (2018).
2. Marques, N., De Morais, S., Da Câmara, P., De Souza, E. & Balaban, R. Gelation Behavior of Polyacrylamide-Polyethyleneimine: Effects of Diethylenetriamine and Glycerol Addition. *J. Braz. Chem. Soc.* (2023) doi:10.21577/0103-5053.20230049.
3. Cazzell, S. A., Duncan, B., Kingsborough, R. & Holten-Andersen, N. Demonstration of Environmentally Stable, Broadband Energy Dissipation via Multiple Metal Cross-Linked Glycerol Gels. *Adv Funct Materials* **31**, 2009118 (2021).
4. Chi-Ucán, S. L. *et al.* Inhibition Effect of Glycerol on the Corrosion of Copper in NaCl Solutions at Different pH Values. *Journal of Chemistry* **2014**, 1–10 (2014).
5. Schroeder, T. B. H. *et al.* An electric-eel-inspired soft power source from stacked hydrogels. *Nature* **552**, 214–218 (2017).
6. Lin, C.-H. *et al.* An investigation of carboxylated chitosan hydrogel electrolytes for symmetric carbon-based supercapacitors at low temperatures. *Journal of the Taiwan Institute of Chemical Engineers* **126**, 324–331 (2021).
7. Szymańska, E. & Winnicka, K. Stability of Chitosan—A Challenge for Pharmaceutical and Biomedical Applications. *Marine Drugs* **13**, 1819–1846 (2015).
8. Guha, A. *et al.* Powering Electronic Devices from Salt Gradients in AA-Battery-Sized Stacks of Hydrogel-Infused Paper. *Advanced Materials* **33**, 2101757 (2021).
9. Tholen, H. M., Taylor, R. F., Hall, D. M. & Najem, J. S. Mechanistic insights into electric fish-inspired power sources: A combined modeling and experimental approach. *Electrochimica Acta* **525**, 146043 (2025).
10. Yllö, Are, and Chao Zhang. Experimental and molecular dynamics study of the ionic conductivity in aqueous LiCl electrolytes. Chemical Physics Letters 729 (2019): 6-10.

11. Lvov, Serguei N. Introduction to electrochemical science and engineering. Crc Press, 2021.

12. Lazanas, Alexandros Ch, and Mamas I. Prodromidis. "Electrochemical impedance spectroscopy─ a tutorial." ACS measurement science au 3.3 (2023): 162-193.

13. Lu, Baoyang, et al. "Pure pedot: Pss hydrogels." Nature communications 10.1 (2019): 1043.

14. Rebetez, Gonzague, et al. "What drives the kinetics and doping level in the electrochemical reactions of PEDOT: PSS?." Advanced functional materials 32.5 (2022): 2105821.

15. Volkov, Anton V., et al. "Understanding the Capacitance of PEDOT: PSS." Advanced Functional Materials 27.28 (2017): 1700329.

16. Romele, Paolo, et al. "Ion buffering and interface charge enable high performance electronics with organic electrochemical transistors." Nature communications 10.1 (2019): 3044.

17. Sui, Xiaojie, et al. "Ionic conductive hydrogels with long-lasting antifreezing, water retention and self-regeneration abilities." Chemical Engineering Journal 419 (2021): 129478.

18. Antipova, Christina G., et al. "A Comprehensive Mechanical Testing of Polyacrylamide Hydrogels: The Impact of Crosslink Density." *Polymers* 17.6 (2025): 737.

19. Wang, Siheng, et al. "Strong, tough, ionic conductive, and freezing-tolerant all-natural hydrogel enabled by cellulose-bentonite coordination interactions." *Nature Communications* 13.1 (2022): 3408.

20. Cheng, Zhenfang, et al. "Entangled hydrogels: physicochemical properties, synthesis and applications." *Pure and Applied Chemistry* 0 (2025).

21. Liu, Huimin, et al. "Super Tough Anti-freezing and Antibacterial Hydrogel With Multi-crosslinked Network for Flexible Strain Sensor." *Small* 21.8 (2025): 2407870.

22. Hütter, Philipp C., et al. "Efficiency of the switching process in organic electrochemical transistors." *ACS applied materials & interfaces* 8.22 (2016): 14071-14076.

23. Savva, Achilleas, Shofarul Wustoni, and Sahika Inal. "Ionic-to-electronic coupling efficiency in PEDOT: PSS films operated in aqueous electrolytes." *Journal of Materials Chemistry C* 6.44 (2018): 12023-12030.

24. Xue, Yu, et al. "Mechanically-compliant bioelectronic interfaces through fatigue-resistant conducting polymer hydrogel coating." *Advanced Materials* 35.40 (2023): 2304095.

25. Lu, Baoyang, et al. "Pure pedot: Pss hydrogels." *Nature communications* 10.1 (2019): 1043.

26. Huang, Jing, et al. "Practical, sustainable, wide-temperature-adaptable zinc-metal batteries enabled by electrogelated recyclable biomacromolecular hydrogel electrolytes." *National Science Review* 12.9 (2025): nwaf308.

27. Wu, Zhong-Shuai, et al. "Photolithographic fabrication of high-performance all-solid-state graphene-based planar micro-supercapacitors with different interdigital fingers." *Journal of Materials Chemistry A* 2.22 (2014): 8288-8293.

28. Wang, Xiao, et al. "Scalable fabrication of printed Zn//MnO2 planar micro-batteries with high volumetric energy density and exceptional safety." *National Science Review* 7.1 (2020): 64-72.

29. Sun, Ke, et al. "3D printing of interdigitated Li-Ion microbattery architectures." *Advanced materials* 25.33 (2013): 4539-4543.

30. Hur, Janet I., Leland C. Smith, and Bruce Dunn. "High areal energy density 3D lithium-ion microbatteries." *Joule* 2.6 (2018): 1187-1201.

31. Lai, Wenhui, et al. "High performance, environmentally benign and integratable Zn//MnO 2 microbatteries." *Journal of Materials Chemistry A* 6.9 (2018): 3933-3940.

32. Liang, Xinyue, et al. "Binder-free CaV3O7 nanobelts with rich oxygen defects as high energy cathode for aqueous Zn-ion battery." *Journal of Power Sources* 472 (2020): 228507.

33. Liang, Xinyue, et al. "Flexible high-energy and stable rechargeable vanadium-zinc battery based on oxygen defect modulated V2O5 cathode." *Nano Energy* 87 (2021): 106164.

34. Lu, Wanheng, et al. "Anion-cation heterostructured hydrogels for all-weather responsive electricity and water harvesting from atmospheric air." *Nano Energy* 104 (2022): 107892.

35. Pan, Xinglong, et al. "Differentiated Ionic Electroresponse of Asymmetric Bio-Hydrogels with Unremitting Power Output." *Advanced Energy Materials* 13.12 (2023): 2204095.

36. Xiao, Xiangting, et al. "Electric-Eel-Type Bi-Ionic Gradient Battery." *ACS Applied Materials & Interfaces* 15.45 (2023): 52641-52650.

37. Han, Won Bae, et al. "Electric Eel-Inspired Soft Electrocytes for Solid-State Power Systems." *Advanced Functional Materials* 34.2 (2024): 2309781.
